# Validation of Differentially Expressed Immune Biomarkers in Latent and Active Tuberculosis by Real-Time PCR

**DOI:** 10.3389/fimmu.2020.612564

**Published:** 2021-03-16

**Authors:** Prem Perumal, Mohamed Bilal Abdullatif, Harriet N. Garlant, Isobella Honeyborne, Marc Lipman, Timothy D. McHugh, Jo Southern, Ronan Breen, George Santis, Kalaiarasan Ellappan, Saka Vinod Kumar, Harish Belgode, Ibrahim Abubakar, Sanjeev Sinha, Seshadri S. Vasan, Noyal Joseph, Karen E. Kempsell

**Affiliations:** ^1^Public Health England, Porton Down, Salisbury, Wiltshire, United Kingdom; ^2^Centre for Clinical Microbiology, University College London, Royal Free Campus, London, United Kingdom; ^3^UCL Respiratory, University College London, Royal Free Campus, London, United Kingdom; ^4^Institute for Global Health, University College London, London, United Kingdom; ^5^Guy’s and St Thomas’ NHS Foundation Trust, London, United Kingdom; ^6^Jawaharlal Institute of Postgraduate Medical Education and Research, Dhanvantri Nagar, Gorimedu, Puducherry, India; ^7^Department of Medicine, All India Institute of Medical Sciences, Ansari Nagar, New Delhi, India; ^8^Department of Health Sciences, University of York, York, United Kingdom

**Keywords:** tuberculosis, biomarker, qPCR, validation, diagnosis, immune

## Abstract

Tuberculosis (TB) remains a major global threat and diagnosis of active TB ((ATB) both extra-pulmonary (EPTB), pulmonary (PTB)) and latent TB (LTBI) infection remains challenging, particularly in high-burden countries which still rely heavily on conventional methods. Although molecular diagnostic methods are available, e.g., Cepheid GeneXpert, they are not universally available in all high TB burden countries. There is intense focus on immune biomarkers for use in TB diagnosis, which could provide alternative low-cost, rapid diagnostic solutions. In our previous gene expression studies, we identified peripheral blood leukocyte (PBL) mRNA biomarkers in a non-human primate TB aerosol-challenge model. Here, we describe a study to further validate select mRNA biomarkers from this prior study in new cohorts of patients and controls, as a prerequisite for further development. Whole blood mRNA was purified from ATB patients recruited in the UK and India, LTBI and two groups of controls from the UK (i) a low TB incidence region (CNTRLA) and (ii) individuals variably-domiciled in the UK and Asia ((CNTRLB), the latter TB high incidence regions). Seventy-two mRNA biomarker gene targets were analyzed by qPCR using the Roche Lightcycler 480 qPCR platform and data analyzed using GeneSpring™ 14.9 bioinformatics software. Differential expression of fifty-three biomarkers was confirmed between MTB infected, LTBI groups and controls, seventeen of which were significant using analysis of variance (ANOVA): CALCOCO2, CD52, GBP1, GBP2, GBP5, HLA-B, IFIT3, IFITM3, IRF1, LOC400759 (GBP1P1), NCF1C, PF4V1, SAMD9L, S100A11, TAF10, TAPBP, and TRIM25. These were analyzed using receiver operating characteristic (ROC) curve analysis. Single biomarkers and biomarker combinations were further assessed using simple arithmetic algorithms. Minimal combination biomarker panels were delineated for primary diagnosis of ATB (both PTB and EPTB), LTBI and identifying LTBI individuals at high risk of progression which showed good performance characteristics. These were assessed for suitability for progression against the standards for new TB diagnostic tests delineated in the published World Health Organization (WHO) technology product profiles (TPPs).

## Introduction

Mycobacterium tuberculosis (MTB), the causative agent of tuberculosis (TB) is the leading cause of infectious disease worldwide (1, 2), accounting for the deaths of approximately 1.3 million people each year (3). The United Kingdom (UK) has seen an increase in TB since the late 1980s, with rates higher than the rest of Europe (4), and there are currently around 6000 new cases each year (5). In 2016, 73.6% of confirmed TB cases in the UK were foreign-born, with India and Pakistan the most frequent countries of origin (6, 7). For India in the same period the estimated incidence of TB was approximately 2.8 million people per year, accounting for about a quarter of the world’s TB cases (8–10) and resulting in considerable mortality (11). Optimal patient care requires early detection, intervention with antibiotic therapy and judicious ongoing management of infectious individuals (8, 12–14). If untreated, each person with pulmonary ATB will infect others at a high rate, on average between 5 and 15 close contacts every year (15).

It is estimated that one quarter of the world’s population are latently infected with MTB (LTBI); approximately 2.3 billion individuals (2). This is an enormous reservoir of people at risk of both spreading TB and developing future disease (16–22). A key priority in TB diagnosis is to predict which of those individuals with LTBI [i.e., with a positive purified protein derivative (PPD) or interferon γ release assay (IGRA)] are in fact still harboring TB bacilli after exposure and are likely to progress to active disease, compared to those who have been exposed and mounted a successful immune response, but cleared the bacilli and are not likely progress to active disease (7, 9, 19, 23–25). Although diagnosis of ATB has been the keystone of the public health response to TB in many countries, including the UK, decreasing the infection reservoir through detection and preventative therapy of LTBI is also essential in achieving disease reduction targets (21, 22, 26–31). There is currently no gold standard method for diagnosing LTBI (32, 33). Identification of individuals with LTBI or incipient ATB (iATB), who are at risk of progression to active disease, but are still relatively asymptomatic is a priority to prevent progression to active disease and to limit disease spread to uninfected individuals (9, 34–36). The LTBI group comprises a heterogeneous group of individuals displaying an immune reaction to PPD mycobacterial antigens (37–39). This represents a spectrum of individuals from those who have completely cleared TB bacilli after exposure or infection, to individuals who are harboring actively replicating, live bacteria in the relative absence of clinical symptoms (incipient active TB (iATB)). These latter individuals are potential reservoirs of infection (40–42) and can spread disease. This is a major problem for control of disease dissemination and LTBI is a key source of infection in high income countries. People with LTBI will often go undiagnosed (14, 40) and are at high risk of progression to active disease. It is predicted that approximately 5% to 10% of individuals with LTBI will progress to ATB during their lifetime (7, 23, 31). The risk of progression from latent to active TB is particularly high among children under the age of 5 years and among people with compromised immunity (1).

As treatment entails risks and costs (43), preventive treatment of LTBI infection should be selectively targeted to the population groups at highest risk for progression to ATB disease, who would benefit most from treatment (9, 34, 44). If caught early enough treatment can be implemented which is less rigorous and results in less severe disease/long term organ damage and fewer relapses (16, 22, 40). Isoniazid monotherapy for 6 months is the primary recommended treatment for LTBI in both adults and children in countries with high and low TB incidence, in contrast to the more intensive combined treatment/DOTS for ATB (13). Non-compliance with anti-mycobacterial therapies contribute to difficulties in disease eradication (25, 45, 46). The treatment for TB is lengthy and patient compliance to long-term drug treatment is varied, with patients often stopping therapy when their symptoms cease (1, 47–50). Failure to complete the treatment regimen promotes the development of multi drug resistance (25, 51–54) and contributes to ongoing barriers for disease eradication (14, 55–57).

The current WHO guidelines for diagnosis and management of TB are outlined as part of their End-TB strategy (2, 58), the primary pillar being diagnosis, as stated in the report “Early diagnosis of TB including universal drug-susceptibility testing, and systematic screening of contacts and high-risk groups”. The report further states that “TB is the 10th leading cause of death worldwide, and since 2007 it has been the leading cause of death from a single infectious agent, ranking above HIV/AIDS. Most of these deaths could be prevented with early diagnosis and appropriate treatment”. The current WHO-endorsed platform for diagnosis of sputum positive TB is the Cepheid GeneXpert, although comprehensive diagnosis still relies on a combination of this with other traditional methods, e.g., chest X-ray and mycobacterial culture from sputum (12). GeneXpert has been widely implemented in many countries globally and has had a positive impact on TB diagnosis and patient management (59–64). However, some high-burden countries like India have reported operational issues with the platform and associated hardware and consumables costs (65, 66) and it not universally available in all high burden countries (34, 67). Its use in India is being recommended for diagnosis of pediatric TB (64, 65). Opportunities for other diagnostic tests to bridge gaps in the current testing portfolio are still evident but will require investment (34, 67, 68). Many TB patients, particularly with EPTB and also LTBI/iATB do not have MTB positive sputum, are consequently harder to diagnose and can further contribute to TB under-diagnosis (12, 21, 33, 69, 70).

Despite considerable investment in research and development for new diagnostics and therapeutics, TB control and eradication has proved challenging (34, 71). Development of rapid, simple and cost-effective diagnostic tests for ATB, particularly EPTB and LTBI are imperative for TB control (72–80). A simple, rapid and cost-effective alternative, which could perhaps be run on a variety of already embedded laboratory platforms and which could diagnose all sub-types of disease is an attractive proposition. Indirect, non-pathogen directed assays employing host immune biomarkers have become the focus of much interest in bridging gaps in the diagnostic portfolio (77, 79–81). These may play an important role in improving primary diagnosis for EPTB (82–88) and LTBI (82, 89–92), assisting clinicians in informing anti-TB treatments and to determine/monitor the response to treatment (14, 83, 87, 91, 93–101). According to Scriba and co-workers a biomarker-based test would reduce incidence by 20% and could reduce over-diagnosis and treatment using methods like IGRA (102), which are poor predictors of disease progression, with pooled positive predictive values of less than 3%.

Numerous studies and reviews have been published evaluating the current status of biomarkers with potential for active, latent and incipient TB diagnosis, many derived from work profiling the host peripheral blood, immune cell transcriptional response (85, 86, 102–106) (82, 90, 93, 94, 96, 101, 107–113). In one of the initial studies Berry and co-workers identified a complex 393 gene panel which could identify individuals with active TB compared with controls and a 86 gene signature which discriminated active TB from other inflammatory and infectious diseases (107). The same group then went on to identify panels which could distinguish pulmonary TB, pulmonary sarcoidosis, pneumonia and lung cancer (114). This field of research has subsequently become a focus of intense interest and these and a number of other groups have identified various discriminatory signatures for the various forms and stages of TB; ATB, EPTB, LTBI and incipient TB and also for exposure in household contacts, risk of progression to active disease and response to therapy (82, 96, 108–113, 115–136). Some of these have subsequently been reviewed or further validated in comparative cohort studies by other workers in the field (88–90, 110, 126, 136–139). Of the previously published blood transcriptional biomarker panels for active pulmonary tuberculosis reviewed recently by Turner et al. (137), four panels achieved the highest diagnostic accuracy and two met the minimum but not optimum WHO target product profiles (TPP) requirements for a triage test (74, 140); Sweeney et al. [Sweeney3 (120)], Roe et al. [Roe3 and BATF2 (119, 121)] (78) and Kaforou et al. [Kaforou25 (132)]. In a similar study by Gupta et al. (89), eight panels showed promise for discrimination of incipient TB with receiver operating characteristic curves ranging from 0·70 to 0·77. These predominantly reflected genes from interferon and tumor necrosis factor-inducible gene expression modules. There is still a need to define biomarker panels which will fulfill the WHO TPP optimal requirements for a triage test and for a confirmatory test.

We have previously shown differential expression of PBL gene mRNAs in response to MTB infection in a *Macaca fascicularis* model of TB (141). These non-human primate models are considered to most closely reflect the disease seen in humans (142, 143) and are widely used, particularly for vaccine development (144–146). Microarray hybridization analyses of macaque peripheral blood mRNAs to human whole genome arrays revealed many temporally expressed, gene expression changes, in response to MTB challenge. A selection of significant, differentially regulated immune mRNA biomarkers was identified, which were shared with previously published human data sets (Patent WO2015170108A1). Here we investigate 72 of the most highly-significant biomarkers by quantitative, real-time PCR (qPCR) in two new cohorts of TB patients and controls from the UK and India. This study was conducted to validate previous findings from the NHP model and confirm biomarker suitability for ongoing diagnostic test development for both ATB (both EPTB and PTB) and LTBI. We discuss the performance of these biomarkers, both singly and in combination with reference to WHO target product profiles and their suitability for inclusion in low complexity qPCR assays. We also present initial observations on the utility of some biomarkers/biomarker configurations to identify LTBI individuals at high risk of progression to ATB and which may differentiate different sub-types of TB, i.e., pulmonary (PTB) and extra-pulmonary (EPTB).

## Materials and Methods

### Study Participants and Sample Collection

All participants recruited to the study were aged ≥18 years old. Patients with PTB and EPTB were recruited at two of India's medical institutes of national importance (1) The All India Institute of Medical Sciences (AIIMS), New Delhi and (2) The Jawaharlal Institute of Postgraduate Medical Education & Research (JIPMER), Puducherry, located in regions of high TB incidence [designated groups IPTB (n = 47) and IEPTB (n = 42)]. Patients with PTB were also recruited at Guy’s and St Thomas’ and Royal Free London NHS Foundation Trusts, London UK (low TB incidence site; designated group UKPTB (n = 63)). Individuals with suspected LTBI (n = 103) and matched negative controls [CNTRLB (n = 102)] were recruited from individuals variably-domiciled in the UK and Asia, resident in the greater London area as part of the UK PREDICT TB study, i.e (4, 42)., by Public Health England Centre for Infections, 61 Colindale Avenue London and University College, London UK. This was a prospective cohort study, recruiting participants from 54 centers in London, Birmingham, and Leicester, at high risk for latent tuberculosis infection (i.e., recent contact with someone with active tuberculosis [contacts] or a migrant who had arrived in the UK in the past 5 years from-or who frequently travelled to-a country with a high burden of tuberculosis [migrants]). Exclusion criteria included prevalent cases of tuberculosis. Individuals with suspected LTBI were identified using the standard Mantoux tuberculin skin test ((TST) i.e., skin-test positive) and/or positivity for one or more of the interferon γ release assay tests (IGRA)—QuantiFERON^®^ TB Gold In-Tube ((QFG) QIAGEN GmbH, Hilden, Germany) and T-SPOT^®^.TB ((TSPOT) Oxford Immunotec Ltd, Oxford, UK). CNTRLB were identified as negative using these test combinations. All patient sample details are given in **Supplementary Table S1** (inside file: **Supplementary Table 1.1**), the number of samples obtained per study site given in **Supplementary Table S1** (inside file: **Supplementary Table 1.2**). Several individuals from the LTBI group were found to have progressed to active disease during study follow up [see **Supplementary Table S1** (inside file: **Supplementary Table 1.3**)]. LTBI were analyzed either as a combined group (LTBI, n = 103) or stratified into non-progressors to active TB (LTBI_NPR, n = 95) or progressors to active TB (LTBI_PR, n = 8) for all ongoing analyses. Other negative controls (CNTRLA, n = 20) were recruited at PHE, Porton Down, Salisbury, UK (Study Number 12/WA/0303).

All patients recruited to the study at partner sites in India were recruited under an approval from the JIPMER Institute Ethics committee (Human studies), AIIMS Institute Ethics committee and PHE, UK (India Study Number JIP/IEC/2015/11/522, UK Study Number PHE0186). The experiments were carried out in accordance with the approved guidelines of the collaborating institutions. Whole blood samples were collected by venipuncture at a single time point in PAXgene™ (PreAnalytiX, SWZ) or Tempus™ Blood RNA tubes (Applied Biosystems, UK) and stored at −80°C until further processing.

### Total RNA Extraction and cDNA Synthesis

Total RNA was extracted from the blood samples of study participants using either the PAXgene Blood RNA or Tempus Spin RNA extraction kits, in accordance with the manufacturer’s instructions. The PAXgene Blood RNA kit (QIAGEN) was used to extract total RNA from all UK group samples and the Tempus Spin RNA Isolation Kit (Applied Bio systems) was used to extract total RNA from all Indian group samples. Although two different RNA extraction methods were used, there are no conflicting reports as to the likely impact of these on the accuracy of downstream qPCR gene target determination (118–123). Differences are reported as relating mainly to miRNAs and not mRNAs (as quantified in this study). To minimize experimental technical variation between samples, mRNA targets were normalized to the average of three internal house-keeping control genes prior to data export and downstream analysis, to minimize any potential sources of technical variation. The concentration and purity of mRNAs were then assessed using a Nanodrop ND-1000 spectrophotometer (Thermo Scientific, EUA). mRNA integrity was further assessed using the Agilent 2100 Bioanalyzer (Agilent Technologies). Purified RNA was immediately processed for complementary DNA (cDNA) conversion using Transcriptor First Strand cDNA synthesis Kit (Roche) as per the instructions provided by the manufacturer. The cDNA was then immediately analyzed using qPCR or stored at −20°C until use.

### Roche Real-Time Ready qPCR Assays

Seventy-two test genes of significance were selected for qPCR validation from our previous studies (141). Details and function of all target genes are given in **Supplementary Table S1** (inside file: **Supplementary Table 1.4**). A summary of the overlap with select previously published gene panels is given in **Supplementary Table S2** (inside files: **Supplementary Tables 2.1** to **2.8**) (genes overlapping with those analyzed in this study highlighted in red text). Glucose-6-phosphate dehydrogenase (G6PD), phosphoglycerate kinase 1 (PGK1) and ribosomal protein L13a (RPL13A) were selected for inclusion as controls from available default control gene options in the Roche Real-Time ready (RTR) assay design center, which showed consistent, invariant expression across control and test groups in the previously published NHP data set.

Expression levels of all human test and control genes were determined using pre-designed or bespoke RTR assays, designed using the RTR assay design configurator (configuration numbers 10059401, 100059386 and 10059377) and arrayed in 384 well format. All qPCR assays were performed in duplicate on the Roche LightCycler 480 (LC480) using TaqMan PCR Probe Master Mix (Roche) and according to the manufacturer’s instructions, using the following cycling conditions (i) preheat for 1 cycle at 95°C for 10 minutes (ii) amplification for 45 cycles: 95°C for 10 s, 60°C for 30 s, 72°C for 1 s (ii) cooling to 40°C for 10 s. Data were normalized to the average of the three control genes prior to export using the LC480 software. Normalized data (ΔCt values) were then exported in .txt file format prior to further analysis.

### Data Analysis and Visualization of qPCR Outputs Using GeneSpring 14.9^™^

Normalized data exported from the Roche LC480 were imported into Microsoft Excel. The mean of two duplicate data points was calculated using the Average $\left(\bar{X}\right)$ function. Averaged data was then imported into GeneSpring 14.9™ (GX14.9) for further statistical and differential gene expression analyses, using baseline transformation to the median of all samples (without further normalization). All data were then assessed for quality and filtered by error, where the % coefficient of variance (%CV) was >200 (maximizing the number of entities exhibiting expression differences across all samples and removing those with poor or no signals). Statistically significant features were identified using either one-way analysis of variance (ANOVA) analysis using Benjamini-Hochberg false discovery rate (BH FDR, at a corrected p-value cutoff p < 0.05) across all groups, or t-tests for comparisons between individual groups (also using BH FDR and a cutoff p < 0.05). All further analyses and graphical depiction of data outputs were conducted using other functions in GX14.9 using default settings, e.g., scatter plot, regression and unbiased hierarchical cluster analyses (either Euclidean (EUC) or Pearson’s centered (PC) distance metrics using Ward’s linkage rule and the cluster entities setting). Other data analyses were conducted using various functions in “R”, Microsoft Excel or Sigmaplot 12.0 (Systat Software Inc.).

### Receiver Operating Characteristic/Area Under the Curve and Performance Analysis

Receiver Operating Characteristic/area under the curve (ROC/AUC) analyses were performed using normalized, exported mean LC480 qPCR ΔCt values. ROC curves were plotted using “R” × 64 3.4.0 Software using the ROCR package or the ROC analysis tool of Sigmaplot 12.0. The accuracy and performance of each candidate single biomarker was measured by calculating area under the curve (AUC) values. Cutoff values were predicted by measuring the optimal accuracy of the curve, from which the sensitivity and specificity of each biomarker/biomarker panel test were determined. Optimal cutoffs were selected to obtain best sensitivity and specificity and to compare biomarker performance. Combined panels of biomarkers were also assessed to determine whether these could show improved discrimination between control and infected TB groups over single biomarkers. Simple algorithms consisting of biomarkers combined additively were assessed by ROC analysis and the diagnostic performance further assessed using sensitivity, specificity, cutoff values, likelihood ratios and positive (PPV) and negative (NPV) predictive value calculations. Select biomarker panel configurations were also evaluated to WHO TPP requirements for triage minimum and optimum and confirmatory test minimum requirements using the Sigmaplot 12.0 ROCR/ROC analysis functions. Outputs were depicted graphically using either Sigmaplot 12.0 or GraphPad 8.0.

### Receiver Operating Characteristic/Area Under the Curve and Performance Analysis for Optimal Biomarker Panels on Previously Published Data Sets

Previously published data sets from Singhania et al. [GSE107991, GSE107992, GSE107993, GSE107994 (109)], Leong et al. [GSE101705 (110)], Turner et al. [E-MTAB-8290 (137)], and Zak et al. [GSE79362 (111)] studies were downloaded and normalized numeric expression values for the relevant panel gene entities extracted. These were analyzed for ROC/AUC and overall performance to WHO TPP requirements for select, significant composite gene biomarker panels from this study as described above (section 2.5).

## Results

### Quality Assessment of Normalized Data Signals and Cluster Analysis

Normalized, exported mean Roche Lightcycler qPCR ΔCt values were imported into GX14.9 and assessed for signal quality. Fifty-three of seventy-two gene entities remained after filtering by error (%CV >200). Samples were assigned to their specific control and disease groups, i.e., (i) low TB incidence region UK control (CNTRLA) (ii) low TB incidence region UK control from the PREDICT TB study (CNTRLB) (iii) low TB incidence region UK LTBI from the PREDICT TB study (LTBI), variously stratified according to progression (LTBI_PR) or non-progression (LTBI_NPR) to ATB (iv) low TB incidence region UK TB (UKPTB) (v) high TB incidence region India extra-pulmonary TB (IEPTB) (vi) high TB incidence region India pulmonary TB (IPTB).

**Figure 1:**
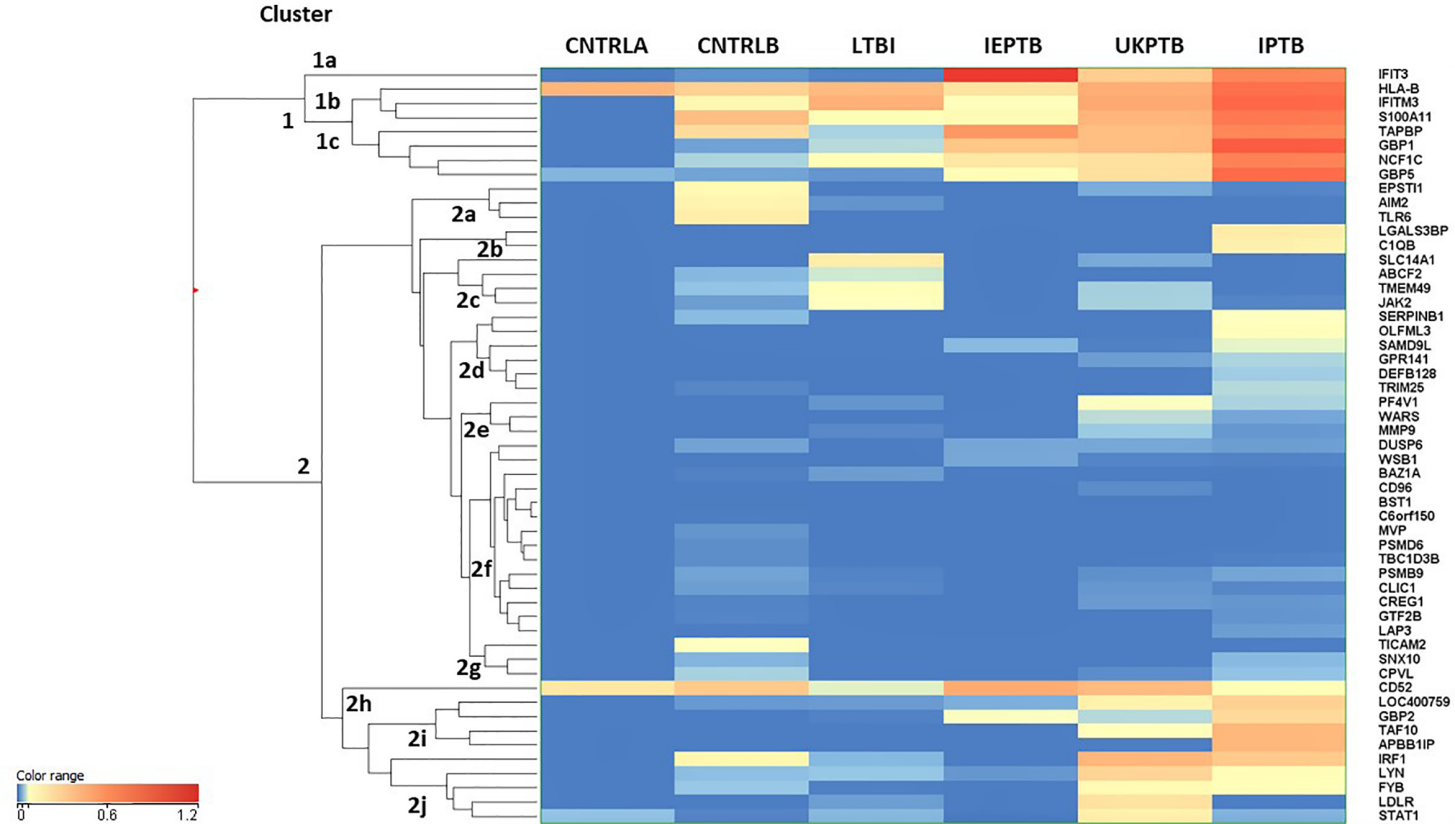
Cluster analysis on all fifty-three significant, filtered entities on group averaged data CNTRLA - low TB incidence region UK control group CNTRLB - low TB incidence region UK control group from the PREDICT TB study group LTBI - low TB incidence region LTBI from the PREDICT TB study group IEPTB - high TB incidence region extra-pulmonary TB group UKPTB - low TB incidence region UK TB group IPTB - high TB incidence region Indian pulmonary TB group.

An unbiased EUC cluster analysis was then performed on filtered data, the results are given in **Figure 1**. Two clear main clusters of entity expression could be seen using this analysis; clusters 1 and 2, with associated sub-clusters. Overall, there was an observed pattern of increasing differential regulation of biomarkers in the TB disease groups compared with the control groups, from LTBI through IEPTB, UKPTB and IPTB. The composition of biomarkers varied slightly in the comparisons between groups, although there was also some overlap of entity expression between groups. Gene entities in cluster 1 appeared to delineate groups associated with generalized presumed exposure and/or infection with MTB. Cluster 2 and associated sub-cluster gene entities exhibited variable expression between exposed or infected groups, clusters 2i and 2j featured entities which associated more strongly with ATB. CD52 (cluster 2h) appears more generically differentially expressed across the groups, but slightly down-regulated in LTBI.

Cluster 1 includes only eight entities, some of which are interferon regulated, e.g., IFIT3 and GBP1, others include entities associated with MHC class I antigen processing, e.g., HLA-B and TAPBP and associated with neutrophil and/or other innate immune cell activity, e.g., IFITM3, S100A11 and NCF1C; (i) in cluster 1a, IFIT3 is only associated with the ATB disease groups (ii) cluster 1b the entities associate mainly with the high incidence control (CNTRLB) and ATB groups, although HLA-B also appears expressed in the low incidence (CNTRLA) group and (iii) cluster 1c, the entities associate with the LTBI, IEPTB, UKPTB and IPTB groups (**Figure 1**).

Cluster 2 featured immune-related entities which were differentially regulated between sub-groups, (i) cluster 2a with the CNTRLB group, (ii) clusters 2b, 2d, and 2j with the IPTB group, (iii) cluster 2c with the UKPTB group, (iv) cluster 2e predominantly with the UKPTB group, (v) cluster 2f weakly with the CNTRLB, UKPTB and IPTB groups and (vi) cluster 2g with the CNTRLB and more weakly with the IPTB group, (vii) cluster 2h associated across all groups but more weakly with the LTBI and IPTB groups, (viii) cluster 2i with the IEPTB, UKPTB and IPTB groups and (ix) cluster 2j with the CNTRLB, LTBI, UKPTB and IPTB groups (i.e., all test groups except the IEPTB group). Thus, good differential expression of gene entities was observed between the low TB incidence controls (CNTRLA) and the other groups, i.e., those with ATB from low TB (UKPTB) and high TB incidence regions (IEPTB and IPTB).

### Analysis of Normalized qPCR Data Using Analysis of Variance

To determine the best performing biomarkers for onward progression from those displaying a positive signal post-filtration, Analysis of Variance (ANOVA) was performed across all groups using BH FDR (corrected p value < 0.05) and using the Student–Newman–Keuls differences in means (SNK), post-hoc test. Seventeen of the fifty-three gene entities from the %CV filtered data set were found to be statistically significant and differentially regulated across the groups using this analysis; including CD52, GBP1, GBP2, GBP5, HLA-B, IFIT3, IFITM3, IRF1, LOC400759 (GBP1P1), NCF1C, PF4V1, S100A11, SAMD9L, STAT1, TAF10, TAPBP, and TRIM25 (17-plex signature). The number of entities that were discriminatory between groups from the ANOVA SNK analysis are summarized in **Supplementary Table S1** (inside file: **Supplementary Table 1.5**) and **Supplementary Information S1, Figure 1.1**.

### Cluster and Scatterplot Analysis of Significant Differentially Regulated Entities

To further investigate group-specific changes in the seventeen, statistically significant differentially regulated biomarkers, PC unbiased cluster analysis was performed a cross the control, stratified LTBI (LTBI_NPR and LTBI_PR) and other ATB disease groups (**Supplementary Information S2, Figure 2.1** and ANOVA p- and fold change values in **Supplementary Table S3** (inside files: **Table 3.1** with pairwise, p values from the SNK post hoc test table given in **Table 3.2**). individual line plots (average expression +/− standard error) for each of these entities are given in **Supplementary Information S1, Figures 1.2 to 1.18**.

Two distinct clusters were observed, each of which could be divided into four sub-clusters, which further delineate differential expression of the key biomarkers between the control and TB-exposed or infected groups. In addition, clear differences in expression could be seen between the LTBI progressors and non-progressors for a number of these gene biomarkers (boxed in red).

**Figure 2:**
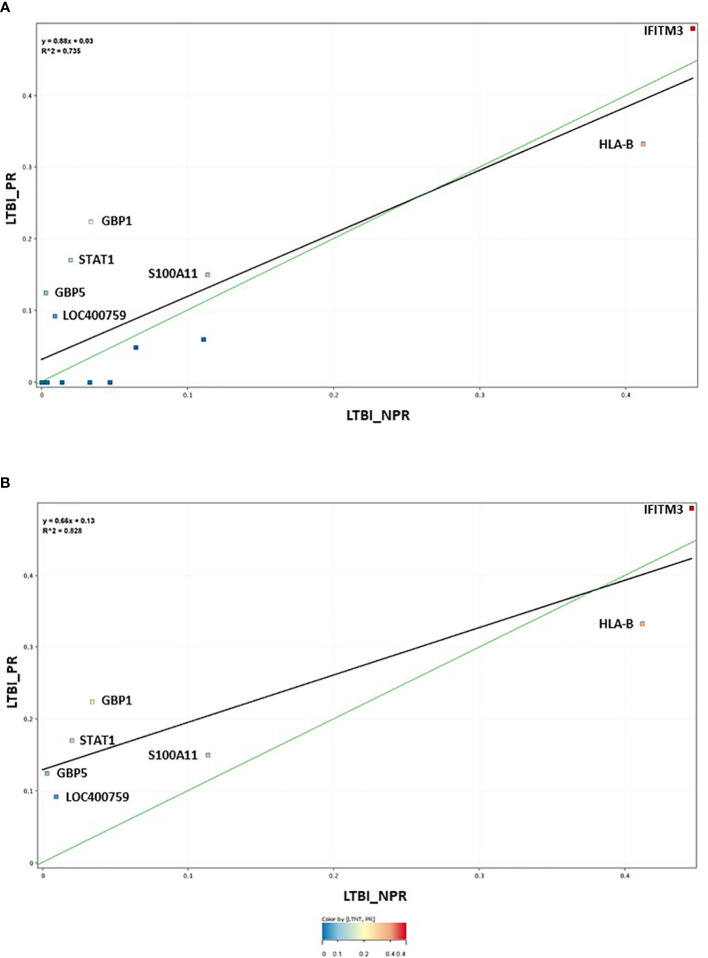
**(A)** Scatter graph depiction of the seventeen statistically significant entities in LTBI non-progressor and LTBI progressor groups, with associated linear regression (R^2^) significance analysis **(B)** Scatter graph depiction of the seven preferred, differentially expressed entities in LTBI non-progressor and LTBI progressor groups, with associated linear regression (R^2^) significance analysis.

The 17-plex signature was further analyzed in greater detail using scatter plot analysis (**Figure 2**), for the LTBI_NPR and LTBI_PR groups. Seven of these biomarkers showed clear differential expression between the two groups (**Supplementary Information S2, Figure 2.1**, with fold change differences given in **Supplementary Table S3** (inside file: **Table 3.3**). IFITM3, S100A11, GBP1, GBP5, STAT1 and LOC400759 (GBP1P1), were upregulated in the LTBI-PR group and HLA-B, TAPBP, NCF1C, PF4V1, CD52 and IRF1 were downregulated. Regression analysis using the 17-plex signature gave a best fit line R^2^ value of 0.735 (**Figure 2A**), however using the six upregulated biomarkers plus HLA-B (7-plex signature) alone, the R^2^ value increased to 0.828 (**Figure 2B**). Addition of any other differentially markers to the panel did not provide any further improvement to the R^2^ value. These showed therefore good potential for identifying “high risk” pre-progressor LTBI patients at an early stage of disease for preventative interventions.

### Analysis of Control and LTBI Groups Using 7plex Cumulative Average Expression

Inherent variability in biomarker expression was observed between individuals within all the groups (depicted in heatmap format in **Supplementary Information 4, Figure 4.1**) and particularly control group CNTRLB. Some individuals within this group have high positivity for select key biomarkers. Using the normalized numeric ΔCt values for the 7-plex signature, we assessed whether these markers could provide a means for stratifying individuals in the control, LTBI_NPR and LTBI_PR groups into high, medium and low risk categories, using a simple arithmetic cumulative index (**Supplementary Table S3** (inside file: **Table 3.4**). ROC curve analyses were not conducted due to an imbalance in the number of individuals in the control and LTBI_NPR groups, relative to the LTBI_PR group (n = 8) **Supplementary Table S3** (inside files: **Table 3.1** with pairwise, group t-test p values given in **Table 3.2**). Individuals in these groups were ranked according to their cumulative average expression values (CAE) for the 7-plex signature, then cutoff points set at (i) equal to and greater than the mean $\left(>\bar{X}\right)$ (ii) equal to and greater than the mean plus one standard deviation $\left(\bar{X}+\text{SD}\right)$ or (ii) equal to and greater than the mean plus two standard deviations $\left(\bar{X}+2\text{SD}\right)$. Three LTBI_NPR, two LTBI_PR, four CNTRLB (including patients 1053, 2439 and 1864 highlighted in **Supplementary Information 4, Figure 4.1**) had cumulative values over $\bar{X}+2\text{SD}$. No CNTRLA individuals had cumulative values over $\bar{X}+2\text{SD}$. Ten LTBI_NPR, one LTBI_PR, ten CNTRLB and no CNTRLA individuals had cumulative values between $\bar{X}+2\text{SD}$ and $\bar{X}+\text{SD}$. Twenty-nine LTBI_NPR, 1 LTBI_PR, 16 CNTRLB and 6 CNTRLA individuals had cumulative values greater than $\bar{X}$, but less than $\bar{X}+\text{SD}$. These results would suggest that both the CNTRLB and LTBI groups are heterogeneous. The CNTRLB group may be a mixture of true negative, exposed uninfected and LTBI infected and the LTBI group a mixture of exposed (currently) uninfected and exposed (currently) infected. Those in the upper ranges for the CAE in both groups are potentially at higher risk of progression to ATB. However, further work needs to be done to assess the performance of this 7-plex panel for stratification purposes, with a greater number of patients and controls to follow up.

These results suggest that the TST and IGRA tests used to define these groups may have incorrectly assigned some individuals in the CNTRLB and LTBI groups. There were near equivalent numbers in each stratified category and the groups look similar in ranked distribution. This information further suggests use of individual or low-complexity gene biomarker panels will be unlikely to be sufficient for stratification of LTBI and high-risk control groups, due to inherent variabilities in expression between individuals, which may lead to omissions in identifying “true” infected individuals. A more complex multi-biomarker approach will be required to give requisite test sensitivity.

### Determination of Single Biomarker Receiver Operating Characteristic Profiles

Pairwise comparisons for all seventeen significant differentially-expressed single biomarkers were conducted across all infected and control groups [**Supplementary Table S3** (inside files: **Table 3.5, 3.5A** for controls vs ATB, **3.5B** for controls vs LTBI and **Table 3.5C** for controls&LTBI vs ATB)], ranked according to specificity). The accuracy of single biomarker discriminatory performance across the main active TB disease groups is summarized in **Supplementary Table S1** (inside file: **Supplementary Table 1.6**) and between the LTBI_PR and LTBI_NPR groups in **Supplementary Table S1** (inside file: **Supplementary Table 1.7**). Many of these single biomarkers gave AUC values above 0.9, the cutoff considered to be an indicator of very high accuracy (up-regulated in the test group, highlighted in bold black text and dark grey fill). Others gave AUC values above 0.8, considered to be an indicator of high accuracy (highlighted in normal text and medium gray fill). Many others gave AUC values above 0.7, the cutoff considered to be an indicator of moderate accuracy (highlighted in normal text and light grey fill). Some gene biomarkers gave AUC curve values below a cutoff of 0.3 indicating an inverse relationship of the markers between the control and test groups ((i.e., down-regulated in the test group) highlighted in white italic text and very dark grey fill). From these analyses, many of the significant gene biomarkers were observed to show good performance between disease and control groups. The best performing across all groups were GBP1, GBP2, IFIT3 and SAMD9L (up-regulated) and TAF10 (down-regulated). Several others with more moderate or group-specific performance were also considered viable candidates for ongoing diagnostic algorithm development. IFITM3 showed the best performance in delineating the LTBI group from both groups of controls and IRF1, TAPBP, and TRIM25 may highlight subtle differences in expression between the two LTBI groups.

Performance/accuracy and discrimination between control and disease groups were assessed for likelihood ratios (LR) and positive/negative predictive values (PPV/NPV), using defined qPCR thresholds. Cutoff values which discriminated all ATB from controls were selected at a fixed sensitivity of 80% for PPV and NPV calculations. The accuracy and discriminatory performance between control and disease groups was very good for many biomarkers. Using predicted cutoff values at 80% sensitivity, the LR+ values approached 10 and LR- were correspondingly low. GBP1 attained a specificity of 91.8% at 80% sensitivity and good PPV/NPV (92.42% and 78.87% respectively) when discriminating all ATB from all controls, IFIT3 also showed good performance with a specificity of 90.98% at 80% sensitivity (PPV/NPV; 90.98% and 78.57% respectively). These results suggest both biomarkers would be very good diagnostic candidates for ATB.

GBP1 and IFIT3 showed less impressive performances for LTBI. GBP1 attained a specificity of 61.68% at 80% sensitivity and PPV/NPV (72.53% and 68.84% respectively) and IFIT3 37.38% specificity at 80% sensitivity (PPV/NPV; 69.69% and 57.14% respectively). The best performing marker for discrimination of LTBI from all controls was IFITM3, with a specificity of 64.49% at 80% sensitivity and PPV/NPV (74.19% and 72.06% respectively). These results emphasize again the difficulties in discriminating LTBI from controls, compared with the superior performance of select biomarkers for the ATB group and suggest that the use of individual gene biomarkers would be unlikely to be sufficient for primary disease diagnosis, due to somewhat lower NPV values. This may lead to omissions in identifying infected individuals, due to the likelihood of false negatives and to some lesser extent false positives with individual biomarkers. It was decided therefore to investigate a multi-biomarker panel approach for on-going diagnostic development.

### Determination of Biomarker Panel Receiver Operating Characteristic Profiles and Determination of Diagnostic Algorithms for Diagnostic Test Development

To improve the overall sensitivity and performance, various combinations of gene biomarkers were trialed to determine the optimal configuration to distinguish the various TB disease groups (LTBI (both LTBI_NPR and LTBI_PR), IEPTB, UKPTB, and IPTB) from the control groups (CNTRLA and CNTRLB), with a view to identifying diagnostic panels. qPCR values were combined or subtracted additively according to empirically designed algorithms, then tested using pairwise ROC curve analyses. Illustrations of the best performing combinations are given in full in **Supplementary Table S3** (inside files: **Table 3.6, 3.6A** for controls vs ATB, **3.6B** for controls vs LTBI and **Table 3.6C** for controls&LTBI vs ATB). These are summarized in **Table 1** across the main active TB disease groups and **Table 2** across the LTBI_PR and LTBI_NPR groups respectively.

**Table 1:** Summary of AUC ROC values for control and ATB group pairwise comparisons using simple, composite arithmetic algorithms.

Group/Algorithm	Biomarker ROC Curve Value				
	GBP1+GBP2 +IFIT3+SAMD9L+TAPBP	GBP1+ GBP2+IFIT3 +SAMD9L	GBP1+ IFIT3 +SAMD9L	GBP1+GBP2 +IFIT3	GBP1+IFIT3
**CNTRLA vs IEPTB**	**0.969**	**0.981**	**0.981**	**0.985**	**0.981**
**CNTRLA vs UKPTB**	**0.945**	**0.952**	**0.937**	**0.962**	**0.956**
**CNTRLA vs IPTB**	**0.977**	**0.970**	**0.977**	**0.982**	**0.980**
**CNTRLB vs IEPTB**	**0.911**	**0.951**	**0.958**	**0.951**	**0.957**
**CNTRLB vs UKPTB**	0.870	**0.906**	**0.915**	**0.907**	**0.916**
**CNTRLB vs IPTB**	**0.938**	**0.949**	**0.959**	**0.947**	**0.956**
**CNTRLA vs All ACTIVE TB**	**0.962**	**0.968**	**0.961**	**0.982**	**0.970**
**CNTRLB vs All ACTIVE TB**	**0.902**	**0.932**	**0.940**	**0.931**	**0.940**
**CNTRLA&CNTRLB vs All ACTIVE TB**	**0.911**	**0.938**	**0.944**	**0.939**	**0.945**

**Table 2:** Summary of AUC ROC values for control, latent and combined ATB group pairwise comparisons using simple, composite arithmetic algorithms.

**Group/Algorithm**	**ROC/AUC Values for Group Pairwise Comparisons using Composite** **Biomarker Panel Gene Algorithms**			
	**GBP1+ IFITM3**	**GBP1 + IFIT3**	**GBP1+ IFIT3 + IFITM3**	**GBP1+IFITM3+ S100A11**
**CNTRLA vs LTBI-NPR**	**0.960**	0.853	**0.966**	**0.987**
**CNTRLA vs LTBI-PR**	**0.981**	0.806	**0.975**	**0.987**
**CNTRLA vs LTBI**	**0.961**	0.849	**0.967**	**0.994**
**CNTRLB vs LTBI-NPR**	0.777	0.7815	0.776	0.590
**CNTRLB vs LTBI-PR**	0.799	0.734	0.793	0.592
**CNTRLB vs LTBI**	0.779	0.778	0.777	0.590
**CNTRLA&CNTRLB vs LTBI**	0.809	0.790	0.808	0.655
**CNTRLA&CNTRLB vs LTBI-NPR**	0.807	0.793	0.807	0.655
**CNTRLA&CNTRLB vs LTBI-PR**	0.829	0.746	0.823	0.658
**LTBI-NPR vs IPTB**	0.792	0.909	0.814	0.830
**LTBI -PR vs IPTB**	0.753	0.838	0.79	0.795
**LTBI VS ALL ACTIVE TB**	0.629	0.865	0.685	0.711

**Figure 3:**
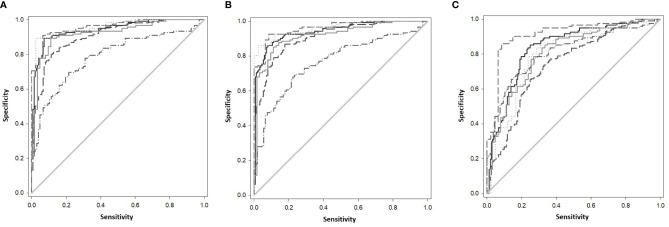
**(A)** ROC Curve analysis of all individual TB disease groups compared to the CNTRLA group and combined CNTRL&LTBI vs ATB groups, using the GBP1+IFIT3 algorithm **(B)** ROC Curve analysis of all individual TB disease groups compared to the CNTRLA group and combined CNTRL&LTBI vs ATB groups, using the GBP1+IFIT3 +SAMD9L algorithm **(C)** ROC Curve analysis of all individual TB disease groups compared to the CNTRLA group and combined CNTRL&LTBI vs ATB groups, using the GBP1+IFITM3 algorithm All CNTRLS vs ATB 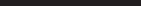, All CNTRLS vs IPTB 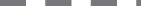, All CNTRLS vs UKPTB 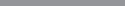, All CNTRL vs IEPTB 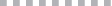, All CNTRL vs LTBI 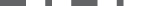, All CNTRL&LTBI vs ATB 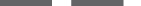.

**Figure 4:**
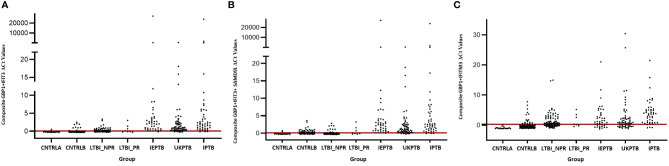
Scatter plot representations of data from analyses using the GBP1+IFIT3, GBP1+IFIT3 +SAMD9L and GBP1+IFITM3 algorithms across all control and TB disease groups including the LTBI non-progressor and progressors **(A)** GBP1+IFIT3 algorithm using the calculated cut-off value which discriminates ATB from all combined control groups (-0.046). **(B)** GBP1+IFIT3 +SAMD9L algorithm using the calculated cut-off value which discriminates ATB from all combined control groups (-0.036) **(C)** GBP1+IFITM3 algorithm using the calculated cut-off value which discriminates the LTBI from all combined control groups (0.074).

The gene combinations which gave most consistent high accuracy discrimination between all control and ATB groups were GBP1+IFIT3 (ROC/AUC = 0.945, **Figure 3A** and depicted in scatterplot format in **Figure 4A**). Inclusion of SAMD9L (GBP1+IFIT3+SAMD9L) reduced the AUC value slightly (ROC/AUC = 0.944, **Figure 3B** and **Figure 4B**), but increased the specificity and PPV and NPV values, suggesting combining these three biomarkers may give the best overall test performance. This latter 3-plex gene combination also worked reasonably well for discrimination of Control&LTBI vs ATB groups (**Supplementary Table S3** (inside file: **Table 3.6C**). The distribution of individuals above the defined cutoffs for each of these two combinations seen in **Figures 4A**, **B** show the high accuracy discrimination across all three ATB groups, compared with the CNTRLA group. The UKPTB group shows a greater range of positive and negative results above and below the cutoff value (−0.46) and may be more heterogeneous. At this cutoff only three IPTB patients and one IEPTB appear as false negatives. More moderate accuracy discrimination was observed between the LTBI and both control groups at the same cutoff (AUC = 0.79). Similar results were shown for GBP1+IFIT3+SAMD9L (**Figure 4B**). As discussed above the combination of GBP1+IFITM3 improved the ability to discriminate LTBI from combined controls but not from ATB (**Figures 3C** and **4C**).

**Figure 5:**
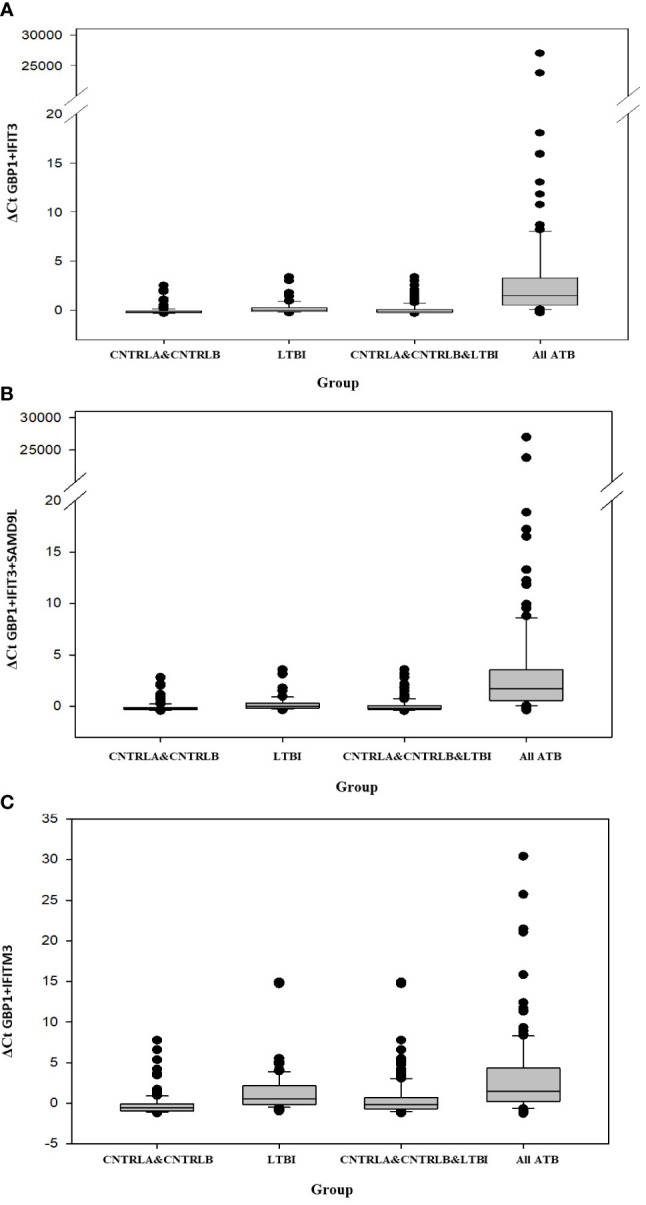
Bar chart representation of the comparison between the combined CNTRLA and CNTRLB control groups, the LTBI group, the combined CNTRLA, CNTRLB and LTBI groups and the combined ATB groups **(A)** using the GBP1+IFIT3 algorithm (from **Table 2**), **(B)** using the GBP1+IFIT3+SAMD9L algorithm (from **Table 2**) **(C)** using the GBP1+IFITM3 algorithm (from **Table 3**).

Other combination panels of gene biomarkers were trialed to determine the optimal configurations distinguishing LTBI (LTBI_NPR and LTBI_PR) from control groups (see **Table 2** and **Supplementary Table S3** (inside file: **Table 3.6B**)). GBP1 + IFITM3 showed high accuracy for discrimination of the LTBI groups from the CNTRLA group (AUC = 0.96) and more moderately when including the CNTRLB group (AUC = 0.809), (**Figures 3C** and **4C**). GBP1+IFIT3 showed best performance for discriminating LTBI from ATB (**Table 2**) with an AUC of 0.865. Small differences were detected between non-progressor and progressor groups. Additionally, the combination of GBP1 + IFIT3 + IFITM3 achieved an ROC/AUC = 0.808, showing this combination could also be used for discrimination of LTBI and ATB. The ability to discriminate ATB from controls with or without LTBI was shown for all three panel combinations (shown in boxplot format in **Figures 5A–C**).

Diagnostic accuracy was further assessed using fixed sensitivities and/or specificities for the WHO TPPs (74, 140) for triage or confirmatory tests (given in **Supplementary Table S3** (inside file: **Table 3.7**) — TB triage test; minimum ≥ 90% sensitivity and 70% specificity, TB optimal test; ≥ 95% sensitivity and 80% specificity, TB confirmatory test; ≥ 98% specificity and 65% sensitivity), with a side by side comparison of the panels’ performance given in **Supplementary Table S3** (inside file: **Table 3.8**). The combination of GBP1+IFIT3 met the minimum and optimal triage and the confirmatory test requirements for all pairwise comparisons using CNTRLA alone, but not with CNTRLB alone or when CNTRLB was combined with CNTRLA, with the exception of the EPTB group. GBP1+IFIT3+SAMD9L met the minimum and optimal triage and the confirmatory test requirements for all pairwise comparisons using CNTRLA alone, except the UKPTB group. However, it showed good performance for the CNTRLB and CNTRLA&B combinations vs all ATB for the confirmatory test performance.

### Determination of Biomarker Panel Receiver Operating Characteristic Profiles and Performance of the Optimal Biomarker Panels on Previously Published Data Sets

The performance of the panels on previously published data sets was then conducted [**Supplementary Table S4**, (inside **Table: 4.1**)]. Overall the performance of the panels was good, with high ROC/AUC values, however the results were variable. Most of the panels met either the minimal triage or confirmatory test requirements, except for the GSE107993 (Singhania_Leicester LTBI non-progressor study set), GSE79362 and E-MTAB-8290 data sets, where there was no minimal requirement positivity observed. Several of the preferred biomarker combinations met the minimum triage requirements for many of the study sets, including GSE107994 (Singhania_Leicester LTBI progressor study set). GBP1+GBP2+IFIT3+SAMD9L also met the confirmatory test minimum for controls vs ATB. The 7plex signature met the minimum and optimum triage and confirmatory test requirements for controls vs ATB and LTBI vs ATB and the minimum requirements for controls vs LTBI progressors. The GSE107992 data set showed good performance for most of the panels, except the optimal triage requirement for GBP1 + IFIT3, GBP1 + IFIT3+ SAMD9L and GBP1 + GBP2 + IFIT3 + SAMD9L +TAPBP.

## Discussion

Here we describe Roche LightCycler 480 qPCR validation of differentially-regulated whole blood PBL mRNA gene biomarkers, previously identified in an NHP model of pulmonary TB (141), in 2 cohorts of patients with active TB (ATB) and a cohort of patients with latent TB (LTBI), compared with two groups of controls (CNTRLA and CNTRLB). Determination of candidate biomarker expression was conducted across ATB patient groups with pulmonary TB (UKPTB and IPTB), extra-pulmonary TB (IEPTB) and latent TB (LTBI_NPR and NPR_PR). Fifty-three of seventy-two biomarkers showed differential gene expression signals between disease groups and controls after quality filtration (%CV >200), on this platform. Seventeen highly significant markers were identified from this filtered data set using ANOVA; CALCOCO2, CD52, GBP1, GBP2, GBP5, HLA-B, IFIT3, IFITM3, IRF1, LOC400759 (GBP1P1), NCF1C, PF4V1, SAMD9L, S100A11, TAF10, TAPBP, and TRIM25 were further analyzed. The results showed a predominance of interferon-regulated gene entities, i.e., IRF1, STAT1, IFIT3, IFITM3, GBP1, LOC400759 (GBP1P1), GBP2, GBP5, and TRIM25 along with other entities associated with immune function. Using unbiased cluster analysis, the significant markers showed differential expression profiles across the control and study groups and increasing patterns of expression in active disease groups. Involvement of interferons and dysregulation of interferon-regulated genes in TB has been documented extensively elsewhere (101, 105, 107, 147–151), and our study further confirms these observations. Some inferences as to the underlying biology of biased expression across the groups could be made (a fuller description of gene biological function and group specific expression is given in **Supplementary Table S1** (inside file: **Supplementary Table 1.4** and **Supplementary Information S4**). Gene expression patterns may suggest some phased expression of interferon-regulated genes associated with different stages of disease.

ROC analyses revealed the single best performing biomarkers for discriminating both ATB and LTBI groups. Individual best performing biomarkers were then assessed for performance in combination using simple algorithms with the aim of developing minimal, multiplex biomarker panels for diagnosis. Various combinations were trialed empirically, with smaller two and three multiplexes giving good performance characteristics. The panels have shown good sensitivity, specificity and PPV/NPV. Combinations of GBP1, IFIT3, IFITM3 and SAMD9L using simple arithmetic algorithms looked promising for diagnosis of most ATB presentations. They may also be useful for diagnosis of LTBI and identification of individuals at high risk of progression.

The key diagnostic panel for all types of ATB was determined to be GBP1 and IFIT3, which gave the best performance both individually and in combination (combined AUC = 0.95). The combination of GBP1+IFIT3 could also discriminate LTBI samples from controls with a fairly good degree of accuracy (combined AUC = 0.79), but with reduced resolution compared with the preferred combination of GBP1 + IFITM3 (combined AUC = 0.809). The combination of GBP1 and IFIT3 met both the minimum and optimum TTP profile criteria for both the triage and confirmatory test when single and combined ATB groups were compared with the CNTRLA group. When the CNTRLB group was used as comparator this combination met the minimum triage test criteria only for the UKPTB, IPTB and IEPTB groups and the combined ATB group. It met the minimum criteria for the confirmatory test for the IEPTB and IPTB, but not the UKPTB or combined ATB groups. When the CNTRLA and CNTRLB groups were combined and the ATB groups then compared, this combination met the minimum triage criteria for all single ATB groups and the combined ATB group, but the optimal criteria for the triage test for the IEPTB group and the minimum confirmatory test for the IEPTB and IPTB groups only. When the CNTRLA, CNTRLB and LTBI groups were combined and compared with the single and combined ATB groups, this biomarker combination met the minimum criteria for the triage test only. These results demonstrated the potential for this minimum biomarker set to be used as both a triage and confirmatory test; however, its performance is influenced by the comparator group used. The results show clear differences between the control groups, again suggesting the CNTRLB group is a biased comparator, due to the likely presence of TB positive individuals. If these tests were used in an unbiased fashion, a proportion of the samples in the CNTRLB and LTBI groups would flag up as positive above the assigned threshold test cutoffs and be identified for potential follow up. This biomarker combination was not useful for discrimination of any combination of control or LTBI groups.

Inclusion of SAMD9L (GBP1+IFIT3 +SAMD9L) achieved a reduced AUC value of 0.94 but improved sensitivity and positive and negative predictive values (PPV & NPV), suggesting that this combination could give overall best performance (i.e., reducing the number of false negatives). This combination met many of the minimum and optimum TTP profile criteria for both the triage and confirmatory test, similarly to GBP1 and IFIT3, but its performance did not compare as favorably for the confirmatory test, except for all ATB groups vs CNTRLB singly as comparator and in combination with CNTRLA. Similar results were also observed with the other combinations, some of which show improved performance for discriminating between LTBI and ATB for the minimum triage requirement. It can be seen that the various biomarker combinations give slightly different results and any resulting developed test could potentially be tailored according to intended end use, particularly for discrimination between LTBI and ATB.

GBP1+ IFITM3 met the minimum and optimal performance criteria for the LTBI_NPR and combined LTBI groups for the triage test in comparison with CNTRLA, but the minimum criteria for the LTBI_PR group only. It met the confirmatory test criteria for the LTBI_PR group, but not for the LTBI_NPR and combined LTBI groups. These results are likely to be influenced by differences between LTBI and the two different control groups, due to heterogeneity in the CNTRLB group, which may contain mis-assigned LTBI or preclinically infected individuals, as discussed previously. The GBP1+ IFITM3 panel distinguishes LTBI from unambiguously uninfected negative control groups, with good sensitivity and thus be useful as a rule out test. It may also pick up previously unidentified LTBI classified negative using the Mantoux or IGRA tests. However, more complex multiplex assays may be required for high confidence detection of LTBI and asymptomatic pre-progressor TB patients at a relatively early, latent stage of disease, due to high inherent variability between individuals in the control and LTBI groups and also a relatively low level of biomarker gene expression in these individuals compared with those in the ATB groups. Various combinations of GBP1, IFITM3, GBP5, HLA-B, LOC400759, S100A11 and STAT1 may be useful for LTBI primary diagnosis and stratification, however this requires further study.

There have been a significant number of comparative studies investigating various biomarkers/biomarker panels for MTB diagnosis (88–91, 97, 110, 137, 138, 152–155). Some of the biomarkers validated in this study have been identified by other workers in the field previously as highly useful key components of other TB-diagnostic panels, e.g., GBP1, GBP2, LOC400759 (GBP1P1), GBP5, STAT1, IFIT3 & IFITM3 (110, 114, 115), adding confidence to our own observations. The overall view that this is a valid approach and a productive pipeline for new diagnostic test development, as evidenced in published market evaluation reports (60, 75–77, 79, 81, 85, 87, 155–157). However, to date few have been postulated to fulfill the WHO minimum requirements for progression (89, 110, 137). GBP1 and IFITM3 have been previously reported as components of a four-gene signature from Maertzdorf et al. for discrimination of TB infected from healthy individuals (123). This panel was included as part of prior signature evaluation studies by Leong et al (110, 138). They showed that both complex and relatively simple biomarker combinations, could be useful diagnostically and that some of the smaller panels evaluated previously exhibit good performance characteristics. These would be more amenable to simple, cost-effective assay development. Turner and co-workers also evaluated a number of previously published biomarker signatures to benchmark their diagnostic accuracy against the WHO TPPs for a tuberculosis triage test and found none which met the optimum criteria and two which met the minimum criteria, Roe3 and Sweeney3. These did not meet the minimum requirement for a confirmatory test (89, 137). Our study may offer biomarker panels which fulfill the WHO minimum criteria and triage optimum and confirmatory minimum requirements, dependent on the control group(s) used for comparison.

Zak et al. reported GBP1 in a signature for disease risk (111), where GBP1, STAT1, and TAP1 were considered to be protective and associated with a good clinical outcome. Sweeney et al. re-ported a three-gene signature GBP5+DUSP3+KLF2 that can correctly identify ATB from healthy controls and LTBI at high risk of progression (120). In comparison to these panels, our GBP1+ IFIT3 and GBP1+IFIT3 +SAMD9L panels gave similar results for discrimination of ATB from controls (AUC - 0.95 and 0.94), and for discrimination of ATB from LTBI non-progressors (AUC - 0.91). Additionally, our GBP1+ IFITM3 panel could identify LTBI from both combined control groups with an AUC of 0.81. When just the CNTRLA control group only was used, the AUC increased to 0.96. The performance of our GBP1+ IFIT3 and GBP1+IFIT3 +SAMD9L panels for ATB also compare favorably with the Indian-lasso-24 signature published by Leong [(110) AUC 0.984] and the RISK6 signature published recently by Penn-Nicholson et al. (AUC 0.936). Our panels exhibited slightly reduced ROC curve values than the lasso-24 on the same data set, however they are smaller and more amenable to multiplex assay development.

One observation from the Leong study is the small number of ATB outlier patient samples which fall outside of the experimental error and appear as false negatives in the scatter plots. There appears to be a subgroup of patients which segregate with the control samples. This is consistent with our own observations in our study where a proportion of patients in the ATB groups test negative for most of the biomarkers assayed. It would be interesting to determine whether these represent a subgroup of patients displaying a different clinical profile to the others or symptom status as proposed previously by Blankley and co-workers (112), e.g., disease severity, defective immune response/developed anergy. This is worth further investigation and could perhaps be characterized using other analytical means, e.g. flow cytometry.

In summary, we have validated a number of TB-associated whole blood PBL immune gene markers in new cohorts of patients and controls using qPCR, of which seventeen were significant. Their utility in primary determination of ATB (both pulmonary and extra-pulmonary manifestations) and LTBI has been assessed using ROC curve analysis and evaluated against the WHO TPP requirements for a triage and confirmatory test. ATB disease could be detected with a high degree of accuracy and sensitivity, including EPTB with LTBI detected at a somewhat reduced level. We have shown that minimally small configurations of biomarkers show comparable performance in relation to other studies. They exhibit the requisite TPP requirements for further evaluation and development on our data set, however variable performance was observed with other previously published data sets. This may be due in part to technical variation with the variety of assay platforms used, the contribution of which may be underestimated in contemporary comparison studies. Our biomarker panels could be easily formulated into a simple multiplex qPCR assay format and used in diagnosis/screening surveillance for all TB presentations, however further validation is required. The assays in this study were conducted on the Roche lightCycler 480, but these could be adapted easily to run on any qPCR platform as part of a low cost, rapid testing/screening program. Further work is underway to develop these panels as clinically useful, utilitarian diagnostic tests.

## Study Limitations

A key limitation of the study is the choice of control groups. The study includes controls, latent TB and TB patients recruited in the UK, but only TB patients in India. The preferred control group for the Indian group samples would be region-matched disease-free individuals and those with other respiratory conditions/infections. The number of LTBI individuals progressing to active disease is also relatively small and this limits the power of the statistical analysis, as they could not be analyzed as a separate group with the analytical methods used. In addition, the Indian blood samples were banked in Tempus tubes while those in the UK were banked into PAXgene tubes, which may have had an impact on the consistency of RNA extraction and recovery. Limited demographic information was available on the patients included in the study. Future studies would be planned to address these issues and further empower the analyses.

## Data Availability Statement

The raw data supporting the conclusions of this article will be made available by the authors, without undue reservation.

## Ethics Statement

The studies involving human participants were reviewed and approved by JIPMER Institute Ethics committee, AIIMS Institute Ethics committee, the NHS Health Research Authority Research Ethics (NRES) Committee for London - Camden & Islington and PHE. The patients/participants provided their written informed consent to participate in this study.

## Author Contributions

PP, MA, and KE conducted the experimental work. IH, ML, TM, JS, RB, GS, SK, IA, SS, and NJ provided control and patient samples and clinical and scientific expertise to the project. PP, NJ, SV, and KK designed the study protocol and managed the study. PP, MA, HG, and KK conducted the data analysis, and HG, IH, ML, TM, KE, NJ, and KK wrote and edited the paper. All authors contributed to the article and approved the submitted version.

## Funding

This study was funded by the UK Department of Health and Social Care Grant in Aid and the Public Health England Pipeline fund. The views expressed in this publication are those of the authors and not necessarily those of PHE or the DH.

## Conflict of Interest

The authors declare that the research was conducted in the absence of any commercial or financial relationships that could be construed as a potential conflict of interest.

## Supplementary Material

Supplementary Table S1 **Table 1.1**. Details of Patient and Control Samples **Table 1.2** Summary of numbers of patients per group and affiliations **Table 1.2**. Summary of patients or controls recruited per collaborating site used in the study **Table 1.3**. Number of participants per PREDICT TB LTBI and CNTRLB study groups study groups classified by TST IGRA status and progression to active TB **Table 1.4** Gene entities validated in study using Roche Real-time Ready qPCR assays with assay configuration identifiers and ascribed biological function **Table 1.5** Summary of the differentially expressed gene entities between the control, latent and active TB disease groups in the study from ANOVA SNK analysis **Table 1.6** ROC/AUC values from pairwise comparisons for single biomarkers between control, latent and active TB disease groups **Table 1.7** ROC/AUC values from pairwise comparisons for single biomarkers between control and latent TB progressor and non-progressor groups.

Supplementary Table S2 Summary of genes from previously published studies overlapping with those analyzed in this study (highlighted in red text) and performance to WHO TPP requirements based on source or reviewed sensitivity and specificity values.

Supplementary Table S3 **Table 3.1**. p values of entities differentially regulated be-tween groups from ANOVA performed across all groups using BH FDR (corrected p value < 0.05) and the SNK *post hoc* test, plus direction of regulation. **Table 3.2**. Detailed comparison of entities differentially regulated between groups from the SNK *post hoc* test table, comparison and p values for significant entities only. **Table 3.3**. Fold change comparison for all 17 statistically significant biomarkers between the LTBI-NPR and LTBI-PR groups. **Table 3.4**. Individuals in the CNTRLA, CNTRLB, LTBI_NPR, and LTBI_PR groups ranked according to their cumulative average expression values for the 7plex signature, using cutoff points set at equal to and greater than the mean $\left(>\bar{X}\right)$ and $1(\bar{X}+SD)$ or $2(\bar{X}+2SD)$ standard deviations above the mean; Greater than $\bar{X}+2SD$ , between $\bar{X}+SD$ and $\left(\bar{X}+2SD\right)$ , between $\left(\bar{X}\right)$ and $\left(\bar{X}+SD\right)$  **Table 3.5**. ROC/AUC results, plus sensitivity, specificity, likelihood ratios and positive and negative predictive values for single significant biomarkers across all ATB and LTBI groups compared with controls **Table 3.6**. ROC/AUC results, plus sensitivity, specificity, likelihood ratios and positive and negative predictive values for key combinations of significant biomarkers across all ATB and LTBI groups compared with controls **Table 3.7**. Diagnostic accuracy of biomarker panels assessed using fixed sensitivities and/or specificities defined by the WHO technology product profiles for triage or confirmatory tests **Table 3.8**. Comparison of performance of biomarker panels **(A–F)** with regard to control and patient groups and the WHO technology product profiles for the minimum and optimal triage and confirmatory tests.

Supplementary Table S4 Performance of optimal biomarker panels on other previously published data sets.

Supplementary Information S1 **Figure 1.1** Distribution of the number of entities differentially regulated between groups from ANOVA, performed across all groups using BH FDR (corrected p value < 0.05) and using the SNK *post hoc* test. **Figure 1.2**. Line graph of CD52 normalized intensity values across all TB disease and control groups. **Figure 1.3**. Line graph of GBP1 normalized intensity values across all TB disease and control groups. **Figure 1.4**. Line graph of GBP2 normalized intensity values across all TB disease and control groups. **Figure 1.5**. Line graph of GBP5 normalized intensity values across all TB disease and control groups. **Figure 1.6**. Line graph of HLA-B normalized intensity values across all TB disease and control groups. **Figure 1.7**. Line graph of IFIT3 normalized intensity values across all TB disease and control groups. **Figure 1.8**. Line graph of IFITM3 normalized intensity values across all TB disease and control groups. **Figure 1.9**. Line graph of IRF1 normalized intensity values across all TB disease and control groups. **Figure 1.10**. Line graph of LOC400759 normalized intensity values across all TB dis-ease and control groups. **Figure 1.11**. Line graph of NCF1C normalized intensity values across all TB disease and control groups. **Figure 1.12**. Line graph of PF4V1 normalized intensity values across all TB disease and control groups. **Figure 1.13**. Line graph of S100A11 normalized intensity values across all TB disease and control groups. **Figure 1.14**. Line graph of SAMD9L normalized intensity values across all TB disease and control groups. **Figure 1.15**. Line graph of STAT1 normalized intensity values across all TB disease and control groups. **Figure 1.16**. Line graph of TAF10 normalized intensity values across all TB disease and control groups. **Figure 1.17**. Line graph of TAPBP normalized intensity values across all TB disease and control groups. **Figure 1.18**. Line graph of TRIM25 normalized intensity values across all TB disease and control groups.

Supplementary Information S2 **Figure 2.1**. Cluster analysis on group averaged data for all statistically significant entities from ANOVA (p < 0.05) **(A)** low TB incidence region UK control group (CNTRLA) **(B)** low TB incidence region UK control from the PREDICT TB study group (CNTRLB) **(C)** low TB incidence region UK Latent TB from the PREDICT TB study group (LTBI) **(D)** high TB incidence region extra-pulmonary TB group (IEPTB) **(E)** low TB incidence region UK pulmonary TB group (UKPTB) **(F)** high TB incidence region pulmonary TB group (IPTB). Entities which show expression differences between the LTBI_NPR and LTBI_PR groups are boxed in red, with the boxed region showing the highest expression compared to that in the relative other group.

Supplementary Information S3 **Figure 3.1** Heatmap of the 17-plex entities showing individual expression profiles across the disease and control groups. The top three ranked patient samples 2439, 1053 and 1864 from the cumulative scoring analysis using the 7-plex signature are highlighted with red arrows.

Supplementary Information S4 Description of the group association and biological function of the 17 statistically significant biomarkers from **Supplementary Information S4**.
