# Supplementary material for: Validation of Differentially Expressed Immune Biomarkers in Latent and Active Tuberculosis by Real-Time PCR
Source: Front Immunol. 2021 Mar 16;11:612564. doi: 10.3389/fimmu.2020.612564 (PMC8029985; doi:10.3389/fimmu.2020.612564)
Supplement: Supplementary Table S1 — Table 1.1. Details of Patient and Control Samples Table 1.2 Summary of numbers of patients per group and affiliations Table 1.2. Summary of patients or controls recruited per collaborating site used in the study Table 1.3. Number of participants per PREDICT TB LTBI and CNTRLB study groups study groups classified by TST IGRA status and progression to active TB Table 1.4 Gene entities validated in study using Roche Real-time Ready qPCR assays with assay configuration identifiers and ascribed biological function Table 1.5 Summary of the differentially expressed gene entities between the control, latent and active TB disease groups in the study from ANOVA SNK analysis Table 1.6 ROC/AUC values from pairwise comparisons for single biomarkers between control, latent and active TB disease groups Table 1.7 ROC/AUC values from pairwise comparisons for single biomarkers between control and latent TB progressor and non-progressor groups. [file DataSheet_1.zip › Supplementary Information S1.pptx]

## Slide 1
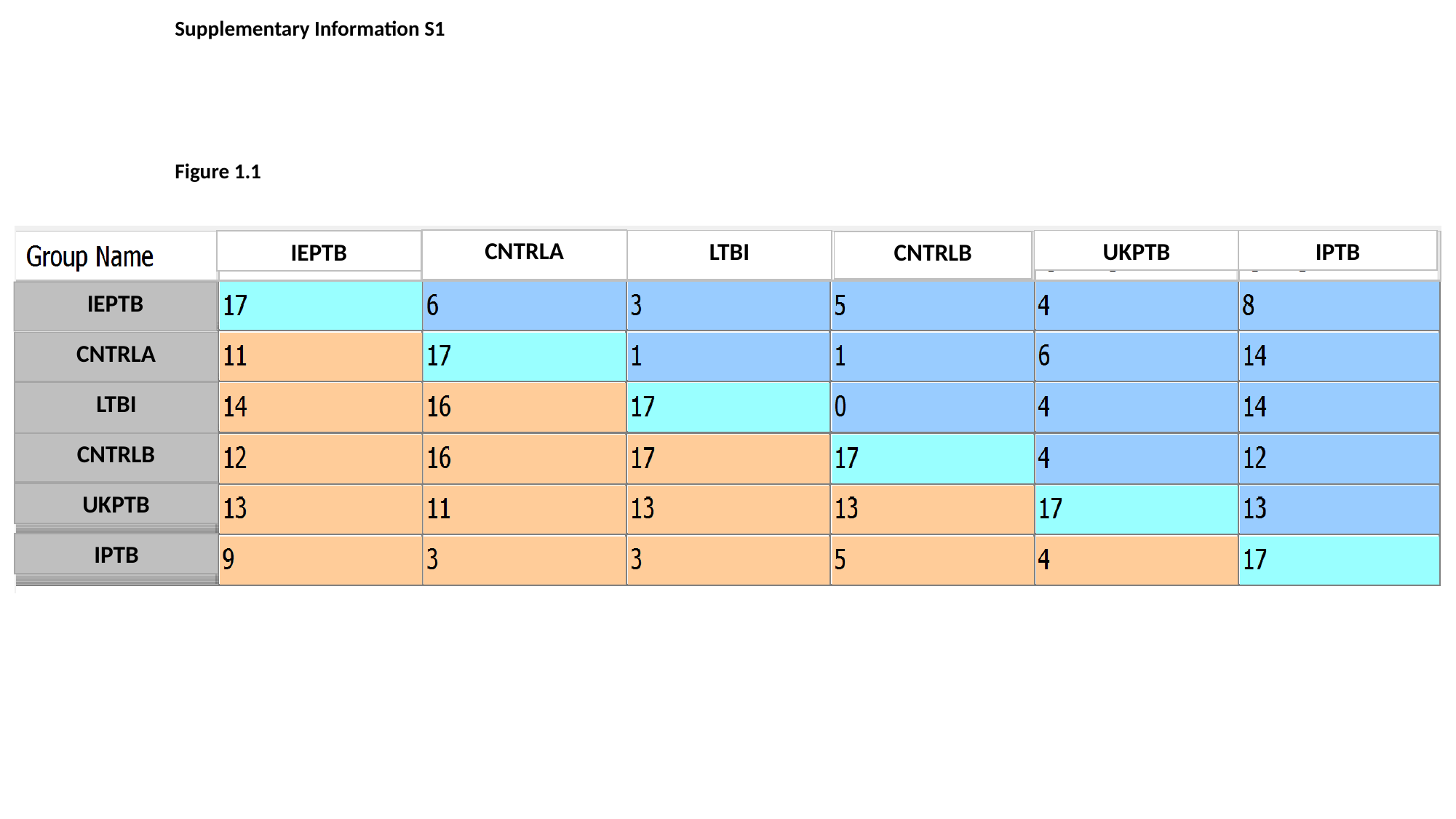

Supplementary Information S1
Figure 1.1
CNTRLA
LTBI
UKPTB
IPTB
IEPTB
CNTRLB
IEPTB
CNTRLA
LTBI
CNTRLB
UKPTB
IPTB

## Slide 2
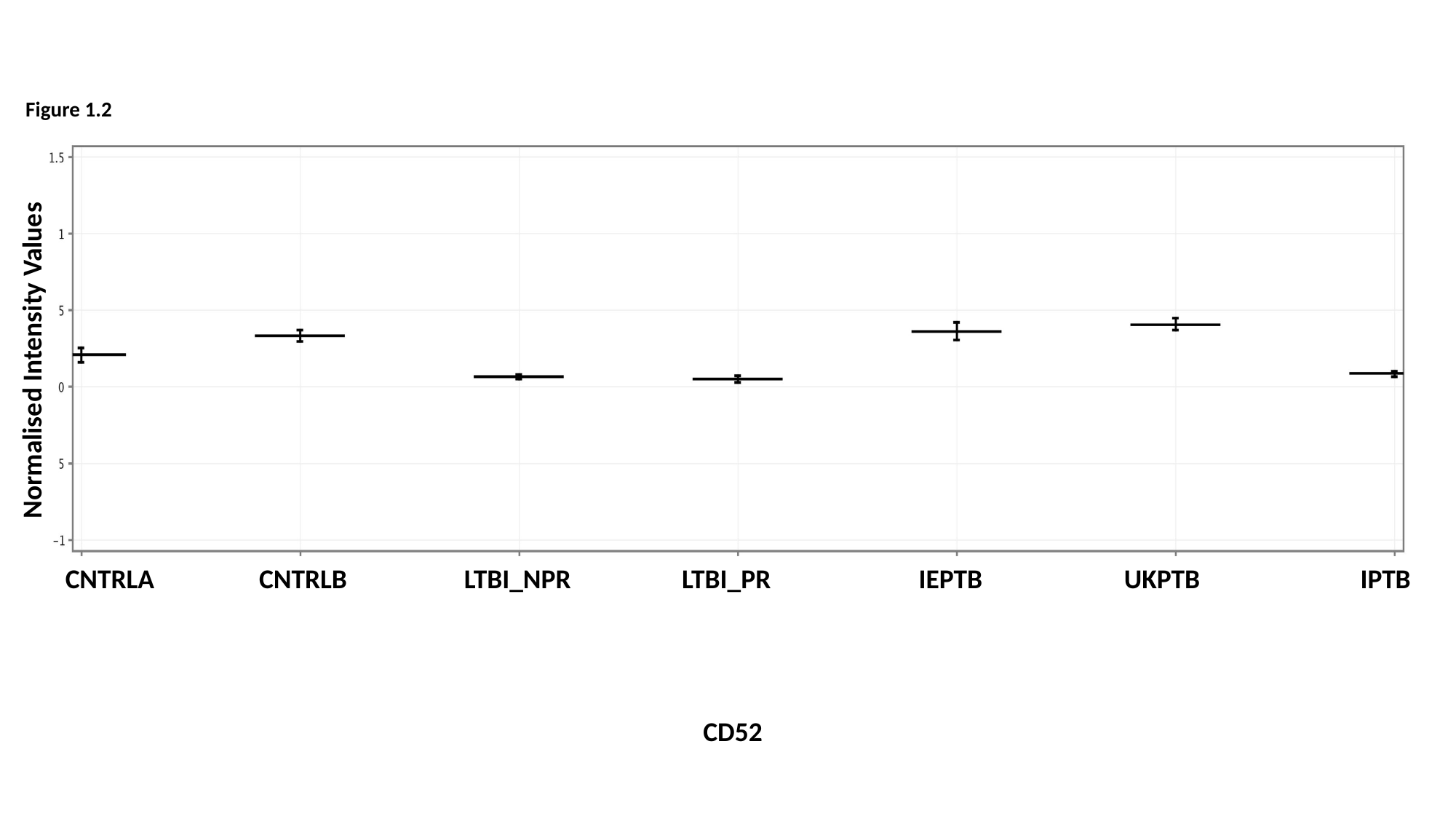

Figure 1.2
CNTRLA CNTRLB LTBI_NPR LTBI_PR IEPTB UKPTB IPTB
Normalised Intensity Values
CD52

## Slide 3
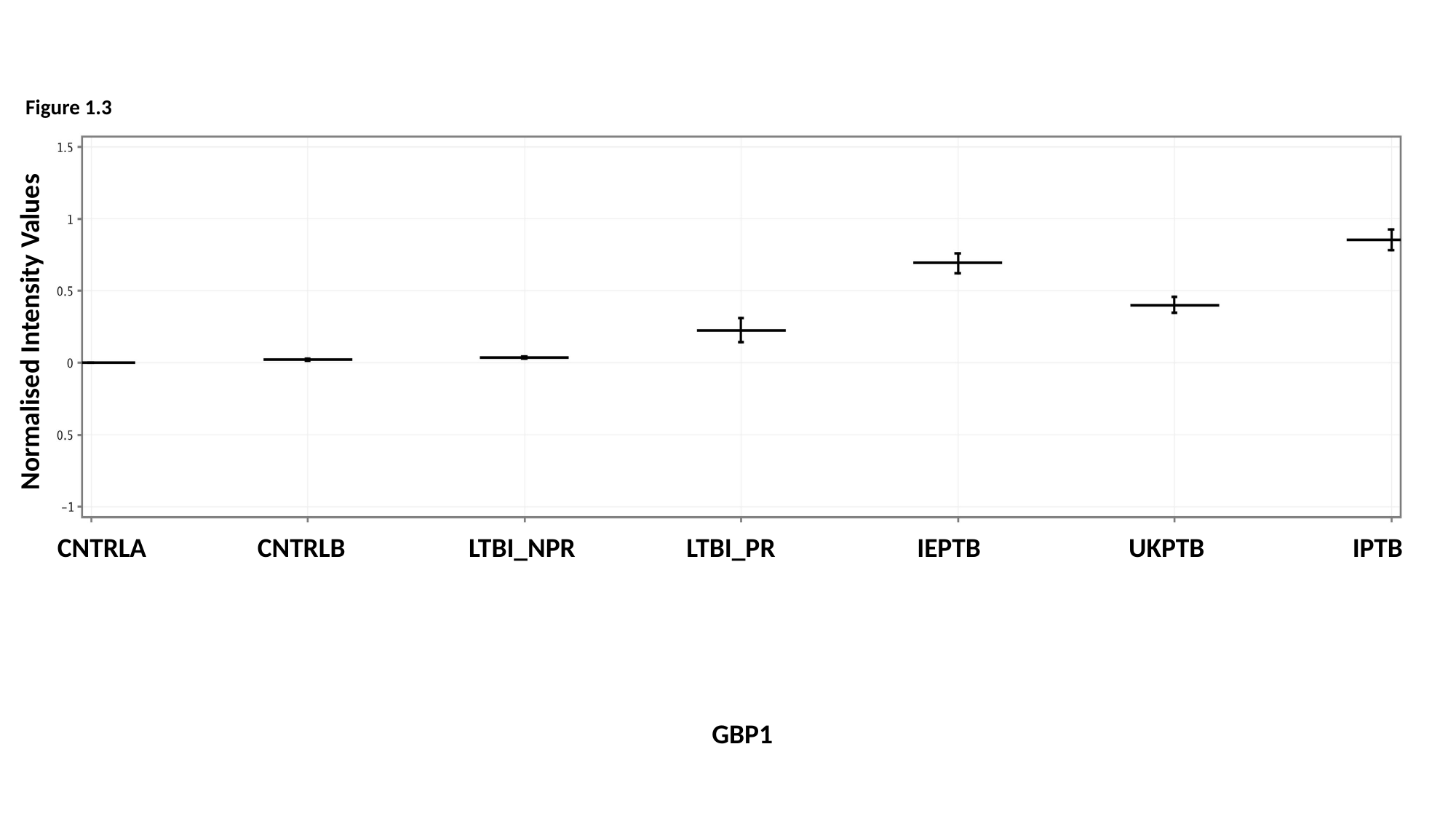

Figure 1.3
Normalised Intensity Values
CNTRLA CNTRLB LTBI_NPR LTBI_PR IEPTB UKPTB IPTB
GBP1

## Slide 4
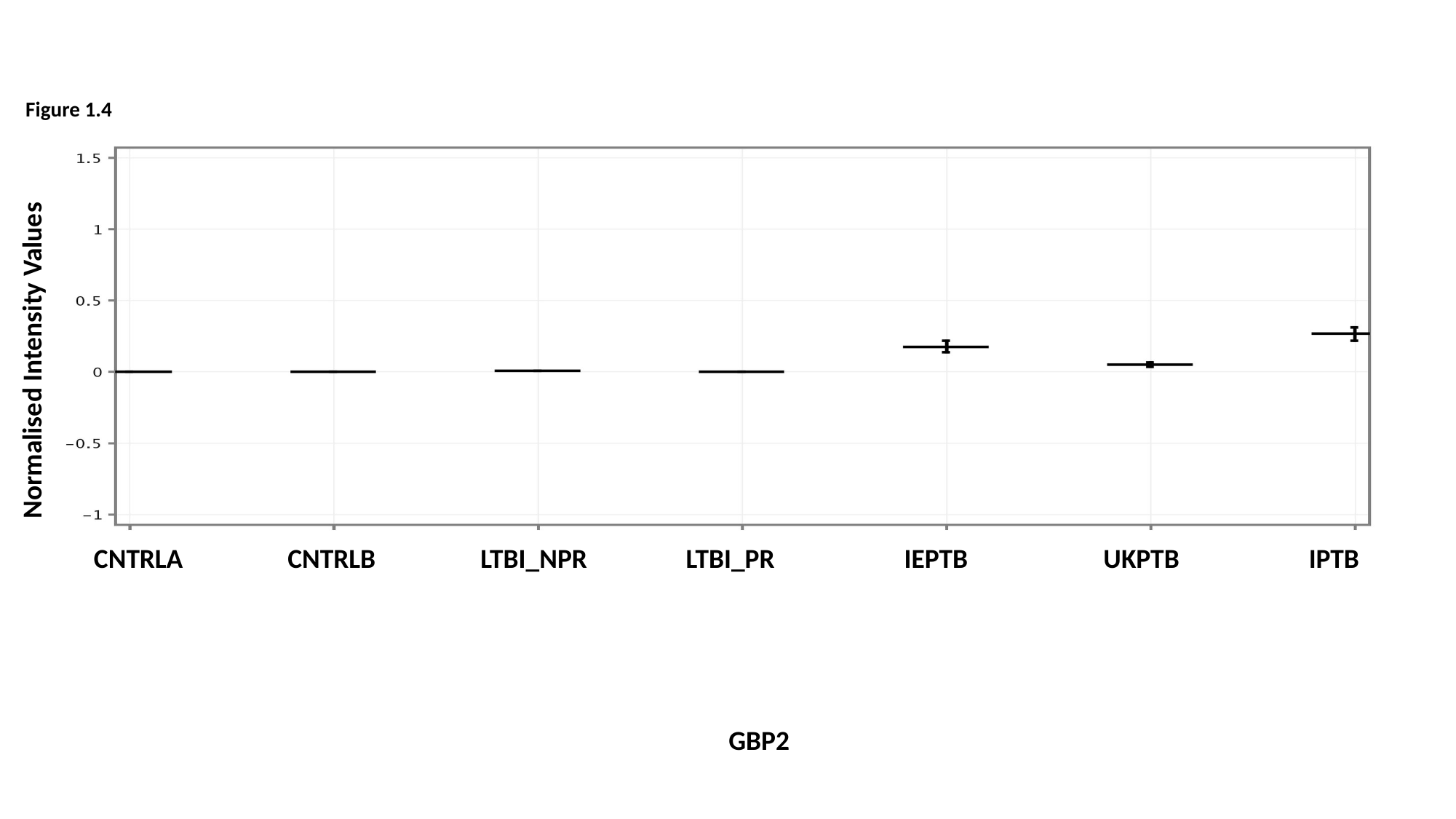

Figure 1.4
Normalised Intensity Values
CNTRLA CNTRLB LTBI_NPR LTBI_PR IEPTB UKPTB IPTB
GBP2

## Slide 5
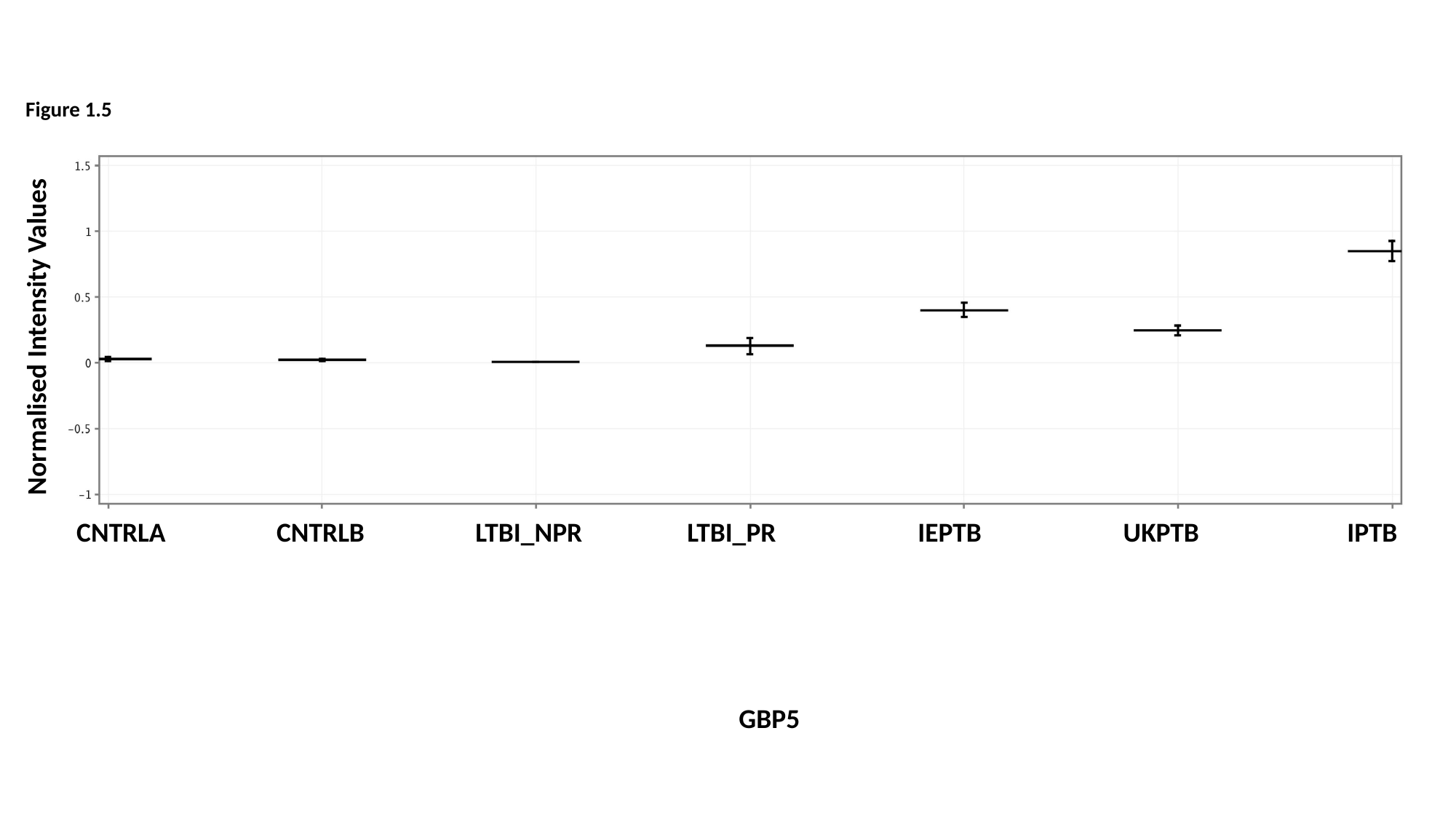

Figure 1.5
Normalised Intensity Values
 CNTRLA CNTRLB LTBI_NPR LTBI_PR IEPTB UKPTB IPTB
GBP5

## Slide 6
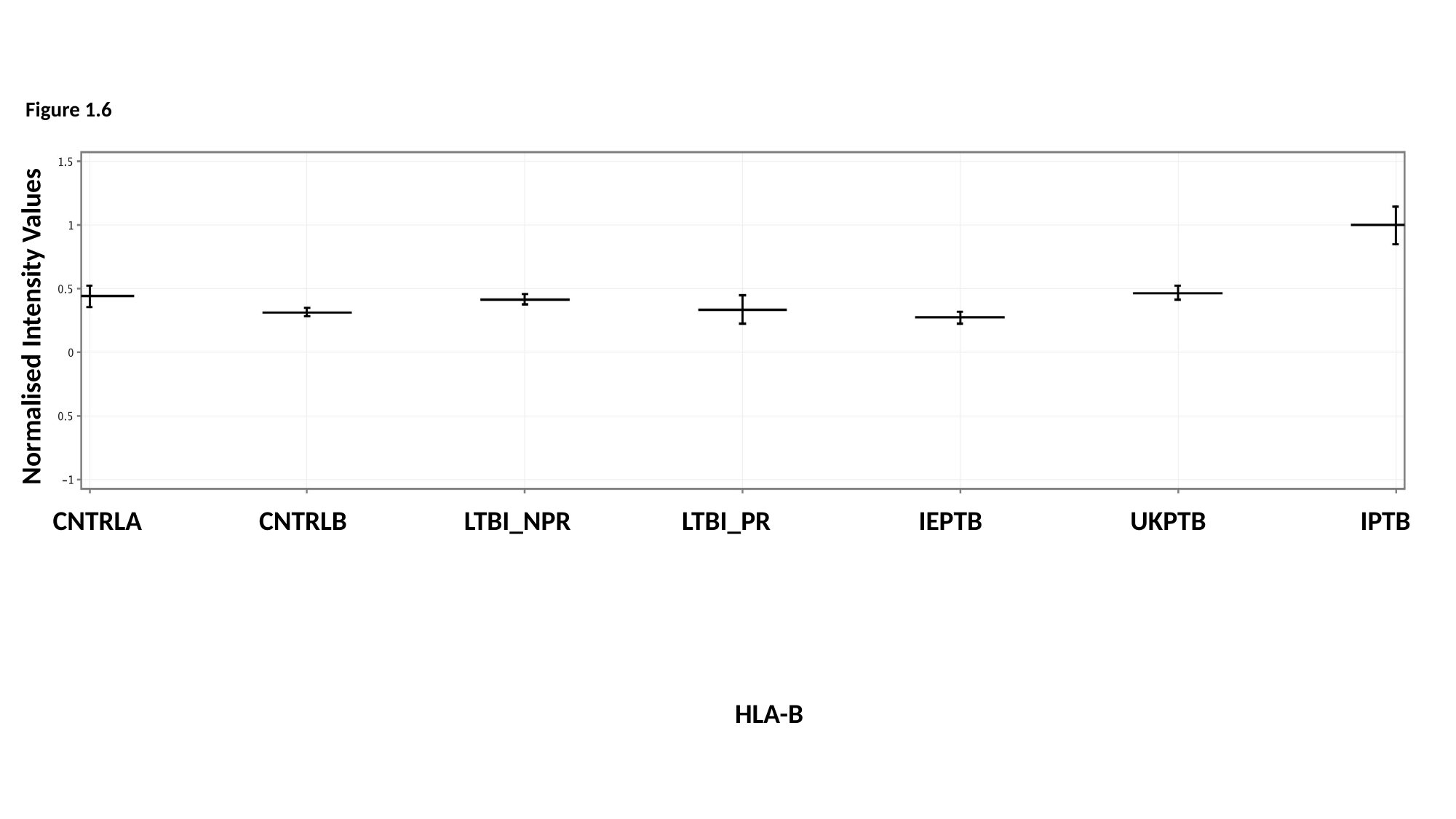

Figure 1.6
Normalised Intensity Values
CNTRLA CNTRLB LTBI_NPR LTBI_PR IEPTB UKPTB IPTB
HLA-B

## Slide 7
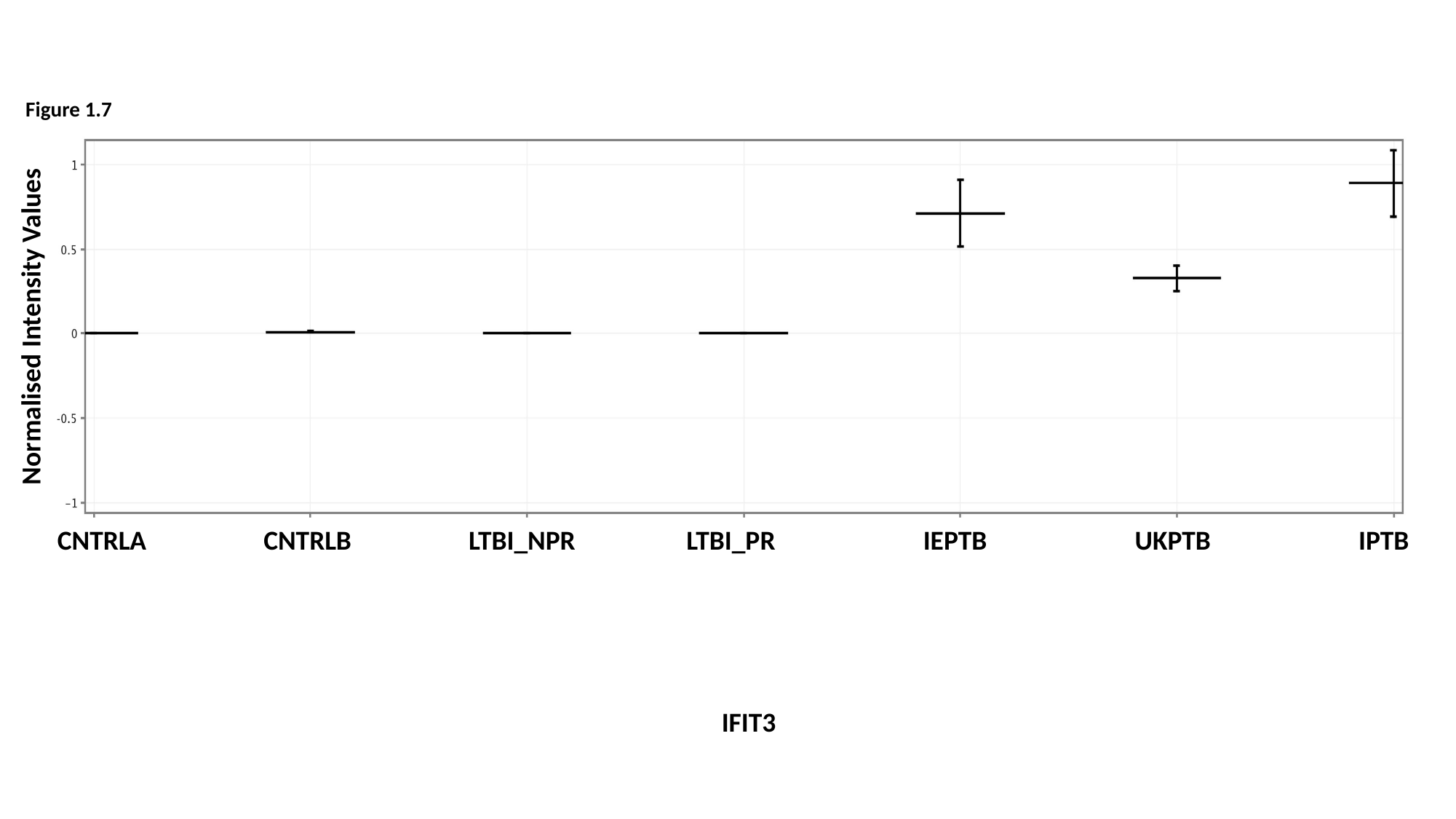

Figure 1.7
Normalised Intensity Values
CNTRLA CNTRLB LTBI_NPR LTBI_PR IEPTB UKPTB IPTB
IFIT3

## Slide 8
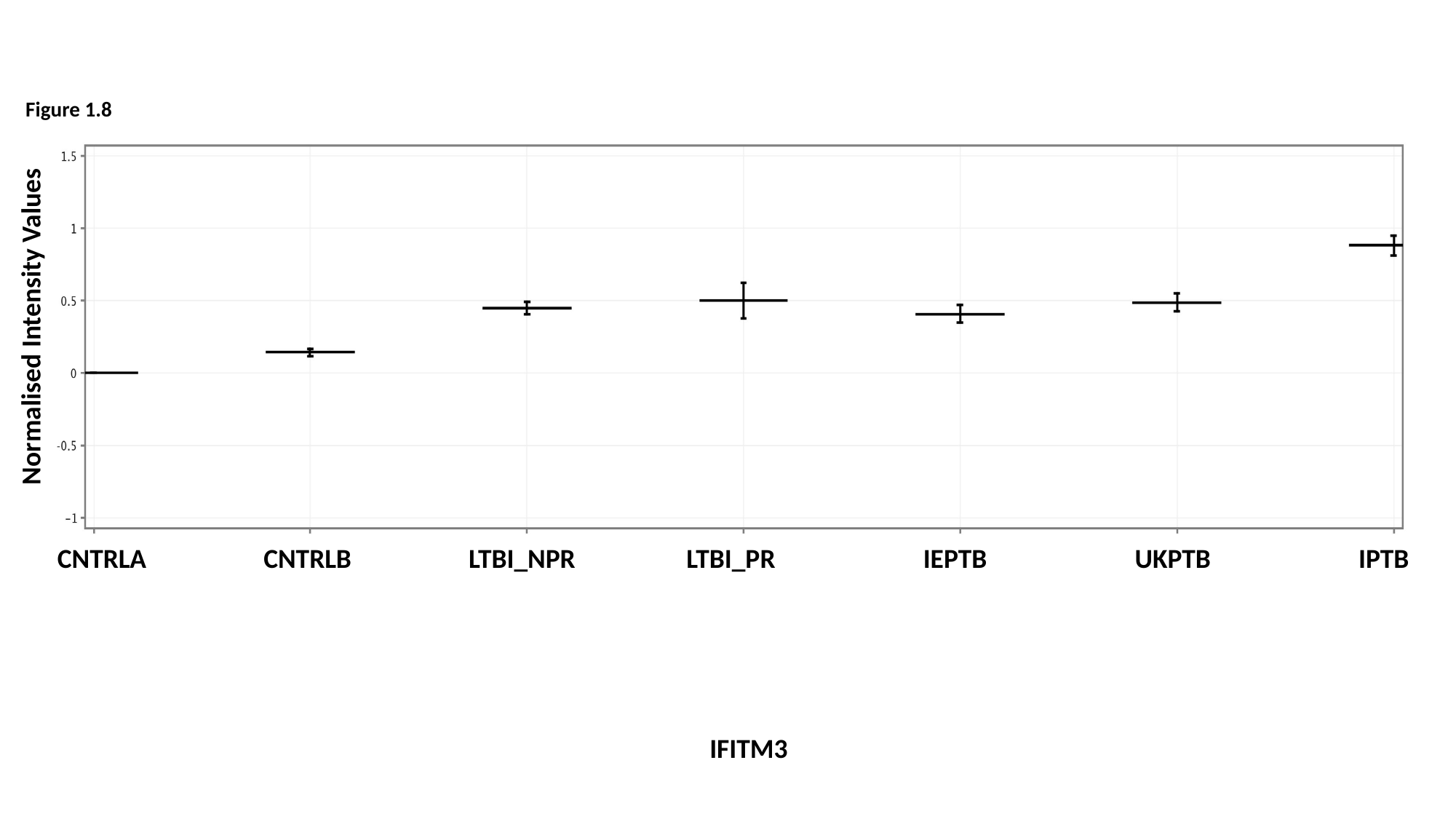

Figure 1.8
Normalised Intensity Values
CNTRLA CNTRLB LTBI_NPR LTBI_PR IEPTB UKPTB IPTB
IFITM3

## Slide 9
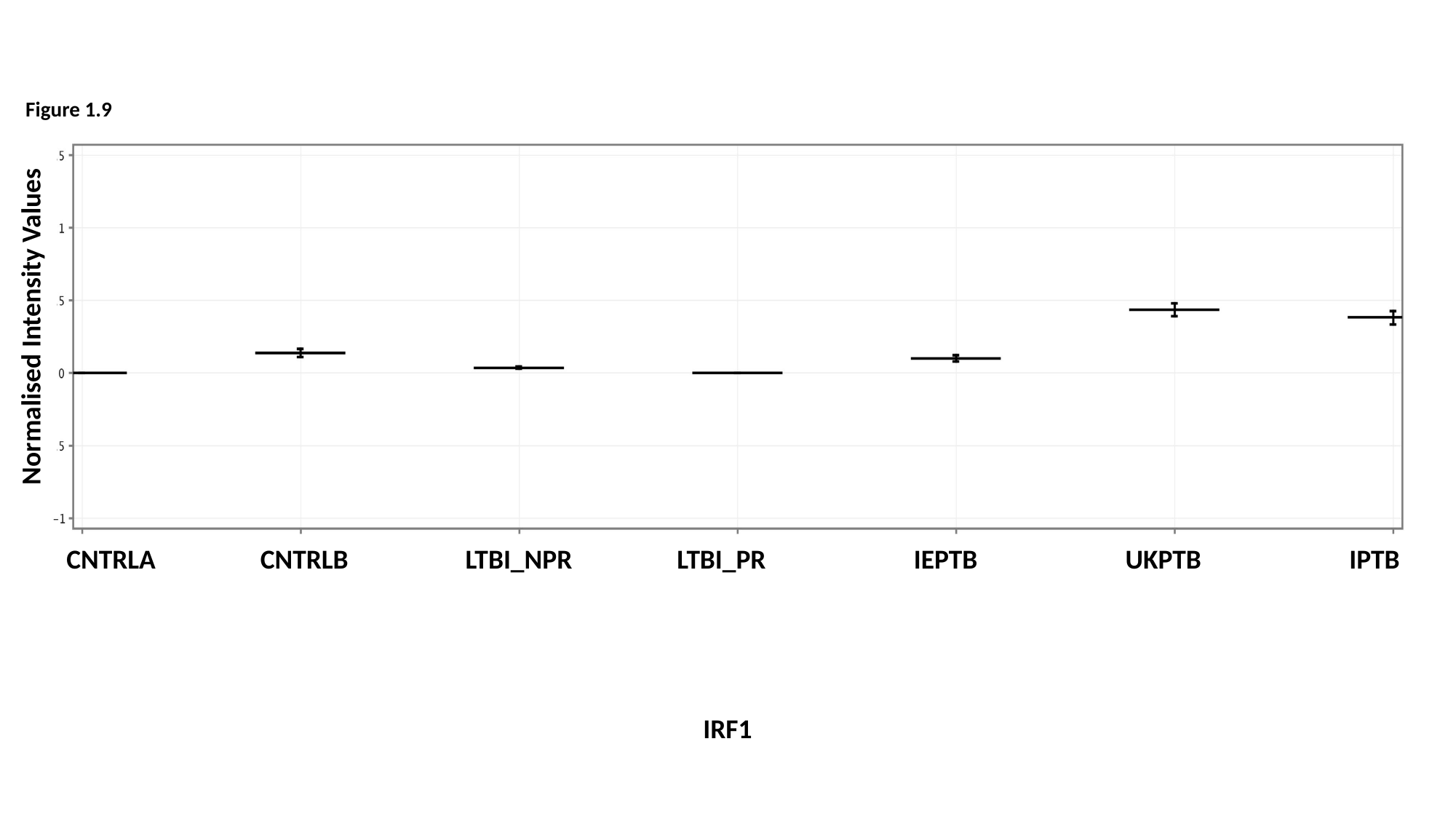

Figure 1.9
CNTRLA CNTRLB LTBI_NPR LTBI_PR IEPTB UKPTB IPTB
Normalised Intensity Values
IRF1

## Slide 10
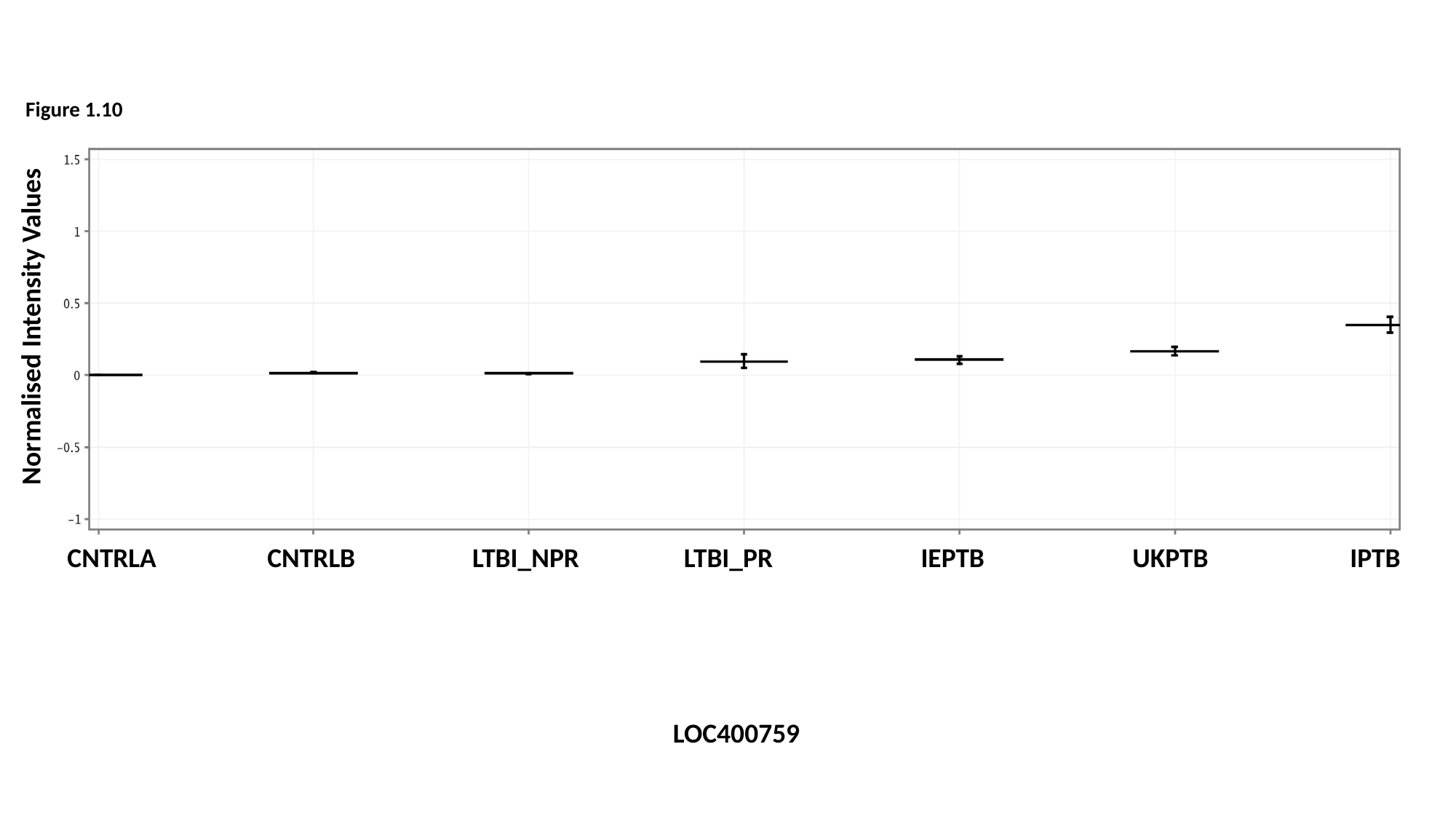

Figure 1.10
CNTRLA CNTRLB LTBI_NPR LTBI_PR IEPTB UKPTB IPTB
Normalised Intensity Values
LOC400759

## Slide 11
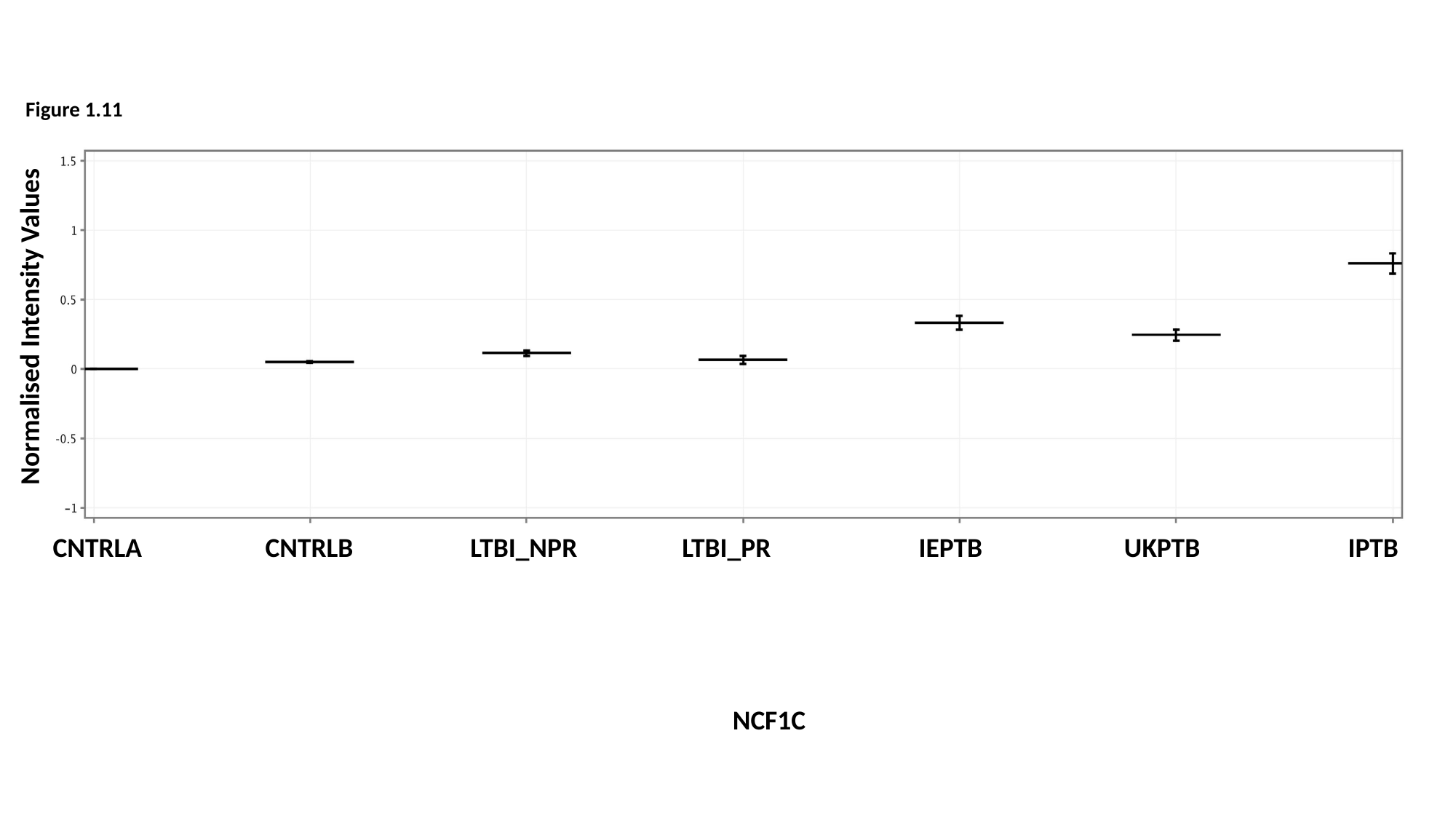

Figure 1.11
Normalised Intensity Values
CNTRLA CNTRLB LTBI_NPR LTBI_PR IEPTB UKPTB IPTB
NCF1C

## Slide 12
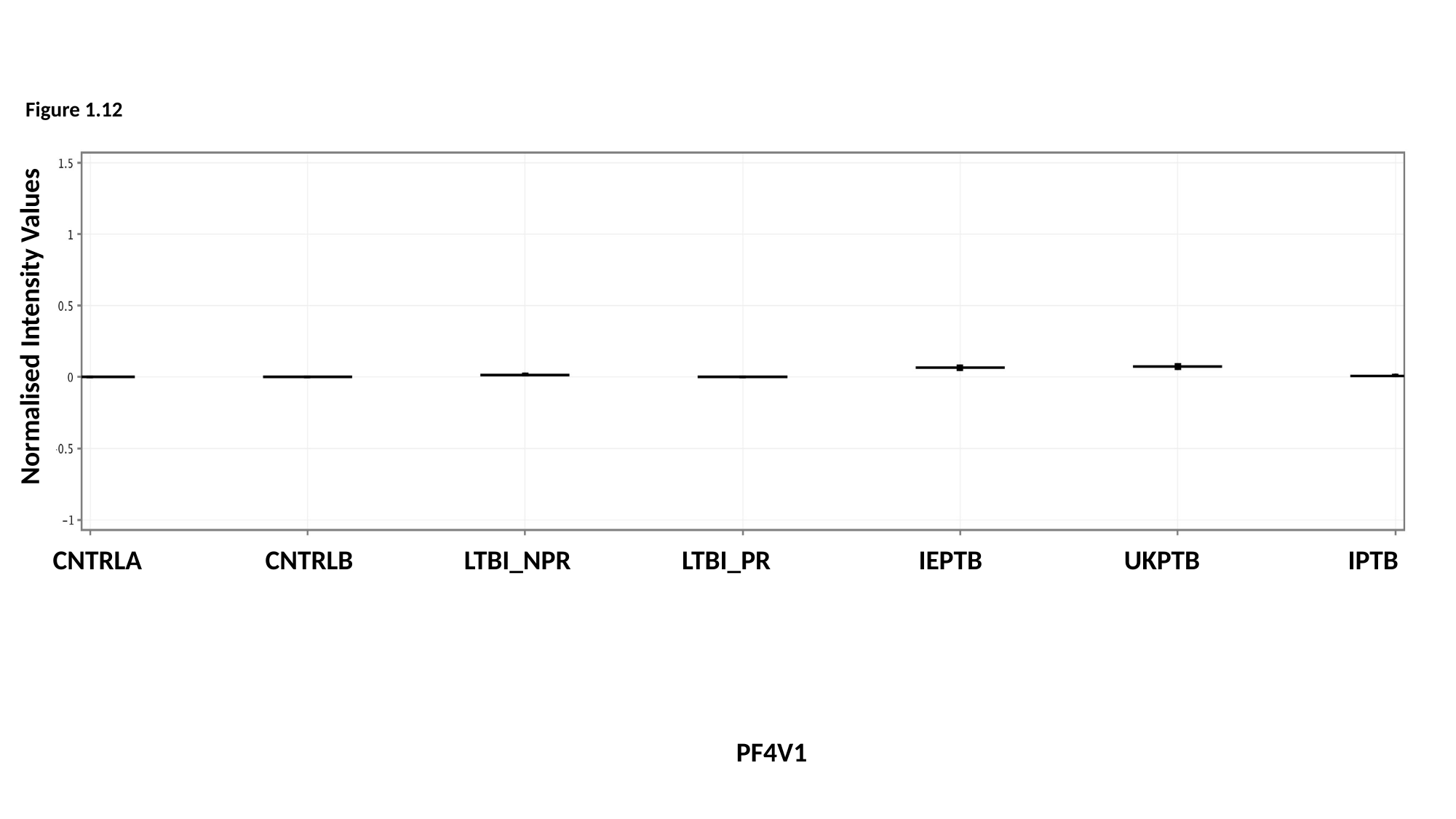

Figure 1.12
Normalised Intensity Values
CNTRLA CNTRLB LTBI_NPR LTBI_PR IEPTB UKPTB IPTB
PF4V1

## Slide 13
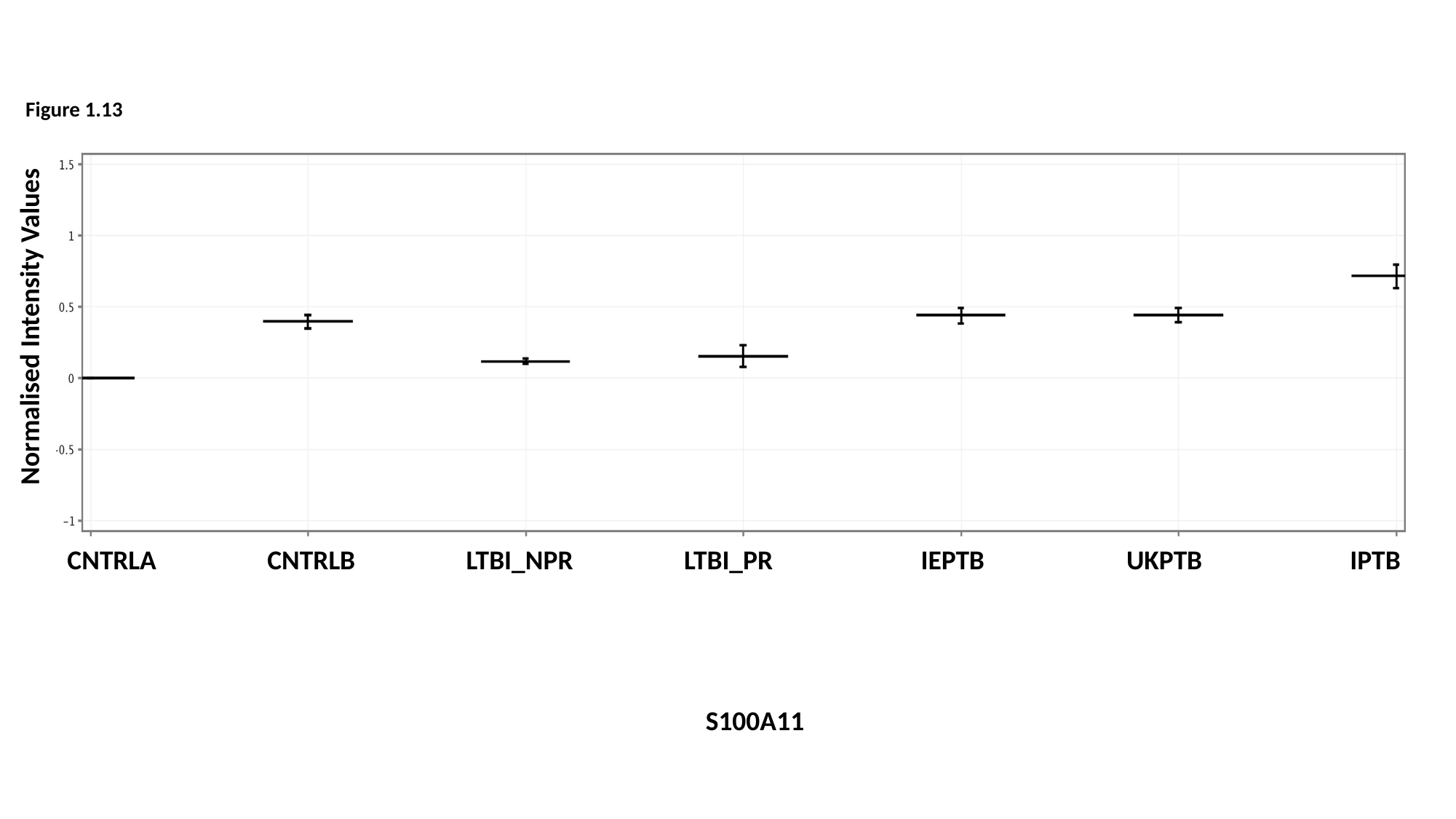

Figure 1.13
CNTRLA CNTRLB LTBI_NPR LTBI_PR IEPTB UKPTB IPTB
Normalised Intensity Values
S100A11

## Slide 14
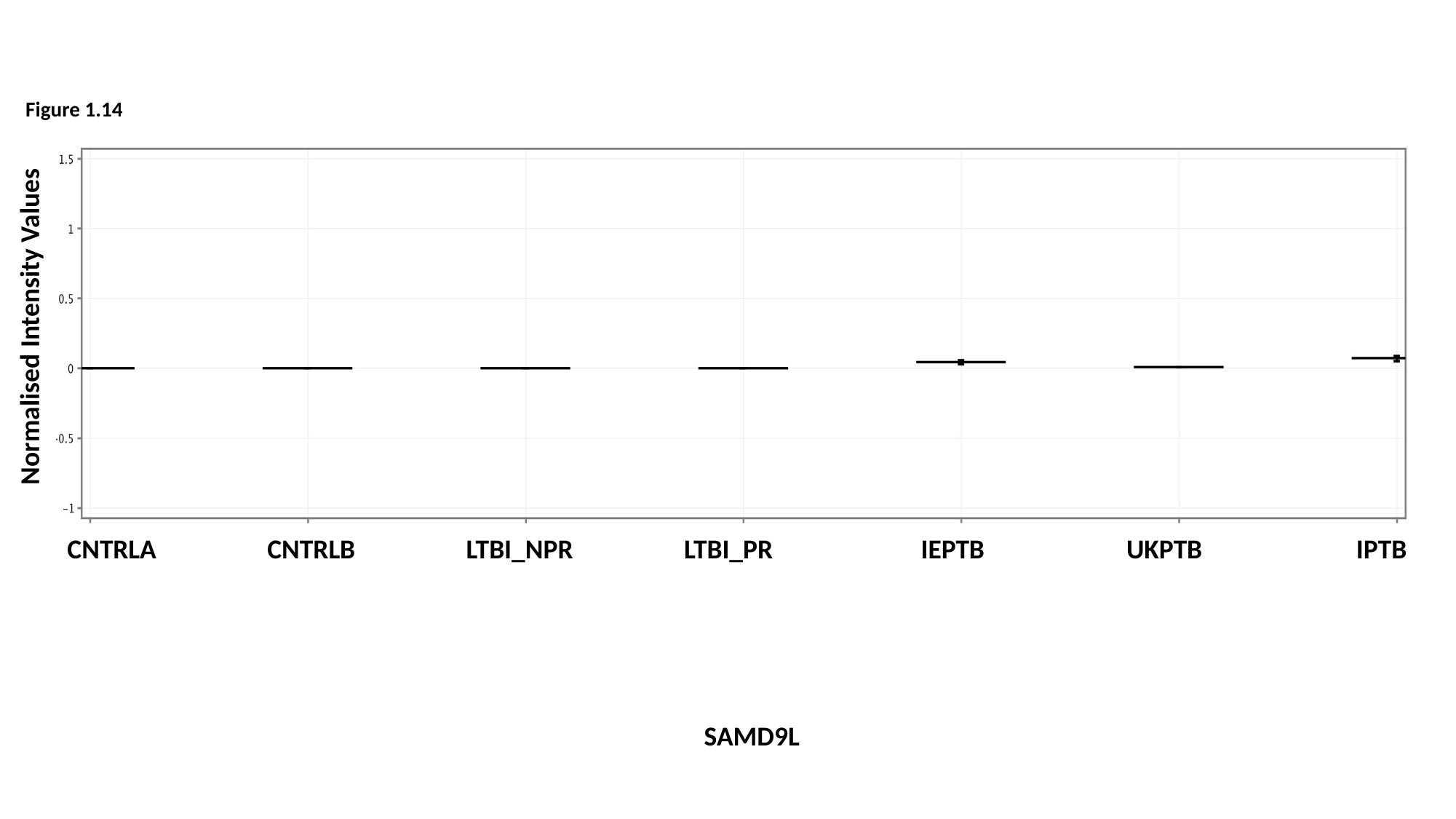

Figure 1.14
CNTRLA CNTRLB LTBI_NPR LTBI_PR IEPTB UKPTB IPTB
Normalised Intensity Values
SAMD9L

## Slide 15
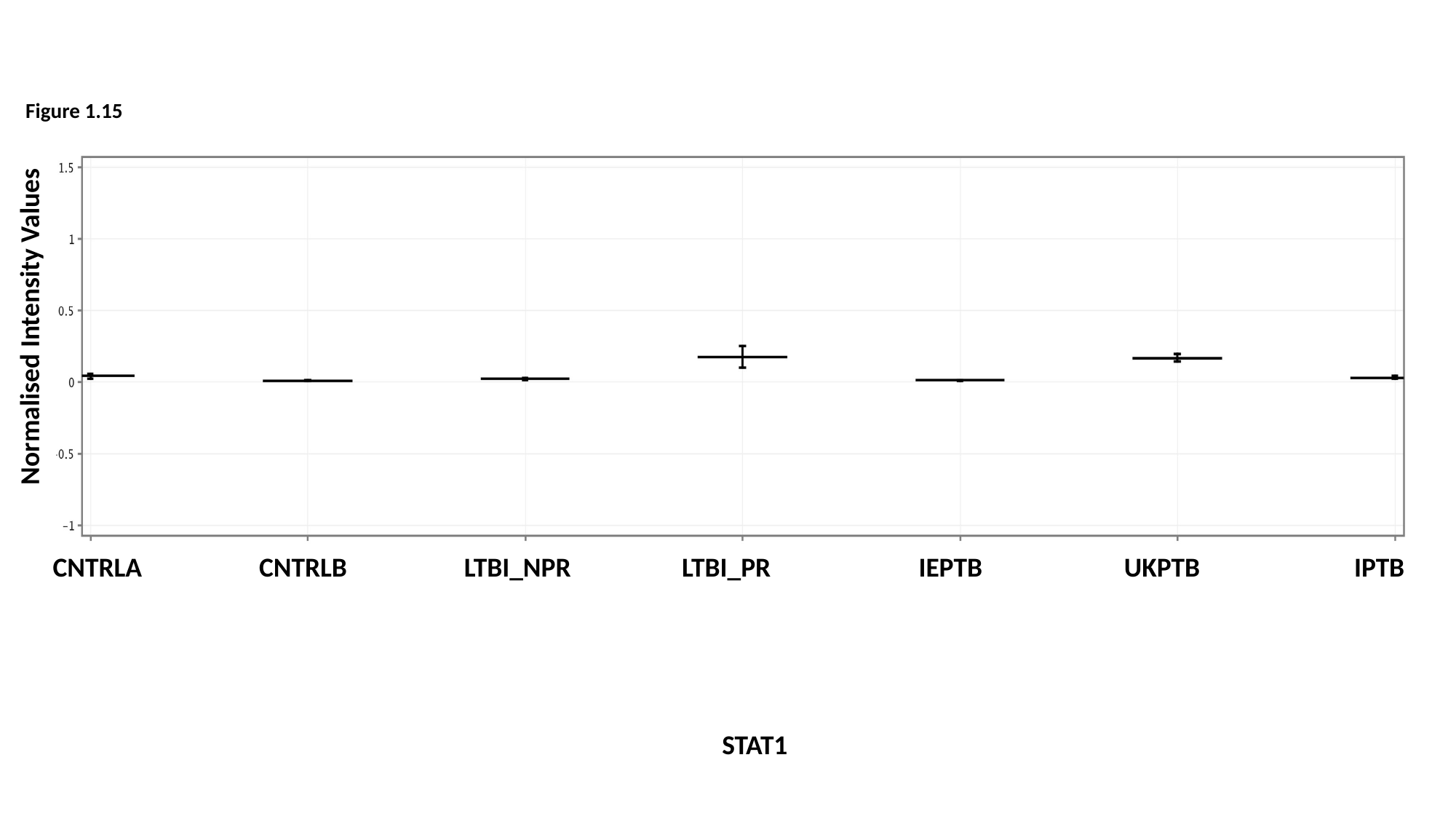

Figure 1.15
Normalised Intensity Values
CNTRLA CNTRLB LTBI_NPR LTBI_PR IEPTB UKPTB IPTB
STAT1

## Slide 16
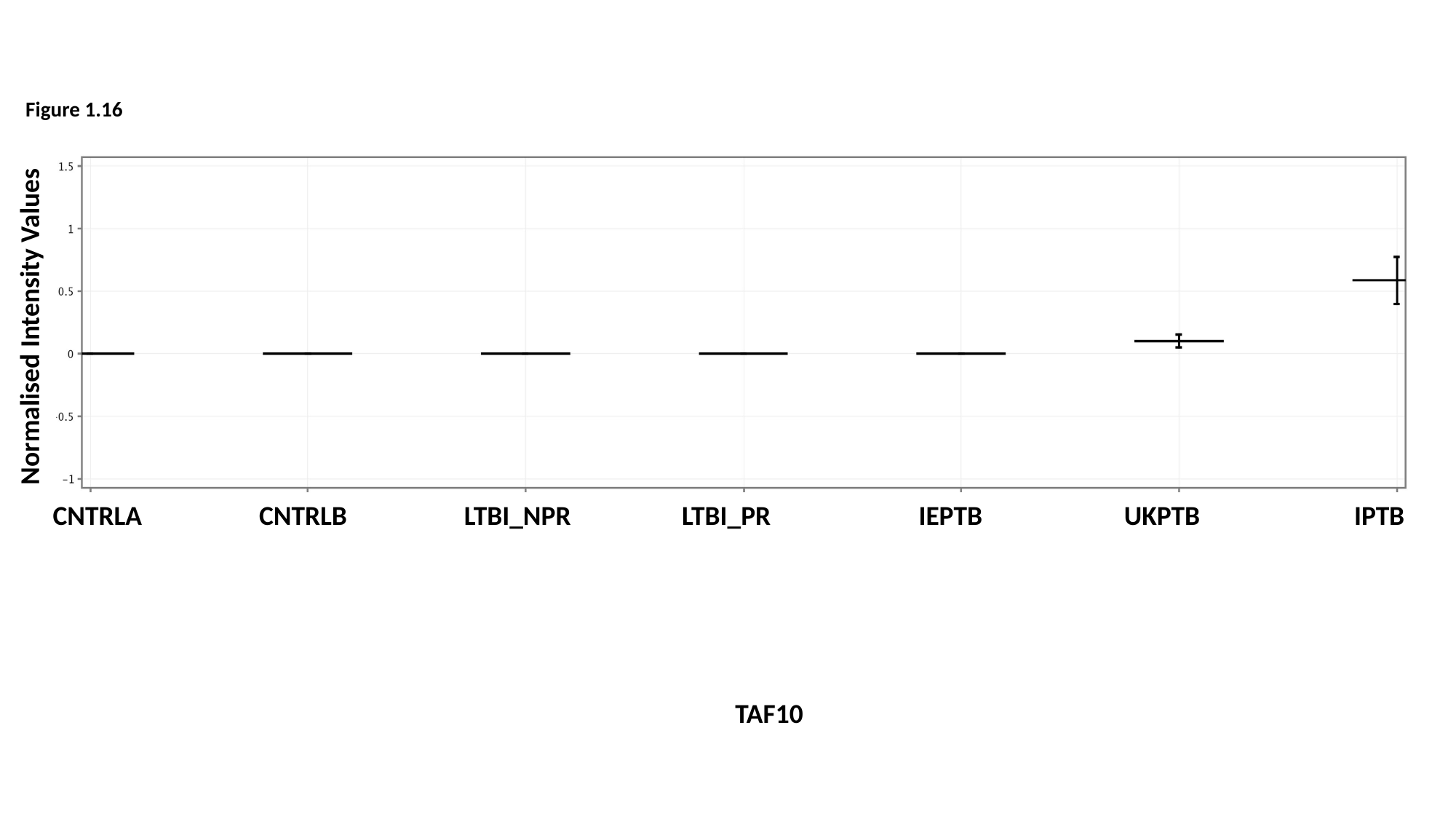

Figure 1.16
Normalised Intensity Values
CNTRLA CNTRLB LTBI_NPR LTBI_PR IEPTB UKPTB IPTB
TAF10

## Slide 17
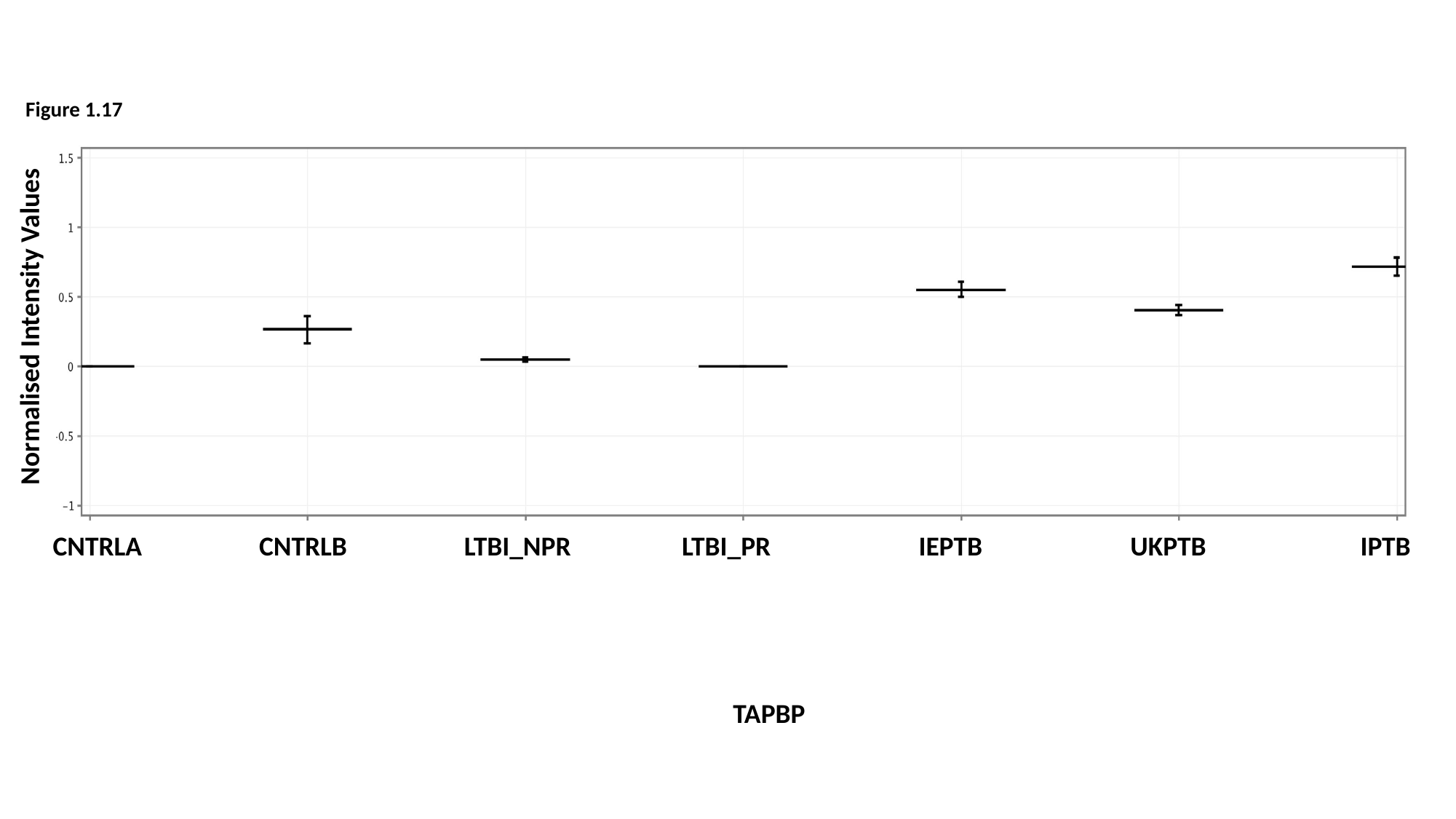

Figure 1.17
Normalised Intensity Values
CNTRLA CNTRLB LTBI_NPR LTBI_PR IEPTB UKPTB IPTB
TAPBP

## Slide 18
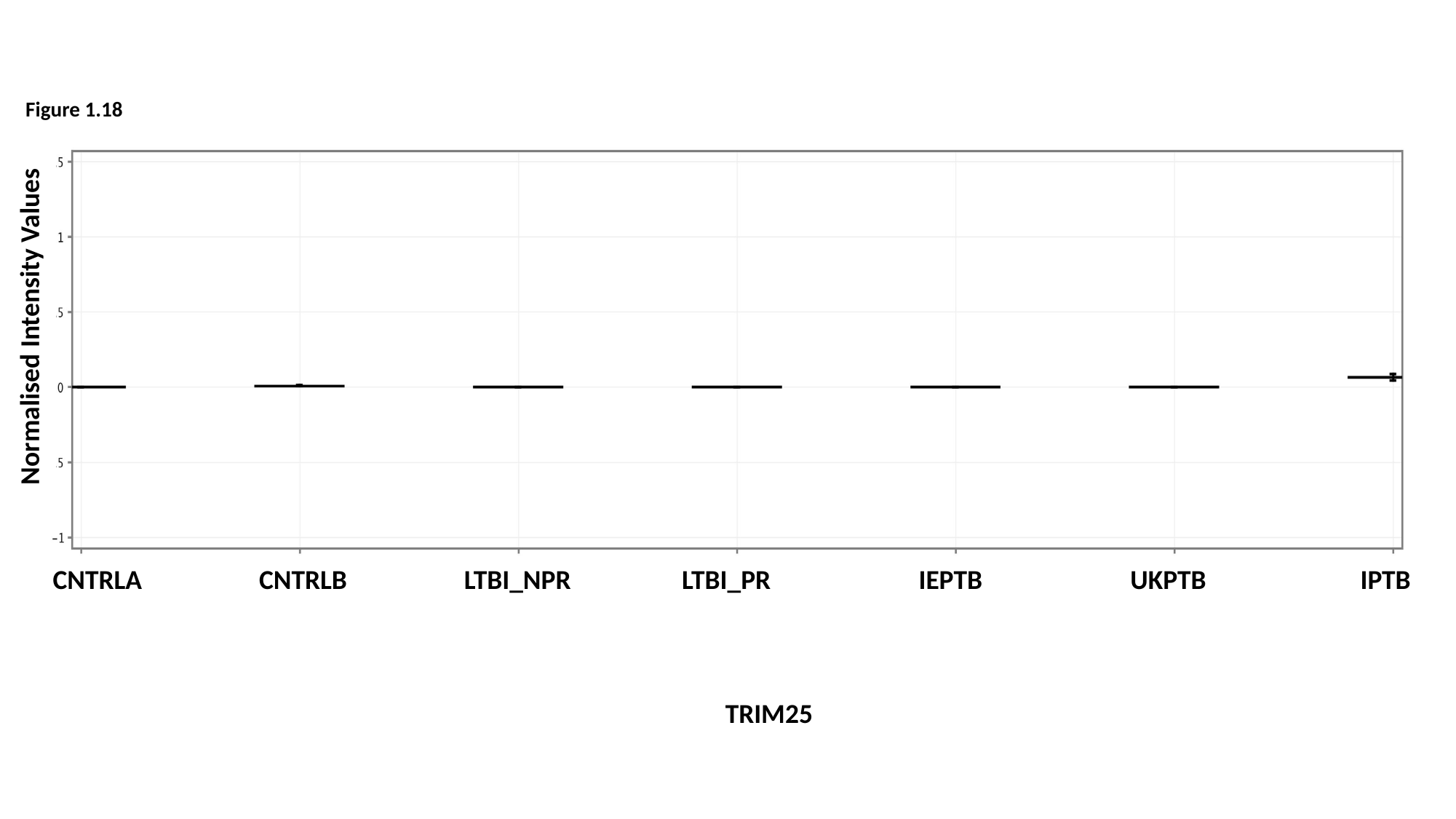

Figure 1.18
Normalised Intensity Values
CNTRLA CNTRLB LTBI_NPR LTBI_PR IEPTB UKPTB IPTB
TRIM25
