# Supplementary material for: Validation of Differentially Expressed Immune Biomarkers in Latent and Active Tuberculosis by Real-Time PCR
Source: Front Immunol. 2021 Mar 16;11:612564. doi: 10.3389/fimmu.2020.612564 (PMC8029985; doi:10.3389/fimmu.2020.612564)
Supplement: Supplementary Table S1 — Table 1.1. Details of Patient and Control Samples Table 1.2 Summary of numbers of patients per group and affiliations Table 1.2. Summary of patients or controls recruited per collaborating site used in the study Table 1.3. Number of participants per PREDICT TB LTBI and CNTRLB study groups study groups classified by TST IGRA status and progression to active TB Table 1.4 Gene entities validated in study using Roche Real-time Ready qPCR assays with assay configuration identifiers and ascribed biological function Table 1.5 Summary of the differentially expressed gene entities between the control, latent and active TB disease groups in the study from ANOVA SNK analysis Table 1.6 ROC/AUC values from pairwise comparisons for single biomarkers between control, latent and active TB disease groups Table 1.7 ROC/AUC values from pairwise comparisons for single biomarkers between control and latent TB progressor and non-progressor groups. [file DataSheet_1.zip › Supplementary Information S4.docx]

**Supplementary Information S4**

Cluster 1a contained biomarkers IFITM3 (Supplementary Information S4) and the class I presentation molecule HLA-B. Although HLA-B is expressed across all groups, it is particularly upregulated in the IPTB group. This may reflect increased antigen presenting cell activity, perhaps to cytotoxic CD8+ cells, which is a previously observed feature of TB. Increased APC activity may perhaps indicate more active or advanced disease in this group. IFITM3 also showed somewhat generalised expression across all TB ‘exposed’ or infected groups. It was modestly upregulated (above a normalised expression value cut-off of 1.0) in the CNTRLB group, compared with CNTRLA group and may reflect exposure to MTB in some individuals within this group. However, expression was also proportionally higher in the IPTB group. IFITM3 positivity was also increased in the LTBI_NPR group and LTBI_PR groups. These results may perhaps indicate IFITM3 is a somewhat generalised marker for TB exposure. The currently available commercial tests for latent tuberculosis infection, the tuberculin skin test and interferon gamma release assays (IGRAs), detect an immune memory response to MTB, rather than the continued presence of viable organisms (7, 9, 19,

23–25). They have low specificity for predicting disease progression, translating into low positive predictive value (PPV) for incident tuberculosis (89). This marker may show potential as identifying a sub-group of individuals for follow up in these preclinical disease groups (CNTRLB, LTBI_NPR and LTBI_PR), however in a background where the gold standard tests may be inadequate in correct allocation to these patient groups, more work is required.

IFITM3 expression was also further upregulated in the active disease groups IEPTB, UKPTB and particularly in the IPTB group. This entity is a component of the early innate cellular response. Its immune function has mainly been studied in the context of antiviral activity and it has been identified as a key component of the anti-TB immune response. Mutations in IFITM3 have been associated with increased risk of paediatric TB in Han Chinese populations. Its expression is constitutive in some cells however it is induced by viral or other infection and some of its isoforms can be further upregulated by Type I and II interferons. These temporal activities may be distinct and reflected in the expression profiles we see across the groups, with modest expression in exposed/sub-clinically infected individuals and further subsequent upregulation, perhaps due to further inducible expression, in individuals with active disease. These two potential phases of IFITM3 during TB exposure and infection require further detailed study, but this entity may be an early sentinel marker of infection and useful in diagnosis.

Cluster 1b (Supplementary information S4) contained S100A11 and TAPBP. S100A11 is a member of a family of proteins involved in the regulation of several cellular processes and may function in motility, invasion, tubulin polymerization and also inflammation. TAPBP is a transmembrane glycoprotein which mediates interaction between newly assembled major histocompatibility complex (MHC) class I molecules and the transporter associated with antigen processing (TAP). This is essential for optimal peptide loading on the MHC class I molecules and is therefore also a key component in the immune response. Its activities therefore synergise with the cluster 1a marker HLA-B, consistent with activity related to innate immune cell activation, antigen processing and presentation. They exhibited similar expression profiles across the disease groups, with relatively higher expression in the CNTRLB, IEPTB, UKPTB and IPTB groups. They were somewhat more highly expressed in the LTBI-NPR and LTBI-PR groups, compared with the CNTRLA group.

The entities in clusters 1c and 1d NCF1C, IFIT3, GBP1, GBP5 showed highly similar expression profiles to those in cluster 1b, except that there was less expression in the CNTRLB group. These entities appear more specific for the ATB groups. NCF1C is a neutrophil cytosolic factor 1C pseudogene, which may play a role in the regulation of neutrophil cytosolic factor 1 (NCF1 or p47phox) the 47 kDa cytosolic subunit of neutrophil NADPH oxidase. This produces superoxide anion and is also involved in intracellular killing of microorganisms. This may suggest increased involvement of neutrophils in ATB, which has been observed previously and may further suggest some attenuation of the respiratory killing burst in these cells as a consequence of NCF1C upregulation. IFIT3 is an interferon-regulated gene, part of a family of entities whose immune function has largely been elucidated from studies on viral infections and has also been identified previously as a component of the proinflammatory response to TB in macrophages. Its expression along with other IFITs has been postulated to be representative of a Type I interferon-inducible module in the TB disease process. Other entities in this cluster have been postulated to be part of an interferon-inducible signature includes the GBPs i.e. GBP1 and GBP5. GBP1 appeared significant as a marker of infection across all the confirmed infected groups i.e. IEPTB, UKPTB and IPTB. This module also includes IRF1 (cluster 2d), TAP1 (as discussed above), STAT1, GBP2 and potentially LOC400759 (also called GBP1P1 (cluster 2e)), which lies downstream of GBP1 in the same transcriptional region on chromosome 1 at map location 1p22.2, in a cluster which also contains GBPs 2 to 7.

IFIT3 and GBP2 expression was confined to the extra-pulmonary and pulmonary disease groups. IRF1 and showed increased expression in the pulmonary TB groups, along with PF4V1. LOC400759 (GBP1P1), GBP1 and GBP5 showed increased expression in all TB disease and latent TB progressor (LTBI_PR) groups. STAT1 showed the greatest expression in the latent TB progressors (LTBI_PR) and UKPTB groups. These observations taken together are indicative of both Type I and II interferon responses and is consistent with observations that these classes of entities are overabundantly expressed in ATB and also LTBI outliers (109). Overall, there was a marked increase in these interferon-regulated genes in the ATB groups, indicating increased adaptive immune cell activity. Their relatively higher expression would perhaps suggest a transition to a more adaptive, interferon-regulated response as progression from LTBI to ATB occurs, from a more early ‘innate’ IFITM3-associated response.

The role of Type I and II interferons and regulation of interferon-regulated gene entities in TB has been documented extensively elsewhere (101, 104) and have been postulated to be a double-edged sword as it leads to immune responses which ultimately may not be productive. Singhania et al suggested that association with type I or type II interferon-inducible signatures may reflect responses in different cell types (147), the type I response indicative of myeloid cells i.e. macrophage, dendritic cell and granulocyte lineages and the type II response with αβ T cells, dendritic cells, and innate lymphoid cells.

CD52 which is a T-cell marker (also called CAMPATH-1 (cluster 2c)), appeared down-regulated in the LTBI_NPR, the LTBI_PR and the IEPTB groups compared with the other disease and control groups, This implies a reduction in T-cell numbers and/or dysregulation of this gene in T cells in the LTBI and IPTB groups and conversely an increase in T-cell numbers or upregulation of this gene in the UKPTB and IEPTB groups. The potential associated differences in T-cell numbers or relative gene expression between the various TB disease sub-types, also suggests differences in T-cell-related adaptive immunity. This has also been documented extensively elsewhere and could be involved with T-cell exhaustion in active disease, although other mechanisms such as exit of cells from the periphery or cell death cannot be excluded as alternate mechanisms for the observed results.

PF4V1, SAMD9L and TRIM25 showed modest upregulation in the ATB groups, with some differential regulation across these groups. PF4V1 is a factor highly similar to platelet factor 4, both produced by platelets. This may suggest increased involvement and/or activation of platelets in ATB. SAMD9L is a cytoplasmic protein that acts as a tumour suppressor but also plays a key role in cell proliferation. It has also been shown to be involved in the innate immune response to viral infection and expressed by peripheral blood leukocytes in other infectious conditions like sepsis. It is an endosome fusion facilitator and may therefore perhaps be involved in intracellular killing of TB bacilli in myeloid cells. TRIM25 is also a Type I interferon associated gene and is involved in anti-viral responses. It is the target for influenza A non-structural protein 1, which interferes with TRIM25 activity to suppress Type I IFN production via inactivation of the RIG-I-mediated viral RNA response pathway and prevent an efficient host immune response. It is therefore an integral component of the anti-viral response and vis a vis, the intracellular bacterial immune response, as there are considerable overlaps and of which TRIM25 is a key regulator. TAF10 is a component of the RNA polymerase II transcription complex, it’s role in disease pathology in this study is unclear, but it may be part of a generalised increase in cell transcription/metabolic functions as part of the ongoing inflammatory process.
