# Supplementary material for: Validation of Differentially Expressed Immune Biomarkers in Latent and Active Tuberculosis by Real-Time PCR
Source: Front Immunol. 2021 Mar 16;11:612564. doi: 10.3389/fimmu.2020.612564 (PMC8029985; doi:10.3389/fimmu.2020.612564)
Supplement: Supplementary Table S1 — Table 1.1. Details of Patient and Control Samples Table 1.2 Summary of numbers of patients per group and affiliations Table 1.2. Summary of patients or controls recruited per collaborating site used in the study Table 1.3. Number of participants per PREDICT TB LTBI and CNTRLB study groups study groups classified by TST IGRA status and progression to active TB Table 1.4 Gene entities validated in study using Roche Real-time Ready qPCR assays with assay configuration identifiers and ascribed biological function Table 1.5 Summary of the differentially expressed gene entities between the control, latent and active TB disease groups in the study from ANOVA SNK analysis Table 1.6 ROC/AUC values from pairwise comparisons for single biomarkers between control, latent and active TB disease groups Table 1.7 ROC/AUC values from pairwise comparisons for single biomarkers between control and latent TB progressor and non-progressor groups. [file DataSheet_1.zip › Supplementary Table S1.docx]

**Table S1.1 Details of Patient and Control Samples**

| **Patient Sample**  **ID** | **Disease**  **Type** | **Disease Status** | **Quantiferon** | **TSPOT** | **TST skin test** | **Recruiting Site** |
| --- | --- | --- | --- | --- | --- | --- |
| 160 | CNTRLA | NA | ND | ND | ND | PHE, Porton UK |
| 161 | CNTRLA | NA | ND | ND | ND | PHE, Porton UK |
| 162 | CNTRLA | NA | ND | ND | ND | PHE, Porton UK |
| 163 | CNTRLA | NA | ND | ND | ND | PHE, Porton UK |
| 170 | CNTRLA | NA | ND | ND | ND | PHE, Porton UK |
| 171 | CNTRLA | NA | ND | ND | ND | PHE, Porton UK |
| 172 | CNTRLA | NA | ND | ND | ND | PHE, Porton UK |
| 173 | CNTRLA | NA | ND | ND | ND | PHE, Porton UK |
| 174 | CNTRLA | NA | ND | ND | ND | PHE, Porton UK |
| 176 | CNTRLA | NA | ND | ND | ND | PHE, Porton UK |
| 177 | CNTRLA | NA | ND | ND | ND | PHE, Porton UK |
| 178 | CNTRLA | NA | ND | ND | ND | PHE, Porton UK |
| 179 | CNTRLA | NA | ND | ND | ND | PHE, Porton UK |
| 224 | CNTRLA | NA | ND | ND | ND | PHE, Porton UK |
| 225 | CNTRLA | NA | ND | ND | ND | PHE, Porton UK |
| 226 | CNTRLA | NA | ND | ND | ND | PHE, Porton UK |
| 231 | CNTRLA | NA | ND | ND | ND | PHE, Porton UK |
| 232 | CNTRLA | NA | ND | ND | ND | PHE, Porton UK |
| 233 | CNTRLA | NA | ND | ND | ND | PHE, Porton UK |
| 234 | CNTRLA | NA | ND | ND | ND | PHE, Porton UK |
| 80 | CNTRLB | NPR | Negative | Negative | Negative | PHE, NMRS-South UK |
| 227 | CNTRLB | NPR | Negative | Negative | Negative | PHE, NMRS-South UK |
| 228 | CNTRLB | NPR | Negative | Negative | Negative | PHE, NMRS-South UK |
| 229 | CNTRLB | NPR | Negative | Negative | Negative | PHE, NMRS-South UK |
| 230 | CNTRLB | NPR | Negative | Negative | Negative | PHE, NMRS-South UK |
| 477 | CNTRLB | NPR | Negative | Negative | Negative | PHE, NMRS-South UK |
| 547 | CNTRLB | NPR | Negative | Negative | Negative | PHE, NMRS-South UK |
| 622 | CNTRLB | NPR | Negative | Negative | Negative | PHE, NMRS-South UK |
| 982 | CNTRLB | NPR | Negative | Negative | Negative | PHE, NMRS-South UK |
| 1053 | CNTRLB | NPR | Negative | Negative | Negative | PHE, NMRS-South UK |
| 1067 | CNTRLB | NPR | Negative | Negative | Negative | PHE, NMRS-South UK |
| 1339 | CNTRLB | NPR | Negative | Negative | Negative | PHE, NMRS-South UK |
| 1488 | CNTRLB | NPR | Negative | Negative | Negative | PHE, NMRS-South UK |
| 1516 | CNTRLB | NPR | Negative | Negative | Negative | PHE, NMRS-South UK |
| 1628 | CNTRLB | NPR | Negative | Negative | Negative | PHE, NMRS-South UK |
| 1727 | CNTRLB | NPR | Negative | Negative | Negative | PHE, NMRS-South UK |
| 1728 | CNTRLB | NPR | Negative | Negative | Negative | PHE, NMRS-South UK |
| 1770 | CNTRLB | NPR | Negative | Negative | Negative | PHE, NMRS-South UK |
| 1771 | CNTRLB | NPR | Negative | Negative | Negative | PHE, NMRS-South UK |
| 1772 | CNTRLB | NPR | Negative | Negative | Negative | PHE, NMRS-South UK |
| 1773 | CNTRLB | NPR | Negative | Negative | Negative | PHE, NMRS-South UK |
| 1864 | CNTRLB | NPR | Negative | Negative | Negative | PHE, NMRS-South UK |
| 1865 | CNTRLB | NPR | Negative | Negative | Negative | PHE, NMRS-South UK |
| 1867 | CNTRLB | NPR | Negative | Negative | Negative | PHE, NMRS-South UK |
| 1868 | CNTRLB | NPR | Negative | Negative | Negative | PHE, NMRS-South UK |
| 1886 | CNTRLB | NPR | Negative | Negative | Negative | PHE, NMRS-South UK |
| 2060 | CNTRLB | NPR | Negative | Negative | Negative | PHE, NMRS-South UK |
| 2217 | CNTRLB | NPR | Negative | Negative | Negative | PHE, NMRS-South UK |
| 2296 | CNTRLB | NPR | Negative | Negative | Negative | PHE, NMRS-South UK |
| 2298 | CNTRLB | NPR | Negative | Negative | Negative | PHE, NMRS-South UK |
| 2358 | CNTRLB | NPR | Negative | Negative | Negative | PHE, NMRS-South UK |
| 2404 | CNTRLB | NPR | Negative | Negative | Negative | PHE, NMRS-South UK |
| 2405 | CNTRLB | NPR | Negative | Negative | Negative | PHE, NMRS-South UK |
| 2564 | CNTRLB | NPR | Negative | Negative | Negative | PHE, NMRS-South UK |
| 2565 | CNTRLB | NPR | Negative | Negative | Negative | PHE, NMRS-South UK |
| 2589 | CNTRLB | NPR | Negative | Negative | Negative | PHE, NMRS-South UK |
| 2609 | CNTRLB | NPR | Negative | Negative | Negative | PHE, NMRS-South UK |
| 2614 | CNTRLB | NPR | Negative | Negative | Negative | PHE, NMRS-South UK |
| 2625 | CNTRLB | NPR | Negative | Negative | Negative | PHE, NMRS-South UK |
| 2846 | CNTRLB | NPR | Negative | Negative | Negative | PHE, NMRS-South UK |
| 2859 | CNTRLB | NPR | Negative | Negative | Negative | PHE, NMRS-South UK |
| 2868 | CNTRLB | NPR | Negative | Negative | Negative | PHE, NMRS-South UK |
| 3015 | CNTRLB | NPR | Negative | Negative | Negative | PHE, NMRS-South UK |
| 3195 | CNTRLB | NPR | Negative | Negative | Negative | PHE, NMRS-South UK |
| 3239 | CNTRLB | NPR | Negative | Negative | Negative | PHE, NMRS-South UK |
| 3641 | CNTRLB | NPR | Negative | Negative | Negative | PHE, NMRS-South UK |
| 3651 | CNTRLB | NPR | Negative | Negative | Negative | PHE, NMRS-South UK |
| 3740 | CNTRLB | NPR | Negative | Negative | Negative | PHE, NMRS-South UK |
| 3767 | CNTRLB | NPR | Negative | Negative | Negative | PHE, NMRS-South UK |
| 3769 | CNTRLB | NPR | Negative | Negative | Negative | PHE, NMRS-South UK |
| 3843 | CNTRLB | NPR | Negative | Negative | Negative | PHE, NMRS-South UK |
| 3850 | CNTRLB | NPR | Negative | Negative | Negative | PHE, NMRS-South UK |
| 4011 | CNTRLB | NPR | Negative | Negative | Negative | PHE, NMRS-South UK |
| 4237 | CNTRLB | NPR | Negative | Negative | Negative | PHE, NMRS-South UK |
| 4539 | CNTRLB | NPR | Negative | Negative | Negative | PHE, NMRS-South UK |
| 4672 | CNTRLB | NPR | Negative | Negative | Negative | PHE, NMRS-South UK |
| 4685 | CNTRLB | NPR | Negative | Negative | Negative | PHE, NMRS-South UK |
| 4733 | CNTRLB | NPR | Negative | Negative | Negative | PHE, NMRS-South UK |
| 4734 | CNTRLB | NPR | Negative | Negative | Negative | PHE, NMRS-South UK |
| 4738 | CNTRLB | NPR | Negative | Negative | Negative | PHE, NMRS-South UK |
| 4744 | CNTRLB | NPR | Negative | Negative | Negative | PHE, NMRS-South UK |
| 4748 | CNTRLB | NPR | Negative | Negative | Negative | PHE, NMRS-South UK |
| 4907 | CNTRLB | NPR | Negative | Negative | Negative | PHE, NMRS-South UK |
| 4966 | CNTRLB | NPR | Negative | Negative | Negative | PHE, NMRS-South UK |
| 5072 | CNTRLB | NPR | Negative | Negative | Negative | PHE, NMRS-South UK |
| 5073 | CNTRLB | NPR | Negative | Negative | Negative | PHE, NMRS-South UK |
| 5231 | CNTRLB | NPR | Negative | Negative | Negative | PHE, NMRS-South UK |
| 5301 | CNTRLB | NPR | Negative | Negative | Negative | PHE, NMRS-South UK |
| 5432 | CNTRLB | NPR | Negative | Negative | Negative | PHE, NMRS-South UK |
| 5439 | CNTRLB | NPR | Negative | Negative | Negative | PHE, NMRS-South UK |
| 5472 | CNTRLB | NPR | Negative | Negative | Negative | PHE, NMRS-South UK |
| 5627 | CNTRLB | NPR | Negative | Negative | Negative | PHE, NMRS-South UK |
| 5723 | CNTRLB | NPR | Negative | Negative | Negative | PHE, NMRS-South UK |
| 5784 | CNTRLB | NPR | Negative | Negative | Negative | PHE, NMRS-South UK |
| 6006 | CNTRLB | NPR | Negative | Negative | Negative | PHE, NMRS-South UK |
| 6030 | CNTRLB | NPR | Negative | Negative | Negative | PHE, NMRS-South UK |
| 6097 | CNTRLB | NPR | Negative | Negative | Negative | PHE, NMRS-South UK |
| 6572 | CNTRLB | NPR | Negative | Negative | Negative | PHE, NMRS-South UK |
| 6640 | CNTRLB | NPR | Negative | Negative | Negative | PHE, NMRS-South UK |
| 6731 | CNTRLB | NPR | Negative | Negative | Negative | PHE, NMRS-South UK |
| 6909 | CNTRLB | NPR | Negative | Negative | Negative | PHE, NMRS-South UK |
| 7265 | CNTRLB | NPR | Negative | Negative | Negative | PHE, NMRS-South UK |
| 7751 | CNTRLB | NPR | Negative | Negative | Negative | PHE, NMRS-South UK |
| 8259 | CNTRLB | NPR | Negative | Negative | Negative | PHE, NMRS-South UK |
| 8365 | CNTRLB | NPR | Negative | Negative | Negative | PHE, NMRS-South UK |
| 8399 | CNTRLB | NPR | Negative | Negative | Negative | PHE, NMRS-South UK |
| 8506 | CNTRLB | NPR | Negative | Negative | Negative | PHE, NMRS-South UK |
| 8693 | CNTRLB | NPR | Negative | Negative | Negative | PHE, NMRS-South UK |
| 8746 | CNTRLB | NPR | Negative | Negative | Negative | PHE, NMRS-South UK |
| 8818 | CNTRLB | NPR | Negative | Negative | Negative | PHE, NMRS-South UK |
| 8859 | CNTRLB | NPR | Negative | Negative | Negative | PHE, NMRS-South UK |
| 8961 | CNTRLB | NPR | Negative | Negative | Negative | PHE, NMRS-South UK |
| 8979 | CNTRLB | NPR | Negative | Negative | Negative | PHE, NMRS-South UK |
| 8991 | CNTRLB | NPR | Negative | Negative | Negative | PHE, NMRS-South UK |
| 9084 | CNTRLB | NPR | Negative | Negative | Negative | PHE, NMRS-South UK |
| 9508 | CNTRLB | NPR | Negative | Negative | Negative | PHE, NMRS-South UK |
| 9521 | CNTRLB | NPR | Negative | Negative | Negative | PHE, NMRS-South UK |
| 9870 | CNTRLB | NPR | Negative | Negative | Negative | PHE, NMRS-South UK |
| 9873 | CNTRLB | NPR | Negative | Negative | Negative | PHE, NMRS-South UK |
| 19944 | CNTRLB | NPR | Negative | Negative | Negative | PHE, NMRS-South UK |
| 10094 | CNTRLB | NPR | Negative | Negative | Negative | PHE, NMRS-South UK |
| 10100 | CNTRLB | NPR | Negative | Negative | Negative | PHE, NMRS-South UK |
| 10441 | CNTRLB | NPR | Negative | Negative | Negative | PHE, NMRS-South UK |
| 73 | LTBI | NPR | Negative | Negative | **Positive** | PHE, NMRS-South UK |
| 99 | LTBI | NPR | **Positive** | **Positive** | **Positive** | PHE, NMRS-South UK |
| 342 | LTBI | NPR | Negative | **Positive** | Negative | PHE, NMRS-South UK |
| 347 | LTBI | NPR | Negative | **Positive** | Negative | PHE, NMRS-South UK |
| 513 | LTBI | NPR | **Positive** | Negative | Negative | PHE, NMRS-South UK |
| 617 | LTBI | PR | Negative | **Positive** | **Positive** | PHE, NMRS-South UK |
| 706 | LTBI | NPR | **Positive** | Negative | Negative | PHE, NMRS-South UK |
| 726 | LTBI | NPR | Negative | **Positive** | Negative | PHE, NMRS-South UK |
| 728 | LTBI | NPR | **Positive** | Negative | **Positive** | PHE, NMRS-South UK |
| 730 | LTBI | NPR | **Positive** | **Positive** | **Positive** | PHE, NMRS-South UK |
| 748 | LTBI | NPR | Negative | Negative | **Positive** | PHE, NMRS-South UK |
| 758 | LTBI | NPR | **Positive** | **Positive** | Negative | PHE, NMRS-South UK |
| 1055 | LTBI | NPR | Negative | **Positive** | **Positive** | PHE, NMRS-South UK |
| 1178 | LTBI | NPR | Negative | **Positive** | **Positive** | PHE, NMRS-South UK |
| 1192 | LTBI | NPR | Negative | Negative | **Positive** | PHE, NMRS-South UK |
| 1223 | LTBI | NPR | **Positive** | **Positive** | **Positive** | PHE, NMRS-South UK |
| 1241 | LTBI | NPR | Negative | **Positive** | Negative | PHE, NMRS-South UK |
| 1244 | LTBI | NPR | Negative | **Positive** | Negative | PHE, NMRS-South UK |
| 1249 | LTBI | NPR | **Positive** | **Positive** | Negative | PHE, NMRS-South UK |
| 1313 | LTBI | NPR | Negative | Negative | **Positive** | PHE, NMRS-South UK |
| 1314 | LTBI | NPR | Negative | Negative | **Positive** | PHE, NMRS-South UK |
| 1710 | LTBI | NPR | **Positive** | **Positive** | **Positive** | PHE, NMRS-South UK |
| 2041 | LTBI | NPR | **Positive** | **Positive** | Negative | PHE, NMRS-South UK |
| 2056 | LTBI | NPR | Negative | **Positive** | **Positive** | PHE, NMRS-South UK |
| 2064 | LTBI | NPR | Negative | Negative | **Positive** | PHE, NMRS-South UK |
| 2161 | LTBI | NPR | **Positive** | **Positive** | **Positive** | PHE, NMRS-South UK |
| 2173 | LTBI | NPR | Negative | Negative | **Positive** | PHE, NMRS-South UK |
| 2194 | LTBI | NPR | **Positive** | **Positive** | Negative | PHE, NMRS-South UK |
| 2198 | LTBI | NPR | Negative | **Positive** | Negative | PHE, NMRS-South UK |
| 2242 | LTBI | NPR | Negative | Negative | **Positive** | PHE, NMRS-South UK |
| 2354 | LTBI | NPR | **Positive** | **Positive** | Negative | PHE, NMRS-South UK |
| 2357 | LTBI | NPR | **Positive** | **Positive** | Negative | PHE, NMRS-South UK |
| 2359 | LTBI | NPR | **Positive** | Negative | Negative | PHE, NMRS-South UK |
| 2439 | LTBI | NPR | **Positive** | **Positive** | **Positive** | PHE, NMRS-South UK |
| 2462 | LTBI | NPR | Negative | **Positive** | Negative | PHE, NMRS-South UK |
| 2467 | LTBI | NPR | **Positive** | **Positive** | Negative | PHE, NMRS-South UK |
| 2488 | LTBI | NPR | **Positive** | **Positive** | **Positive** | PHE, NMRS-South UK |
| 2562 | LTBI | PR | **Positive** | **Positive** | Negative | PHE, NMRS-South UK |
| 2571 | LTBI | PR | Negative | **Positive** | Negative | PHE, NMRS-South UK |
| 2595 | LTBI | NPR | Negative | **Positive** | **Positive** | PHE, NMRS-South UK |
| 2603 | LTBI | NPR | **Positive** | Negative | Negative | PHE, NMRS-South UK |
| 2604 | LTBI | NPR | Negative | **Positive** | **Positive** | PHE, NMRS-South UK |
| 2624 | LTBI | NPR | **Positive** | **Positive** | Negative | PHE, NMRS-South UK |
| 2626 | LTBI | NPR | **Positive** | **Positive** | Negative | PHE, NMRS-South UK |
| 2636 | LTBI | NPR | Negative | **Positive** | Negative | PHE, NMRS-South UK |
| 2847 | LTBI | NPR | Negative | **Positive** | **Positive** | PHE, NMRS-South UK |
| 2863 | LTBI | NPR | **Positive** | **Positive** | **Positive** | PHE, NMRS-South UK |
| 2870 | LTBI | PR | Negative | **Positive** | **Positive** | PHE, NMRS-South UK |
| 2971 | LTBI | NPR | Negative | **Positive** | Negative | PHE, NMRS-South UK |
| 2977 | LTBI | NPR | **Positive** | Negative | Negative | PHE, NMRS-South UK |
| 2978 | LTBI | NPR | **Positive** | **Positive** | Negative | PHE, NMRS-South UK |
| 3192 | LTBI | NPR | **Positive** | Negative | Negative | PHE, NMRS-South UK |
| 3370 | LTBI | NPR | **Positive** | **Positive** | Negative | PHE, NMRS-South UK |
| 3372 | LTBI | NPR | **Positive** | Negative | **Positive** | PHE, NMRS-South UK |
| 3550 | LTBI | NPR | **Positive** | **Positive** | **Positive** | PHE, NMRS-South UK |
| 3560 | LTBI | NPR | **Positive** | **Positive** | Negative | PHE, NMRS-South UK |
| 3673 | LTBI | NPR | **Positive** | **Positive** | **Positive** | PHE, NMRS-South UK |
| 3768 | LTBI | PR | Negative | **Positive** | **Positive** | PHE, NMRS-South UK |
| 3792 | LTBI | NPR | Negative | Negative | **Positive** | PHE, NMRS-South UK |
| 3811 | LTBI | NPR | **Positive** | **Positive** | Negative | PHE, NMRS-South UK |
| 4039 | LTBI | NPR | **Positive** | Negative | **Positive** | PHE, NMRS-South UK |
| 4127 | LTBI | PR | **Positive** | Negative | **Positive** | PHE, NMRS-South UK |
| 4599 | LTBI | NPR | Negative | Negative | **Positive** | PHE, NMRS-South UK |
| 4706 | LTBI | NPR | **Positive** | Negative | **Positive** | PHE, NMRS-South UK |
| 4870 | LTBI | NPR | **Positive** | Negative | **Positive** | PHE, NMRS-South UK |
| 4931 | LTBI | NPR | Negative | **Positive** | **Positive** | PHE, NMRS-South UK |
| 4932 | LTBI | NPR | Negative | **Positive** | Negative | PHE, NMRS-South UK |
| 5076 | LTBI | NPR | **Positive** | **Positive** | Negative | PHE, NMRS-South UK |
| 5138 | LTBI | NPR | **Positive** | Negative | Negative | PHE, NMRS-South UK |
| 5180 | LTBI | NPR | **Positive** | **Positive** | **Positive** | PHE, NMRS-South UK |
| 5375 | LTBI | NPR | Negative | **Positive** | **Positive** | PHE, NMRS-South UK |
| 5664 | LTBI | NPR | Negative | **Positive** | **Positive** | PHE, NMRS-South UK |
| 5682 | LTBI | NPR | **Positive** | Negative | Negative | PHE, NMRS-South UK |
| 5684 | LTBI | NPR | Negative | Negative | **Positive** | PHE, NMRS-South UK |
| 6155 | LTBI | NPR | Negative | **Positive** | Negative | PHE, NMRS-South UK |
| 6282 | LTBI | NPR | **Positive** | Negative | **Positive** | PHE, NMRS-South UK |
| 6316 | LTBI | PR | **Positive** | Negative | **Positive** | PHE, NMRS-South UK |
| 6406 | LTBI | NPR | **Positive** | Negative | Negative | PHE, NMRS-South UK |
| 6642 | LTBI | NPR | **Positive** | **Positive** | Negative | PHE, NMRS-South UK |
| 6709 | LTBI | PR | Negative | **Positive** | Negative | PHE, NMRS-South UK |
| 6736 | LTBI | NPR | **Positive** | Negative | **Positive** | PHE, NMRS-South UK |
| 6750 | LTBI | NPR | **Positive** | Negative | Negative | PHE, NMRS-South UK |
| 6947 | LTBI | NPR | Negative | **Positive** | Negative | PHE, NMRS-South UK |
| 6995 | LTBI | NPR | **Positive** | Negative | **Positive** | PHE, NMRS-South UK |
| 7213 | LTBI | NPR | **Positive** | Negative | **Positive** | PHE, NMRS-South UK |
| 7222 | LTBI | NPR | **Positive** | Negative | **Positive** | PHE, NMRS-South UK |
| 7572 | LTBI | NPR | Negative | **Positive** | **Positive** | PHE, NMRS-South UK |
| 8118 | LTBI | NPR | **Positive** | **Positive** | Negative | PHE, NMRS-South UK |
| 8202 | LTBI | NPR | Negative | Negative | **Positive** | PHE, NMRS-South UK |
| 8212 | LTBI | NPR | Negative | **Positive** | Negative | PHE, NMRS-South UK |
| 8271 | LTBI | NPR | **Positive** | **Positive** | **Positive** | PHE, NMRS-South UK |
| 8370 | LTBI | NPR | **Positive** | Negative | **Positive** | PHE, NMRS-South UK |
| 8411 | LTBI | NPR | **Positive** | Negative | Negative | PHE, NMRS-South UK |
| 8467 | LTBI | NPR | Negative | Negative | **Positive** | PHE, NMRS-South UK |
| 8478 | LTBI | NPR | **Positive** | **Positive** | **Positive** | PHE, NMRS-South UK |
| 8685 | LTBI | NPR | **Positive** | Negative | **Positive** | PHE, NMRS-South UK |
| 8970 | LTBI | NPR | **Positive** | Negative | Negative | PHE, NMRS-South UK |
| 8982 | LTBI | NPR | Negative | Negative | **Positive** | PHE, NMRS-South UK |
| 8998 | LTBI | NPR | Negative | **Positive** | **Positive** | PHE, NMRS-South UK |
| 9123 | LTBI | NPR | **Positive** | **Positive** | **Positive** | PHE, NMRS-South UK |
| 9148 | LTBI | NPR | Negative | Negative | **Positive** | PHE, NMRS-South UK |
| 9476 | LTBI | NPR | **Positive** | Negative | Negative | PHE, NMRS-South UK |
| 9974 | LTBI | NPR | Negative | Negative | **Positive** | PHE, NMRS-South UK |
| 10714 | LTBI | NPR | **Positive** | Negative | Negative | PHE, NMRS-South UK |
| BAL072 | UKPTB | NA | ND | ND | ND | UCL, Royal Free NHS UK |
| BAL073 | UKPTB | NA | ND | ND | ND | UCL, Royal Free NHS UK |
| BAL074 | UKPTB | NA | ND | ND | ND | UCL, Royal Free NHS UK |
| BAL075 | UKPTB | NA | ND | ND | ND | UCL, Royal Free NHS UK |
| BAL077 | UKPTB | NA | ND | ND | ND | UCL, Royal Free NHS UK |
| BAL079 | UKPTB | NA | ND | ND | ND | UCL, Royal Free NHS UK |
| BAL080 | UKPTB | NA | ND | ND | ND | UCL, Royal Free NHS UK |
| BAL082 | UKPTB | NA | ND | ND | ND | UCL, Royal Free NHS UK |
| BAL083 | UKPTB | NA | ND | ND | ND | UCL, Royal Free NHS UK |
| BAL086 | UKPTB | NA | ND | ND | ND | UCL, Royal Free NHS UK |
| BAL087 | UKPTB | NA | ND | ND | ND | UCL, Royal Free NHS UK |
| BAL088 | UKPTB | NA | ND | ND | ND | UCL, Royal Free NHS UK |
| BAL089 | UKPTB | NA | ND | ND | ND | UCL, Royal Free NHS UK |
| BAL091 | UKPTB | NA | ND | ND | ND | UCL, Royal Free NHS UK |
| BAL092 | UKPTB | NA | ND | ND | ND | UCL, Royal Free NHS UK |
| BAL093 | UKPTB | NA | ND | ND | ND | UCL, Royal Free NHS UK |
| BAL094 | UKPTB | NA | ND | ND | ND | UCL, Royal Free NHS UK |
| EA16 | UKPTB | NA | ND | ND | ND | UCL, Royal Free NHS UK |
| EA17 | UKPTB | NA | ND | ND | ND | UCL, Royal Free NHS UK |
| EA22 | UKPTB | NA | ND | ND | ND | UCL, Royal Free NHS UK |
| EA25 | UKPTB | NA | ND | ND | ND | UCL, Royal Free NHS UK |
| EA26 | UKPTB | NA | ND | ND | ND | UCL, Royal Free NHS UK |
| EA27 | UKPTB | NA | ND | ND | ND | UCL, Royal Free NHS UK |
| EA29 | UKPTB | NA | ND | ND | ND | UCL, Royal Free NHS UK |
| EA30 | UKPTB | NA | ND | ND | ND | UCL, Royal Free NHS UK |
| EA34 | UKPTB | NA | ND | ND | ND | UCL, Royal Free NHS UK |
| EA35 | UKPTB | NA | ND | ND | ND | UCL, Royal Free NHS UK |
| EA36 | UKPTB | NA | ND | ND | ND | UCL, Royal Free NHS UK |
| EA37 | UKPTB | NA | ND | ND | ND | UCL, Royal Free NHS UK |
| EA38 | UKPTB | NA | ND | ND | ND | UCL, Royal Free NHS UK |
| EA39 | UKPTB | NA | ND | ND | ND | UCL, Royal Free NHS UK |
| EA40 | UKPTB | NA | ND | ND | ND | UCL, Royal Free NHS UK |
| EA41 | UKPTB | NA | ND | ND | ND | UCL, Royal Free NHS UK |
| EA43 | UKPTB | NA | ND | ND | ND | UCL, Royal Free NHS UK |
| EA44 | UKPTB | NA | ND | ND | ND | UCL, Royal Free NHS UK |
| EA45 | UKPTB | NA | ND | ND | ND | UCL, Royal Free NHS UK |
| EA49 | UKPTB | NA | ND | ND | ND | UCL, Royal Free NHS UK |
| EBUS018 | UKPTB | NA | ND | ND | ND | UCL, Royal Free NHS UK |
| EBUS020 | UKPTB | NA | ND | ND | ND | UCL, Royal Free NHS UK |
| EBUS021 | UKPTB | NA | ND | ND | ND | UCL, Royal Free NHS UK |
| EBUS023 | UKPTB | NA | ND | ND | ND | UCL, Royal Free NHS UK |
| EBUS024 | UKPTB | NA | ND | ND | ND | UCL, Royal Free NHS UK |
| EBUS028 | UKPTB | NA | ND | ND | ND | UCL, Royal Free NHS UK |
| EBUS031 | UKPTB | NA | ND | ND | ND | UCL, Royal Free NHS UK |
| EBUS032 | UKPTB | NA | ND | ND | ND | UCL, Royal Free NHS UK |
| EBUS033 | UKPTB | NA | ND | ND | ND | UCL, Royal Free NHS UK |
| EBUS042 | UKPTB | NA | ND | ND | ND | UCL, Royal Free NHS UK |
| EBUS046 | UKPTB | NA | ND | ND | ND | UCL, Royal Free NHS UK |
| EBUS047 | UKPTB | NA | ND | ND | ND | UCL, Royal Free NHS UK |
| EBUS050 | UKPTB | NA | ND | ND | ND | UCL, Royal Free NHS UK |
| EBUS053 | UKPTB | NA | ND | ND | ND | UCL, Royal Free NHS UK |
| EBUS054 | UKPTB | NA | ND | ND | ND | UCL, Royal Free NHS UK |
| EBUS055 | UKPTB | NA | ND | ND | ND | UCL, Royal Free NHS UK |
| EBUS056 | UKPTB | NA | ND | ND | ND | UCL, Royal Free NHS UK |
| EBUS057 | UKPTB | NA | ND | ND | ND | UCL, Royal Free NHS UK |
| EBUS058 | UKPTB | NA | ND | ND | ND | UCL, Royal Free NHS UK |
| EBUS059 | UKPTB | NA | ND | ND | ND | UCL, Royal Free NHS UK |
| EBUS060 | UKPTB | NA | ND | ND | ND | UCL, Royal Free NHS UK |
| EBUS061 | UKPTB | NA | ND | ND | ND | UCL, Royal Free NHS UK |
| EBUS062 | UKPTB | NA | ND | ND | ND | UCL, Royal Free NHS UK |
| EBUS063 | UKPTB | NA | ND | ND | ND | UCL, Royal Free NHS UK |
| EBUS064 | UKPTB | NA | ND | ND | ND | UCL, Royal Free NHS UK |
| EBUS066 | UKPTB | NA | ND | ND | ND | UCL, Royal Free NHS UK **63** |
| 1 | IEPTB | NA | ND | ND | ND | JIPMER, Pondicherry, India |
| 2 | IEPTB | NA | ND | ND | ND | JIPMER, Pondicherry, India |
| 4 | IEPTB | NA | ND | ND | ND | JIPMER, Pondicherry, India |
| 7 | IEPTB | NA | ND | ND | ND | JIPMER, Pondicherry, India |
| 8 | IEPTB | NA | ND | ND | ND | JIPMER, Pondicherry, India |
| 9 | IEPTB | NA | ND | ND | ND | JIPMER, Pondicherry, India |
| 10 | IEPTB | NA | ND | ND | ND | JIPMER, Pondicherry, India |
| 11 | IEPTB | NA | ND | ND | ND | JIPMER, Pondicherry, India |
| 12 | IEPTB | NA | ND | ND | ND | JIPMER, Pondicherry, India |
| 13 | IEPTB | NA | ND | ND | ND | JIPMER, Pondicherry, India |
| 14 | IEPTB | NA | ND | ND | ND | JIPMER, Pondicherry, India |
| 15 | IEPTB | NA | ND | ND | ND | JIPMER, Pondicherry, India |
| 16 | IEPTB | NA | ND | ND | ND | JIPMER, Pondicherry, India |
| 17 | IEPTB | NA | ND | ND | ND | JIPMER, Pondicherry, India |
| 18 | IEPTB | NA | ND | ND | ND | JIPMER, Pondicherry, India |
| 19 | IEPTB | NA | ND | ND | ND | JIPMER, Pondicherry, India |
| 20 | IEPTB | NA | ND | ND | ND | JIPMER, Pondicherry, India |
| EP1 | IEPTB | NA | ND | ND | ND | AIIMS, New Delhi, India |
| EP2 | IEPTB | NA | ND | ND | ND | AIIMS, New Delhi, India |
| EP3 | IEPTB | NA | ND | ND | ND | AIIMS, New Delhi, India |
| EP4 | IEPTB | NA | ND | ND | ND | AIIMS, New Delhi, India |
| EP5 | IEPTB | NA | ND | ND | ND | AIIMS, New Delhi, India |
| EP6 | IEPTB | NA | ND | ND | ND | AIIMS, New Delhi, India |
| EP7 | IEPTB | NA | ND | ND | ND | AIIMS, New Delhi, India |
| EP8 | IEPTB | NA | ND | ND | ND | AIIMS, New Delhi, India |
| EP9 | IEPTB | NA | ND | ND | ND | AIIMS, New Delhi, India |
| EP10 | IEPTB | NA | ND | ND | ND | AIIMS, New Delhi, India |
| EP11 | IEPTB | NA | ND | ND | ND | AIIMS, New Delhi, India |
| EP12 | IEPTB | NA | ND | ND | ND | AIIMS, New Delhi, India |
| EP13 | IEPTB | NA | ND | ND | ND | AIIMS, New Delhi, India |
| EP14 | IEPTB | NA | ND | ND | ND | AIIMS, New Delhi, India |
| EP15 | IEPTB | NA | ND | ND | ND | AIIMS, New Delhi, India |
| EP16 | IEPTB | NA | ND | ND | ND | AIIMS, New Delhi, India |
| EP17 | IEPTB | NA | ND | ND | ND | AIIMS, New Delhi, India |
| EP18 | IEPTB | NA | ND | ND | ND | AIIMS, New Delhi, India |
| EP19 | IEPTB | NA | ND | ND | ND | AIIMS, New Delhi, India |
| EP20 | IEPTB | NA | ND | ND | ND | AIIMS, New Delhi, India |
| EP21 | IEPTB | NA | ND | ND | ND | AIIMS, New Delhi, India |
| EP22 | IEPTB | NA | ND | ND | ND | AIIMS, New Delhi, India |
| EP23 | IEPTB | NA | ND | ND | ND | AIIMS, New Delhi, India |
| EP24 | IEPTB | NA | ND | ND | ND | AIIMS, New Delhi, India |
| EP25 | IEPTB | NA | ND | ND | ND | AIIMS, New Delhi, India |
| P1 | IPTB | NA | ND | ND | ND | AIIMS, New Delhi, India |
| P2 | IPTB | NA | ND | ND | ND | AIIMS, New Delhi, India |
| P3 | IPTB | NA | ND | ND | ND | AIIMS, New Delhi, India |
| P4 | IPTB | NA | ND | ND | ND | AIIMS, New Delhi, India |
| P5 | IPTB | NA | ND | ND | ND | AIIMS, New Delhi, India |
| P6 | IPTB | NA | ND | ND | ND | AIIMS, New Delhi, India |
| P7 | IPTB | NA | ND | ND | ND | AIIMS, New Delhi, India |
| P8 | IPTB | NA | ND | ND | ND | AIIMS, New Delhi, India |
| P9 | IPTB | NA | ND | ND | ND | AIIMS, New Delhi, India |
| P10 | IPTB | NA | ND | ND | ND | AIIMS, New Delhi, India |
| P11 | IPTB | NA | ND | ND | ND | AIIMS, New Delhi, India |
| P12 | IPTB | NA | ND | ND | ND | AIIMS, New Delhi, India |
| P13 | IPTB | NA | ND | ND | ND | AIIMS, New Delhi, India |
| P14 | IPTB | NA | ND | ND | ND | AIIMS, New Delhi, India |
| P15 | IPTB | NA | ND | ND | ND | AIIMS, New Delhi, India |
| P16 | IPTB | NA | ND | ND | ND | AIIMS, New Delhi, India |
| P17 | IPTB | NA | ND | ND | ND | AIIMS, New Delhi, India |
| P18 | IPTB | NA | ND | ND | ND | AIIMS, New Delhi, India |
| P19 | IPTB | NA | ND | ND | ND | AIIMS, New Delhi, India |
| P20 | IPTB | NA | ND | ND | ND | AIIMS, New Delhi, India |
| P21 | IPTB | NA | ND | ND | ND | AIIMS, New Delhi, India |
| P22 | IPTB | NA | ND | ND | ND | AIIMS, New Delhi, India |
| P23 | IPTB | NA | ND | ND | ND | AIIMS, New Delhi, India |
| P24 | IPTB | NA | ND | ND | ND | AIIMS, New Delhi, India |
| P25 | IPTB | NA | ND | ND | ND | AIIMS, New Delhi, India |
| P26 | IPTB | NA | ND | ND | ND | AIIMS, New Delhi, India |
| P27 | IPTB | NA | ND | ND | ND | AIIMS, New Delhi, India |
| P28 | IPTB | NA | ND | ND | ND | AIIMS, New Delhi, India |
| P29 | IPTB | NA | ND | ND | ND | AIIMS, New Delhi, India |
| P30 | IPTB | NA | ND | ND | ND | AIIMS, New Delhi, India |
| P31 | IPTB | NA | ND | ND | ND | AIIMS, New Delhi, India |
| P32 | IPTB | NA | ND | ND | ND | AIIMS, New Delhi, India |
| P33 | IPTB | NA | ND | ND | ND | AIIMS, New Delhi, India |
| P34 | IPTB | NA | ND | ND | ND | AIIMS, New Delhi, India |
| P35 | IPTB | NA | ND | ND | ND | AIIMS, New Delhi, India |
| P36 | IPTB | NA | ND | ND | ND | AIIMS, New Delhi, India |
| P37 | IPTB | NA | ND | ND | ND | AIIMS, New Delhi, India |
| P38 | IPTB | NA | ND | ND | ND | AIIMS, New Delhi, India |
| P39 | IPTB | NA | ND | ND | ND | AIIMS, New Delhi, India |
| P40 | IPTB | NA | ND | ND | ND | AIIMS, New Delhi, India |
| P41 | IPTB | NA | ND | ND | ND | AIIMS, New Delhi, India |
| P42 | IPTB | NA | ND | ND | ND | AIIMS, New Delhi, India |
| P43 | IPTB | NA | ND | ND | ND | AIIMS, New Delhi, India |
| P44 | IPTB | NA | ND | ND | ND | AIIMS, New Delhi, India |
| P45 | IPTB | NA | ND | ND | ND | AIIMS, New Delhi, India |
| P46 | IPTB | NA | ND | ND | ND | AIIMS, New Delhi, India |
| P47 | IPTB | NA | ND | ND | ND | AIIMS, New Delhi, India |

**Table S1.2 Number of patients or controls recruited per collaborating site**

| **Recruitment Site** | **Group Sample Number** | | | | | |
| --- | --- | --- | --- | --- | --- | --- |
|  | **CNTRLA** | **CNTRLB** | **LTBI** | **IEPTB** | **UKPTB** | **IPTB** |
| Public Health England Porton Down, Salisbury | **20** |  |  |  |  |  |
| Public Health England Colindale, London &  University College London |  | **103** | **106** |  |  |  |
| St. Thomas’s and Royal Free  Hospitals, London |  |  |  |  | **63** |  |
| The All India Institute for Medical Sciences, New Delhi |  |  |  | **25** |  | **47** |
| The Jawaharlal Institute of Postgraduate Medical Education & Research, Pondicherry |  |  |  | **17** |  |  |

**Table S1.3 Number of Participants per PREDICT TB Latent TB and Control Study Groups Classified by TST IGRA Status and Progression to Active TB**

| **Group** | **Quantiferon** | **TSPOT** | **TST skin test** | **Numbers Individual per Group** | **Total Number Progressed to Active Tuberculosis** |
| --- | --- | --- | --- | --- | --- |
| **1** | **+** | **+** | **+** | **14** | **0** |
| **2** | **+** | **-** | **+** | **14** | **2** |
| **3** | **-** | **+** | **+** | **14** | **3** |
| **4** | **+** | **-** | **-** | **15** | **0** |
| **5** | **-** | **+** | **-** | **15** | **2** |
| **6** | **+** | **+** | **-** | **18** | **1** |
| **7** | **-** | **-** | **+** | **16** | **0** |
| **8** | **-** | **-** | **-** | **103** | **ND** |
| **Total Number Latent TB n = 106** | | | | | |
| **Total Number Latent TB progressed to active Tuberculosis n = 8 (7.55%)** | | | | | |
| **Total Number Controls n = 103** | | | | | |

**Table S1.4 Gene Entities Validated using Roche Real-time Ready qPCR Assays in Study**

| **Gene** | **Gene Name** | **Biological Function** | **Gene ID** | **Roche RTR Configuration ID** | **Roche RTR Assay ID** |
| --- | --- | --- | --- | --- | --- |
| ABCF2 | ATP-binding cassette, sub-family F (GCN20), member 2 | transport of various molecules across extra- and intracellular membranes | 10061 | 59386 |  |
| AIM2 | (PYHIN4) absent in melanoma 2 | Interferon-gamma induced, may control cell proliferation | 9447 | 70479 | 137959 |
| ALPK1 | alpha-kinase 1 | kinase responsible for TIFA oligomerization and IL-8 expression in response to infection | 80216 | 59386 | 106136 |
| ANKRD22 | ankyrin repeat domain 22 | promotes cell proliferation by up-regulating expression of E2F1 which enhances cell cycle progression | 118932 | 70479 |  |
| APBB1IP | Rap1-interacting molecule (RIAM) , amyloid beta (A4) precursor protein-binding, family B, member 1 interacting protein | Immune function, adhesion | 54518 | 59386 | 144712 |
| BAZ1A | bromodomain adjacent to zinc finger domain, 1A | Accessory subunit of the ATP-dependent chromatin assembly factor (ACF), a member of the ISWI ('imitation switch') family of chromatin remodelling complexes | 960 | 59386 | 129645 |
| BST1 | bone marrow stromal cell antigen 1 | Immune function | 683 | 59377 | 143515 |
| C1QB | complement component 1, q subcomponent, B chain | associates with C1r and C1s to yield the first component of the serum complement system | 713 | 70479 | 140622 |
| C6orf150 | (CGAS) cyclic GMP-AMP synthase | Innate immune signalling, cytosolic DNA sensing | 115004 | 59386 | 142684 |
| CALCOCO2 | calcium binding and coiled-coil domain 2 | Mediates macro-autophagy in innate immunity | 10241 | 59386 | 127932 |
| CD274 | (PD- L1) CD274 molecule | immune inhibitory receptor ligand | 29126 | 59386 | 104030 |
| CD52 | CD52 molecule | Natural killer cell function | 1043 | 59386 | 127224 |
| CD96 | CD96 molecule | Innate and adaptive immune system | 10225 | 59386 | 147106 |
| CDH23 | cadherin related protein 23 | calcium dependent cell-cell adhesion glycoprotein | 64072 | 59377 | 144804 |
| CLIC1 | Chloride intracellular channel protein 1 | Localised principally to cell nucleus, exhibits both nuclear and plasma membrane chloride ion channel activity | 1192 | 59377 |  |
| CPVL | carboxypeptidase vitellogenic like | strong sequence similarity to serine carboxypeptidases, function unknown | 54504 | 59377 | 109746 |
| CREG1 | cellular repressor of E1A stimulated genes 1 | Immune function | 8804 | 59377 | 120091 |
| DEFB128 | defensin, beta 128 | antimicrobial cysteine-rich cationic polypeptide that is important in the immunologic response to invading microorganisms | 245939 | 70479 |  |
| DOCK9 | dedicator of cytokinesis 9 | Regulator of small guanosine triphosphatases (GTPases) | 23348 | 59386 | 144808 |
| DUSP6 | dual specificity phosphatase 6 | Dual specificity protein phosphatase subfamily. Dephosphorylation of phosphoserine/ threonine and phosphotyrosine residues, negatively regulate members of the mitogen-activated protein (MAP) kinase superfamily (MAPK/ERK, SAPK/JNK, p38) | 3072 | 59386 | 105074 |
| EPSTI1 | epithelial stromal interaction 1 | upregulated by direct binding of the Kruppel like factor 8 protein to its promoter sequences, upregulated in systemic lupus erythematosus (SLE) lymphocytes | 94240 | 70479 | 143623 |
| FNBP1L | (Toca-1) formin binding protein 1-like | involved in a pathway that links cell surface signals to the actin cytoskeleton, plays an essential role in antibacterial autophagy | 54874 | 70479 | 145424 |
| FYB | FYN binding protein 1 | adapter for FYN protein and LCP2 signaling cascades in T-cells, platelet activation | 2533 | 59377 | 144710 |
| GBP1 | guanylate binding protein 1, interferon-inducible | Immune multifunctional, type II IFN and NOD-like receptor signalling | 2633 | 59386 | 103520 |
| GBP2 | guanylate binding protein 2, interferon-inducible | Interferon induced, regulator of dendritic cell maturation, inhibitor of intracellular pathogen replication e.g. T. gondii | 14469 | 70479 | 108390 |
| GBP5 | guanylate binding protein 5 | acts as an activator of NLRP3 inflammasome assembly and has a role in innate immunity and inflammation | 115362 | 70479 | 103978 |
| GK | glycerol kinase | A key enzyme in the regulation of glycerol uptake and metabolism | 2710 | 70479 | 147305 |
| GPR141 | G protein-coupled receptor 141 | member of the rhodopsin family of G protein-coupled receptors (GPRs) | 353345 | 70479 | 145422 |
| GTF2B | general transcription factor IIB | transcription initiation by RNA polymerase II cofactor | 2959 | 59377 | 111741 |
| HERC2 | HECT and RLD domain containing E3 ubiquitin protein ligase 2 | Antigen processing: Ubiquitination & Proteasome degradation | 8924 | 59377 | 125362 |
| HLA-B | major histocompatibility complex, class I, B | HLA class I heavy chain, antigen presentation | 3106 | 59377 | 135874 |
| HLA-F | major histocompatibility complex, class I, F | HLA class I heavy chain forms a heterodimer with a beta-2 microglobulin light chain, localized in the endoplasmic reticulum and Golgi apparatus | 3134 | 59377 | 125340 |
| IFIT3 | interferon-induced protein with tetratricopeptide repeats 3 | Interferon induced immune process | 3437 | 59386 | 128097 |
| IFITM3 | interferon induced transmembrane protein 3 | interferon-induced membrane protein | 10410 | 59377 | 145233 |
| IL8 | interleukin 8 (CXCL8) | Primarily secreted by neutrophils, chemotactic factor which guides neutrophils to sites of infection, also a potent angiogenic factor | 3576 | 59386 | 103136 |
| IRF1 | interferon regulatory factor 1 | transcriptional regulator of genes involved in innate and adaptive immune responses | 3659 | 59377 | 144798 |
| JAK2 | Janus kinase 2 | Protein tyrosine kinase involved in a specific subset of cytokine receptor signalling pathways, found to be constitutively associated with the prolactin receptor and is required for responses to gamma interferon | 3717 | 70479 | 105921 |
| KLRAP1 | killer cell lectin-like receptor subfamily A pseudogene 1, killer cell lectin-like receptor subfamily A, member 1 | Transcribed pseudogene | 10748 | 59386 | 144812 |
| LAP3 | leucine aminopeptidase 3 | Regulator of cell growth, proliferation and migration | 51056 | 70479 | 108390 |
| LDLR | low density lipoprotein receptor | Endocytosis, lipid transport | 3949 | 59386 | 113016 |
| LGALS3BP | lectin, galactoside-binding, soluble, 3 binding protein | immune response associated with natural killer (NK) and lymphokine-activated killer (LAK) cell cytotoxicity, binds specifically to human macrophage-associated Mac-2 and also galectin 1 | 3959 | 59386 | 135937 |
| LOC400759 | (GBP1P1) interferon-induced guanylate-binding protein 1 pseudogene | Interferon induced, immune system | 400759 | 59377 | 144711 |
| LYN | LYN proto-oncogene, Src family tyrosine kinase | Immune function, dendritic cell responses, negative regulator of BIM apoptotic regulator | 4067 | 59377 | 144706 |
| LYPLA1 | lysophospholipase I | The encoded protein functions as a homodimer, exhibiting both depalmitoylating as well as lysophospholipase activity, may be involved in Ras localization and signalling | 6737 | 59386 | 144717 |
| MGST2 | microsomal glutathione S-transferase 2 | Involved in the production of leukotrienes and prostaglandin E, catalyzes the conjugation of leukotriene A4 and reduced glutathione to produce leukotriene C4 | 4258 | 59386 | 118047 |
| MMP9 | matrix metallopeptidase 9 | Breakdown of extracellular matrix in normal physiological processes, may also be involved in IL-8-induced mobilization of hematopoietic progenitor cells from bone marrow | 4318 | 70479 | 139820 |
| MVP | major vault protein | major component of multi-subunit ribonucleoprotein vault structures | 9961 | 59377 | 112104 |
| NCF1C | neutrophil cytosolic factor 1C pseudogene | One of three highly similar pseudogenes located proximal to the neutrophil cytosolic factor 1 (NCF1) gene encoding the 47 kDa cytosolic subunit of neutrophil NADPH oxidase, which produces superoxide anion | 654817 | 70479 | 145443 |
| NELL2 | neural EGFL like 2 | Cell growth and differentiation | 4753 | 59386 | 139015 |
| OLFML3 | olfactomedin-like 3 | secreted extracellular matrix glycoprotein which facilitates protein-protein interactions, cell adhesion, and intercellular interactions | 56944 | 59386 |  |
| PARP9 | poly (ADP-ribose) polymerase family, member 9 | Regulator of macrophage activation via STAT1 phosphorylation, B cell regulator etc. | 83666 | 70479 | 122042 |
| PF4V1 | (CXCL4L1, CXCL4V1) platelet factor 4 variant 1 | The protein encoded by this gene is a chemokine highly similar to platelet factor 4. The encoded protein displays a strong antiangiogenic function and is regulated by chemokine (C-X-C motif) receptor 3 | 5197 | 70479 | 145708 |
| PSMB9 | proteasome subunit beta 9 | induced by gamma interferon and replaces catalytic subunit 1 (proteasome beta 6 subunit) in the immunoproteasome, processing of MHC Class I peptides | 5698 | 59377 | 144683 |
| PSMD6 | proteasome 26S subunit, non-ATPase 6 | subunit of the 26S proteasome, involved in the ATP-dependent degradation of ubiquinated proteins | 9861 | 59377 | 137899 |
| S100A11 | S100 calcium binding protein A11 | Possible function in motility, invasion, and tubulin polymerization | 6282 | 59377 | 115330 |
| SAMD9L | sterile alpha motif domain containing 9-like | Cytoplasmic protein that acts as a tumor suppressor but also plays a key role in cell proliferation and the innate immune response to viral infection | 219285 | 70479 | 103641 |
| SERPINB1 | serpin family B member 1 | inhibits neutrophil-derived proteinases neutrophil elastase, cathepsin G, and proteinase-3 | 1992 | 59377 | 124773 |
| SIRPB2 | signal-regulatory protein beta 2 | Expressed mainly by myeloid cells, role in immune-cell regulation | 284759 | 70479 | 124326 |
| SLC14A1 | solute carrier family 14 (urea transporter), member 1 | membrane channel protein that mediates the rapid transmembrane transport of urea and participates in urine concentration |  | 70479 |  |
| SNX10 | sorting nexin 10 | plays a role in regulating endosome homeostasis | 29887 | 59377 | 139740 |
| SPAST | Spastin | Microtubule binding/regulation, motility | 6683 | 59386 | 113242 |
| STAT1 | signal transducer and activator of transcription 1, 91kDa | transcription activator, can be activated by various ligands including interferon-alpha, interferon-gamma, EGF, PDGF and IL6, mediates expression of a variety of genes thought to be important for cell viability in response to different cell stimuli and pathogens | 6772 | 70479 | 101180 |
| TAF10 | TATA-box binding protein associated factor 10 | RNA polymerase II transcription factor IID (TFIID) associated factor | 6881 | 59377 | 120238 |
| TAPBP | tapasin isoform 1 precursor | transmembrane glycoprotein which mediates interaction between newly assembled major histocompatibility complex (MHC) class I molecules and the transporter associated with antigen processing (TAP), required for transport of antigenic peptides across the endoplasmic reticulum membrane and essential for optimal peptide loading on the MHC class I molecule. | 6892 | 70479 | 138030 |
| TBC1D3B | TBC1 domain family, member 3B | stimulates the intrinsic GTPase activity of RAB5A, an essential actor in early endosome trafficking | 414059 | 70479 | 145420 |
| TICAM2 | toll like receptor adaptor molecule 2 | Immune function, (TIR) domain-containing adaptor protein involved in Toll receptor signaling | 353376 | 59377 | 144681 |
| TLR6 | toll-like receptor 6 | member of the Toll-like receptor (TLR) family which plays a fundamental role in pathogen recognition and activation of innate immunity, functionally interacts with toll-like receptor 2 to mediate cellular response to bacterial lipoproteins | 10333 | 70479 | 144018 |
| TMEM49 | (VMP1) vacuole membrane protein 1 | regulatory role in the process of autophagy | 81671 | 59377 | 119966 |
| TRIM25 | tripartite motif-containing 25 | Transcription factor, critical for sustained and amplified retinoic acid-inducible gene 1 (RIG-I)-induced type I interferon expression | 7706 | 70479 | 119435 |
| WAC | WW domain containing adaptor with coiled-coil | WW domain protein, function unknown | 51322 | 59377 | 138653 |
| WARS | tryptophanyl-tRNA synthetase | Induced by interferon, primary innate immune defence | 7453 | 59386 | 144819 |
| WSB1 | WD repeat and SOCS box containing 1 | Adaptive immunity, Antigen processing | 26118 | 59386 | 131297 |

**Table S1.5 Biomarkers differentially expressed between different control and disease groups from ANOVA SNK analysis**

| **Group** | **CNTRLA** | | **CNTRLB** | **LTBI** | | **IEPTB** | | **UKPTB** | | | **IPTB** |
| --- | --- | --- | --- | --- | --- | --- | --- | --- | --- | --- | --- |
| **CNTRLA** | **NA** | **S100A11** | | | **IFITM3** | | **GBP1**  **GBP2**  **GBP5**  **IFITM3**  **NCF1C**  **S100A11** | | **GBP1**  **GBP5**  **IFITM3**  **IRF1**  **STAT1**  **S100A11** | **GBP1**  **GBP2**  **GBP5**  **HLA-B**  **IFIT3**  **IFITM3**  **IRF1**  **LOC400759**  **NCF1C**  **S100A11**  **SAMD9L**  **TAF10**  **TAPBP**  **TRIM25** | |
| **CNTRLB** |  | **NA** | | | **NONE** | | **GBP1**  **GBP2**  **GBP5**  **IFIT3**  **NCF1C** | | **GBP1**  **IRF1**  **PF4V1**  **STAT1** | **GBP1**  **GBP2**  **GBP5**  **HLA-B**  **IFIT3**  **IFITM3**  **IRF1**  **LOC400759**  **NCF1C**  **SAMD9L**  **TAF10**  **TRIM25** | |
| **LTBI** |  |  | | | **NA** | | **GBP1**  **GBP2**  **GBP5** | | **CD52**  **GBP1**  **IRF1**  **STAT1** | **GBP1**  **GBP2**  **GBP5**  **HLA-B**  **IFIT3**  **IFITM3**  **IRF1**  **LOC400759**  **NCF1C**  **S100A11**  **SAMD9L**  **TAF10**  **TAPBP**  **TRIM25** | |
| **IEPTB** |  |  | | |  | | **NA** | | **GBP1**  **GBP2**  **IRF1**  **STAT1** | **GBP5**  **HLA-B**  **IFITM3**  **IRF1**  **LOC400759**  **NCF1C**  **TAF10**  **TRIM25** | |
| **UKPTB** |  |  | | |  | |  | | **NA** | **CD52**  **GBP1**  **GBP2**  **GBP5**  **HLA-B**  **IFITM3**  **LOC400759**  **NCF1C**  **PF4V1**  **SAMD9L**  **STAT1** | |
| **IPTB** |  |  | | |  | |  | |  | **NA** | |

**Table S1.6** **ROC/AUC values from pairwise comparisons for single biomarkers between control, latent and active disease groups**

| **Group** | **Biomarker ROC Curve Value** | | | | | | | | | | | | | | | | |
| --- | --- | --- | --- | --- | --- | --- | --- | --- | --- | --- | --- | --- | --- | --- | --- | --- | --- |
|  | **CD52** | **GBP1** | **GBP2** | **GBP5** | **HLA-B** | **IFIT3** | **IFITM3** | **IRF1** | **LOC400759** | **NCF1C** | **PF4V1** | **S100A11** | **SAMD9L** | **STAT1** | **TAPBP** | **TAF10** | **TRIM25** |
| **CNTRLA vs UKPTB** | 0.561 | **0.907** | **0.952** | 0.738 | 0.574 | **0.999** | **0.933** | **0.99** | **0.956** | 0.869 | 0.667 | **0.998** | 0.725 | 0.859 | 0.887 | ***0.326*** | 0.442 |
| **CNTRLA vs IEPTB** | 0.358 | **0.918** | **0.905** | 0.740 | 0.558 | **0.960** | 0.826 | 0.785 | 0.786 | 0.880 | 0.345 | 0.866 | 0.836 | 0.427 | 0.848 | ***0.089*** | 0.654 |
| **CNTRLA vs IPTB** | ***0.167*** | **0.973** | **0.962** | **0.905** | 0.576 | **0.971** | **0.983** | **0.993** | **0.979** | **0.953** | ***0.273*** | **0.995** | **0.933** | 0.736 | **0.952** | ***0.143*** | **0.919** |
| **UKPTB vs IPTB** | ***0.182*** | 0.687 | 0.656 | 0.752 | 0.691 | 0.539 | 0.702 | 0.536 | 0.699 | 0.759 | ***0.208*** | 0.510 | 0.800 | 0.317 | 0.637 | ***0.243*** | 0.797 |
| **IEPTB vs UKPTB** | 0.651 | 0.409 | 0.496 | 0.562 | 0.509 | 0.470 | 0.559 | 0.698 | 0.588 | 0.410 | 0.724 | 0.579 | 0.324 | 0.833 | 0.459 | 0.834 | 0.338 |
| **IEPTB vs IPTB** | 0.391 | 0.579 | 0.638 | 0.672 | 0.623 | 0.503 | 0.735 | 0.735 | 0.752 | 0.669 | 0.455 | 0.583 | 0.651 | 0.721 | 0.570 | 0.671 | 0.696 |
| **CNTRLB vs UKPTB** | 0.743 | 0.782 | 0.847 | 0.774 | 0.534 | **0.916** | 0.326 | 0.824 | 0.850 | 0.448 | 0.823 | 0.667 | 0.804 | 0.851 | 0.769 | 0.610 | 0.333 |
| **CNTRLB vs IEPTB** | 0.645 | **0.906** | 0.822 | 0.777 | 0.547 | 0.884 | 0.575 | 0.597 | 0.677 | 0.648 | 0.538 | 0.542 | 0.890 | 0.409 | 0.747 | ***0.221*** | 0.536 |
| **CNTRLB vs IPTB** | 0.553 | **0.961** | 0.895 | **0.925** | 0.678 | 0.897 | 0.852 | 0.863 | **0.927** | 0.826 | 0.496 | 0.647 | **0.955** | 0.721 | 0.861 | ***0.322*** | 0.799 |
| **LTBI vs UKPTB** | 0.853 | 0.773 | 0.845 | 0.753 | 0.460 | 0.861 | 0.430 | 0.851 | 0.824 | 0.601 | 0.813 | 0.787 | **0.945** | 0.774 | 0.841 | 0.527 | 0.332 |
| **LTBI vs IEPTB** | 0.717 | 0.817 | 0.821 | 0.757 | 0.452 | 0.846 | 0.383 | 0.614 | 0.554 | 0.673 | 0.526 | 0.627 | **0.871** | 0.346 | 0.831 | ***0.162*** | 0.550 |
| **LTBI vs IPTB** | 0.601 | 0.895 | **0.901** | **0.918** | 0.585 | 0.854 | 0.662 | 0.845 | **0.913** | 0.832 | 0.488 | 0.756 | **0.951** | 0.621 | **0.908** | ***0.219*** | 0.833 |
| **IEPTB (JIPMER vs AIIMS)** | 0.380 | 0.777 | 0.454 | 0.819 | 0.689 | 0.318 | 0.814 | 0.814 | 0.584 | 0.621 | 0.593 | 0.748 | 0.360 | 0.727 | 0.315 | 0.748 | 0.859 |

**Table S1.7** **ROC/AUC values from pairwise comparisons for single biomarkers between control and latent progressor and non-progressor**

**groups**

| **Group** | **Biomarker ROC Curve Value** | | | | | | | | | | | | | | | | |
| --- | --- | --- | --- | --- | --- | --- | --- | --- | --- | --- | --- | --- | --- | --- | --- | --- | --- |
|  | **CD52** | **GBP1** | **GBP2** | **GBP5** | **HLA-B** | **IFIT3** | **IFITM3** | **IRF1** | **LOC400759** | **NCF1C** | **PF4V1** | **S100A11** | **SAMD9L** | **STAT1** | **TAF10** | **TAPBP** | **TRIM25** |
| **CNTRLA vs**  **LTBI-NPR** | ***0.133*** | 0.821 | 0.577 | 0.465 | 0.593 | 0.894 | **0.978** | **0.909** | 0.828 | 0.793 | ***0.250*** | **0.984** | 0.516 | 0.627 | ***0.206*** | 0.542 | 0.638 |
| **CNTRLA vs**  **LTBI-PR** | ***0.144*** | 0.806 | 0.513 | 0.638 | 0.575 | 0.888 | **0.981** | 0.875 | 0.813 | 0.756 | ***0.288*** | **0.976** | 0.525 | 0.628 | ***0.274*** | 0.405 | 0.744 |
| **CNTRLB vs**  **UKLTBI-NPR** | 0.459 | 0.797 | 0.524 | 0.535 | 0.587 | 0.599 | 0.724 | 0.493 | 0.599 | 0.446 | 0.510 | 0.408 | 0.685 | 0.590 | 0.606 | 0.402 | 0.466 |
| **PCNTRLB vs**  **LTBI-PR** | 0.536 | 0.780 | 0.472 | 0.694 | 0.597 | 0.586 | 0.755 | 0.449 | 0.591 | 0.425 | 0.487 | 0.316 | 0.623 | 0.629 | 0.543 | ***0.235*** | 0.571 |
| **LTBI-NPR vs**  **LTBI-PR** | 0.622 | 0.528 | 0.451 | 0.668 | 0.361 | 0.476 | 0.470 | 0.460 | 0.508 | 0.475 | 0.476 | 0.383 | 0.583 | 0.572 | 0.439 | 0.340 | 0.628 |
