# Supplementary figures and images for: Validation of Differentially Expressed Immune Biomarkers in Latent and Active Tuberculosis by Real-Time PCR
Source: Front Immunol. 2021 Mar 16;11:612564. doi: 10.3389/fimmu.2020.612564 (PMC8029985; doi:10.3389/fimmu.2020.612564)

## Slide 1
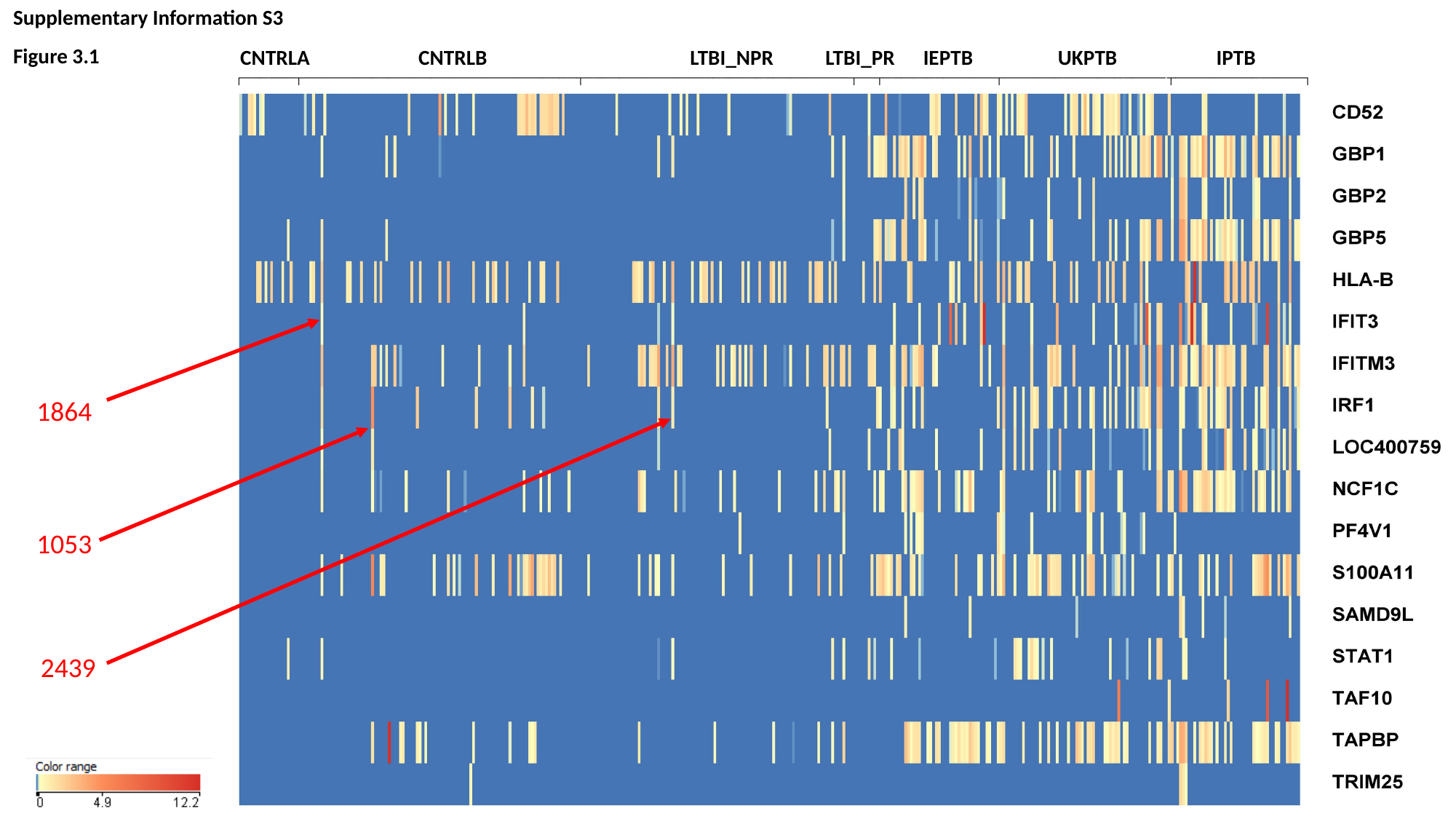

Supplementary Information S3
Figure 3.1
 CNTRLA CNTRLB LTBI_NPR LTBI_PR IEPTB UKPTB IPTB ┌──────┬────────────────────────────────┬───────────────────────────────┬──┬─────────────┬───────────────────┬───────────────┐
1864
1053
2439

Supplement: Supplementary Table S1 — Table 1.1. Details of Patient and Control Samples Table 1.2 Summary of numbers of patients per group and affiliations Table 1.2. Summary of patients or controls recruited per collaborating site used in the study Table 1.3. Number of participants per PREDICT TB LTBI and CNTRLB study groups study groups classified by TST IGRA status and progression to active TB Table 1.4 Gene entities validated in study using Roche Real-time Ready qPCR assays with assay configuration identifiers and ascribed biological function Table 1.5 Summary of the differentially expressed gene entities between the control, latent and active TB disease groups in the study from ANOVA SNK analysis Table 1.6 ROC/AUC values from pairwise comparisons for single biomarkers between control, latent and active TB disease groups Table 1.7 ROC/AUC values from pairwise comparisons for single biomarkers between control and latent TB progressor and non-progressor groups. [file DataSheet_1.zip › Supplementary Information S3.pptx]
